# *Csf1r* mediates enhancement of intestinal tumorigenesis caused by inactivation of *Mir34a*

**DOI:** 10.7150/ijbs.75503

**Published:** 2022-08-29

**Authors:** Fangteng Liu, Nassim Bouznad, Markus Kaller, Xiaolong Shi, Janine König, Stephanie Jaeckel, Heiko Hermeking

**Affiliations:** 1 Experimental and Molecular Pathology, Institute of Pathology, Ludwig-Maximilians-University München, Germany; 2 German Cancer Consortium (DKTK), D-69120 Heidelberg, Germany; 3 German Cancer Research Center (DKFZ), D-69120 Heidelberg, Germany

**Keywords:** *MiR34a*, * miR-34a*, *CSF1R*, * Csf1r*, *APC*, p53, intestinal adenomas, colorectal cancer, *Ntn1*, * Tagln*

## Abstract

The CSF1 receptor (CSF1R) encoding mRNA represents a direct target of miR-34a. However, the *in vivo* relevance of the suppression of *CSF1R* by miR-34a for intestinal tumor suppression mediated by the p53/miR-34a pathway has remained unknown. Here, *Apc*^Min/+^ mice with intestinal-epithelial cell (IEC)-specific deletions of *Mir34a* showed increased formation of adenomas and decreased survival, whereas deletion of *Csf1r* decreased adenoma formation and increased survival. In adenomas deletion of *Mir34a* enhanced proliferation, STAT3 signaling, infiltration with fibroblasts, immune cells and microbes, and tumor stem cell abundance and decreased apoptosis. Deletion of *Csf1r* had the opposite effects. In addition, homeostasis of intestinal secretory and stem cells, and tumoroid formation were affected in opposite directions by deletion of *Mir34a* and *CSF1R*. Concomitant deletion of *Csf1r* and *Mir34a* neutralized the effects of the single deletions. mRNAs containing Mir34a seed-matching sites, which encode proteins related to EMT (epithelial-mesenchymal transition), stemness and Wnt signaling, were enriched after *Mir34a* inactivation in adenomas and derived tumoroids. *Netrin-1/Ntn1* and *Transgelin/Tagln* were characterized as direct targets of Mir34a and Csf1r signaling. *Mir34a*-inactivation related expression signatures were associated with CMS4/CRISB+D, stage 4 CRCs and poor patient survival. In tumoroids the loss of *Mir34a* conferred resistance to 5-FU which was mediated by *Csf1r*. This study provides genetic evidence for a requirement of Mir34a-mediated *Csf1r* suppression for intestinal stem/secretory cell homeostasis and tumor suppression, and suggests that therapeutic targeting of CSF1R may be effective for the treatment of CRCs with defects in the p53/miR-34a pathway.

## Introduction

Colorectal cancer (CRC) arises through a multistep process in which genetic and epigenetic alterations accumulate in a sequential order. CRC is caused by the sequential inactivation of tumor suppressor genes (*APC, DCC, DPC4/SMAD4, TP53*) and activation of oncogenes, such as *KRAS* 1. Activation of the Wnt signaling pathway by mutations of the *Adenomatous polyposis coli (APC)* or the *CTNNB1* gene is a hallmark of CRCs 2.

The *miR-34a* gene is directly activated by p53 and has tumor suppressive functions 3-7. Numerous mRNAs directly targeted by miR-34a have been identified, which mediate its effects 8-10. The* miR-34a* gene is frequently inactivated by epigenetic silencing in CRCs and other tumor entities 11, 12. Whereas, inactivation of the *Mir34a* and/or *Mir34b/c* genes in mice does not increase the rate of tumor formation, combination of *Mir34* loss with other pro-tumorigenic lesions or treatments promotes tumor formation and progression in mouse models of prostate 13, pancreatic 14 and lung cancer 15 as well as in sporadic 16, colitis-associated 17 and inherited colon cancer mouse models 18. Since loss of *Mir34a* did not promote lymphoma formation in *Eµ-Myc* mice 19 and the ability of miR-34a to inhibit proliferation may depend on high levels of expression, the definition of *miR-34a* as *bona fide* tumor suppressor has been subject to debates 20. However, besides its effects on proliferation miR-34a has been implicated in the negative regulation of numerous other pro-tumorigenic processes, such as autophagy and metabolism, by targeting key regulators and effectors of these processes 3-7. Therefore, miR-34a presumably harbors context-dependent, tumor suppressive capacities.

Recently, we demonstrated that abrogation of the miR-34-mediated suppression of CSF1R/STAT3 signaling promotes EMT and associated processes, such as migration, invasion, metastasis and chemo-resistance of human colorectal cancer cell lines 21. In primary CRCs elevated expression of CSF1R was detected at the tumor invasion front and was associated with CpG methylation of the *miR-34a* promoter as well as distant metastasis 21.

Here, we aimed to determine the *in vivo* role of *Csf1r* as a *Mir34a* target in a preclinical mouse model of CRC using a genetic approach. Therefore, we generated *Apc*^Min/+^ mice carrying intestinal-epithelial cell (IEC)-specific deletions of the *Mir34a* or *Csf1r* loci and combinations of these. Here we report the characterization of the resulting phenotypes at the organismal and molecular level. Our findings provide genetic proof of an important role of *Csf1r* down-regulation for tumor suppression by *Mir34a* during intestinal tumorigenesis.

## Materials and Methods

### Generation and breeding of mice

Conditional *Csf1r* gene knockout mice (*Csf1r^fl/fl^*), in which exon 5 of the *Csf1r* gene is flanked by *loxP* sites 22, were purchased from the Jackson Laboratory (*Csf1r^flox^*, Stock No: 021212). The generation of *Mir34a^fl/fl^* mice was described previously 17. To delete *Csf1r* and *Mir34a* in intestinal epithelial cells (IECs), *Csf1r^fl/fl^* and *Mir34a^fl/fl^* mice were crossed with *Villin-Cre* mice with a C57BL/6 background 10, 22-24. The offspring showed no overt phenotype and the genotypes were obtained in normal Mendelian ratios. *Csf1r^ΔIEC^*, *Mir34a^ΔIEC^*,* Csf1r^fl/fl^;Mir34a^fl/fl^* and* Csf1r^ΔIEC^;Mir34a^ΔIEC^* mice were crossed with *Apc*^Min/+^ mice with a C57BL6 background*.* Mice were housed in individually ventilated cages (IVC) with a 12 hour light/dark cycle and *ad libitum* access to water and standard rodent diet. Animal experimentation was approved by the Government of Upper Bavaria. Oligonucleotides used for genotyping are listed in Supplementary Table S1.

### Tissue preparation and adenoma counting

The whole murine intestinal tract was isolated and flushed with PBS. The small intestine was separated into three equal parts (duodenum, jejunum and ileum). The murine intestinal tract was opened longitudinally and rolled to form a “swiss roll”. Each part was photographed, fixed in formalin, dehydrated and embedded into paraffin. Tumor numbers and size were evaluated by using a ZEISS dissecting microscope.

### Histology and Immunohistochemistry

5 μm paraffin sections were used for hematoxylin and eosin (H&E), and periodic acid-Schiff (PAS) staining. Immunohistochemistry (IHC) was conducted using DAB (3,3´-Diaminobenzidine) for brown stainings or aminoethyl carbazole (AEC) for red stainings. Antibodies and reagents are listed in Supplementary Table S2. Slides were scanned (Vectra® Polaris™ Automated Quantitative Pathology Imaging System), and quantified by ImageJ software 18.

### *In situ* hybridization analysis

For detection of intestinal stem cells (ISCs), the *Olfm4* and *Lgr5* RNA probes were generated using the Bluescript II SK plasmid p695-pBS-mOlfm4 (kindly provided by Prof. Hans Clevers), and the pBSII KS-mLgr5 plasmid. The Lgr5 open reading frame (ORF) was obtained from the pCMV6 entry plasmid (bought from OriGene Technologies, Inc., catalog number MR219702), and cloned into the Bluescript II (pBSII) KS plasmid by using the restriction enzymes *NotI* and *KpnI*. For the generation of the probes the Bluescript II plasmid p695-pBS-mOlfm4 and the pBSII KS-mLgr5 were linearized with *NotI* and *EcoRI* restriction enzymes, respectively. The *Olfm4* and *Lgr5* RNA probes were *in vitro* transcribed with RNA-T7 Polymerase and labeled with digoxigenin (DIG) by using the DIG Northern Starter Kit (Roche Diagnostics). *In situ* hybridization (ISH) was performed as described 25. For visualization of bacterial infiltration in intestinal adenoma, the universal eubacteria probe (EUB338) and negative probe (NON338) were used for fluorescence *in situ* hybridization (FISH) as previously described 16. The oligonucleotides used for FISH are listed in Supplementary Table S3.

### Generation of cell pools stably expressing conditional alleles

Stably transfected cells were generated as described previously 24. Briefly, cells were transfected with pRTR plasmids 26 using Lipofectamin 2000 (Invitrogen) or FuGENE6 (Roche). After 24 hours, cells were transferred into media containing 4 μg/ml puromycin for one week. Homogeneity of the derived cell pools was tested by addition of 100 ng/ml DOX for 48 hours, and GFP expression was evaluated by fluorescence microscopy.

### Cell lines and tumoroid culture

The lung cancer cell line H1299 and colorectal cancer cell line SW480 were kept in Dulbecco's modified Eagle's medium (DMEM), murine colorectal carcinoma cell CT26 was cultured in RPMI1640 (Sigma-Aldrich, Gibco Life Technologies). Intestinal adenoma cells from three tumors for each *Apc*^Min/+^ mouse were isolated by lysis in DMEM containing 4000 units Collagenase Type IV (Merck Millipore) and 125 μg/ml Dispase Type II (Sigma-Aldrich). 1.5×10^4^ single cells were counted using a hemocytometer and then embedded in 50 μl Matrigel per well and seeded in 24-well plates. Tumoroids were documented with a Nikon AZ-100 macroscope. Tumoroid passaging and culture medium formulation was done as described before 24, 27.

### Immunofluorescence (IF) staining of tumoroids

Organoids were harvested for immunofluorescence staining as previously described 28, 29. Briefly, Matrigel drops containing tumoroids were collected in 15 ml centrifuge tubes and washed with cold HBSS three times gently. Tumoroids were settled by gravity. Then tumoroids were fixed with 4% paraformaldehyde (PFA) for 30 minutes, permeabilized with 0.1% Triton X-100 for 30 minutes, and then pre-blocked with HBSS containing 5% BSA for 1 hour at room temperature. The antibodies used for immunofluorescence staining are listed in Supplementary Table S2. For EdU staining an EdU Click 555 Kit (baseclick GmbH) was used according to the manufacturer's protocol. The images of tumoroids were acquired with an LSM 700 confocal microscope (Zeiss).

### Dual 3'-UTR luciferase reporter assays

The 3'-UTR of the murine *Csf1r*, *Tagln* and *Ntn1* mRNA was PCR amplified from cDNA obtained from murine bone-marrow derived macrophages or murine genomic DNA. The 3'-UTRs of the human *TAGLN* and *NTN1* mRNA were PCR amplified from cDNA obtained from SW480, inserted into the pGL3-control-MCS vector, and verified by sequencing. The mutagenesis of the miR-34a seed-matching sequences was performed with the QuikChange II XL Site-Directed Mutagenesis Kit (Stratagene) or Mut Express II Fast Mutagenesis Kit V2 (Vazyme Biotech Co.,Ltd) and verified by sequencing. H1299 or CT-26 cells were seeded in 12-well plate with 3×10^4^ cells/well for 24 hours, and transfection with 100 ng of the indicated firefly luciferase reporter plasmid, 20 ng of Renilla reporter plasmid for normalization, and 25 nM of *pre-miR-34a* oligonucleotide (Ambion, PM11030), or a negative control oligonucleotide (Ambion, neg. control #1) with HiPerFect Transfection Reagent (Qiagen). 48 hours later, luciferase assays were performed using a Dual Luciferase Reporter assay (Promega). Fluorescence intensities were measured with an Orion II Luminometer (Berthold) and analyzed with the SIMPLICITY software package (DLR). The oligonucleotides and the primers used for cloning and mutagenesis are listed in Supplementary Table S4.

### Quantitative real-time PCR

Total RNA was isolated using the RNeasy Plus Mini Kit (Qiagen) or High Pure RNA Isolation Kit (Roche), and converted to cDNA using the Verso cDNA Kit (Thermo Scientific). Quantitative real-time PCR (qPCR) was conducted with Fast SYBR Green Master Mix (Applied Biosystems) on the LightCycler 480 II platform (Roche Diagnostics). Expression was normalized to *Cyclophilin*, *B2M* or *β-actin* expression and calculated using the 2^-ΔΔCt^ method 30. Primers used for qPCR are listed in Supplementary Table S5.

### Western blot analysis

Cell-lysates were collected in RIPA lysis buffer (50 mM Tris/HCl, pH 8.0, 250 mM NaCl, 1% NP40, 0.5% (w/v) sodium deoxycholate, 0.1% sodium dodecylsulfate, protease inhibitor cocktail tablets (Roche) and phosphatase inhibitor cocktail tablets (Roche)), and then sonicated and centrifuged at 12000 rpm for 20 min at 4°C. 40-80 μg protein were separated on 12% sodium dodecyl sulfate-acrylamide gels and transferred to Immobilon-P transfer membranes (R1JB33689; Merck Millipore). Signals from horseradish-peroxidase-coupled secondary antibodies were generated by enhanced chemiluminescent substrate (WBKLS0100; Merck Millipore) and recorded with a CCD/Charged Coupled Device camera (Odyssey Fc; LI-COR, Lincoln, NE). Densitometric analysis of blots was performed using Image Studio Version 5.2 software. Antibodies used here are listed in Supplementary Table S2.

### Transcriptomic analysis

Total RNA from adenomas or tumoroids was isolated using the RNeasy Plus Mini Kit (Qiagen), with an on-column DNase digestion (3 RNA samples per genotype; each tumor RNA sample represented a pool of 3 tumors isolated from the same mouse). Random primed cDNA libraries were constructed and sequenced using the HiSeq4000 (Illumina) platform by GATC (Konstanz, Germany). Each sample was covered by at least 30 million single reads of 50 bp length. RNA-Seq FASTQ files were processed using the RNA-Seq module implemented in the CLC Genomics Workbench v20.0.2 software (Qiagen Bioinformatics) and mapped to the GRCm38/mm10 mouse reference genome and its associated gene and transcript annotation (ENSEMBL) using the settings mismatch cost = 2, insertion cost = 2, deletion cost = 3, length fraction = 0.8, similarity fraction = 0.8. RNA-Seq data were filtered to exclude weakly expressed transcripts with less than two mapped exon reads in all samples from the analysis and subjected to upper quartile normalization using the R/Bioconductor RUVSeq (remove unwanted variation from RNA-Seq data) package as described in Risso et al. 31. Differential gene expression analysis was performed with DESeq2 32 after normalization using the RUVg approach to remove variation between RNA samples resulting from differences in library preparation. Principal Component Analysis (PCA) was performed using the PCA functionality of the EDASeq R package as implemented in RUVSeq. Gene Set Enrichment Analysis (GSEA) was performed with the fgsea R package 33. Prior to GSEA, expression changes from low count genes were adjusted using the ashr (adaptive shrinkage) estimator 34. Heatmaps were generated with Morpheus (Broad Institute). Gene sets were obtained from the Molecular Signatures database (MSigDB) 35 or as indicated 36-40. miR-34a targets were predicted with TargetScanMouse 7.1 41 and 11 additional miRNA target prediction algorithms, which are included in the miRWalk2.0 archive 42.

### Analysis of expression and clinical data from public databases

For the generation of STAT3 and c-JUN expression signatures, we compiled RNA-Seq datasets from the NCBI Gene Expression Omnibus (GEO), as described recently 43. Differentially regulated genes were identified using RNA-Seq data from cell lines/tissues with STAT3, c-JUN, or SRF ectopic expression or knockdown (KD)/knockout (KO). A list of analyzed RNA-Seq studies is provided in Supplementary Table S6-9 with the respective GEO accession numbers. Direct regulation was assessed by analysis of transcription factor ChIP-Seq data using the Cistrome database 44. Only ChIP-Seq data sets that passed peak quality controls were included in the analysis. A list of ChIP-Seq studies analyzed is provided in Supplementary Table S6-9 with the respective GEO accession numbers.

For the analysis of human colon cancer samples, we retrieved expression and clinical data from the TCGA-COAD and GSE39582 patient cohorts 45, 46. Association of patient samples with the different CMS categories was obtained from the Colorectal Cancer Subtyping Consortium (CRCSC) at www.synapse.org. The classifications of tumor samples by CRC intrinsic subtypes (CRIS) were obtained from 47. Expression data of human CRC cell lines were obtained from the Cancer Cell Line Encyclopedia (CCLE) 48. The statistics for Forest plots and survival curves was calculated by log-rank test. For binary classification of cases (high/low expression), the Survminer R-package (https://CRAN.R-project.org/package=survminer) was used to determine optimal cutoff values. Differential expression between tumors of different stages was calculated using one-way ANOVA with a post-test for linear trend from stage 1 to stage 4. Gene Set Enrichment Analysis (GSEA) of curated gene sets obtained from the Molecular Signatures database (MSigDB) 35, or as indicated 43, 49, was performed on pre-ranked gene lists ordered by expression correlation coefficients (Pearson) with the *Δ34a* expression signature, mature miR-34a, or *CSF1R* expression, as described previously 21. The significance of enrichments is presented by normalized enrichment scores (NES) and false discovery rate-adjusted q values.

### Statistical analyses

The GraphPad Prism 8.3.0 software was used for statistical analyses. The statistical significance of differences between group means was determined with the two-tailed unpaired Student's t test and one-way ANOVA. Kaplan-Meier curves were used to display the overall survival time and the results were compared with a log-rank test. *P* values less than 0.05 were considered as statistically significant with asterisks indicated (*P < 0.05, **P < 0.01, ***P <0.001, or ****P < 0.0001).

## Results

### Combined deletion of *Mir34a* and* Csf1r* in murine intestinal epithelium

**Figure 1:**
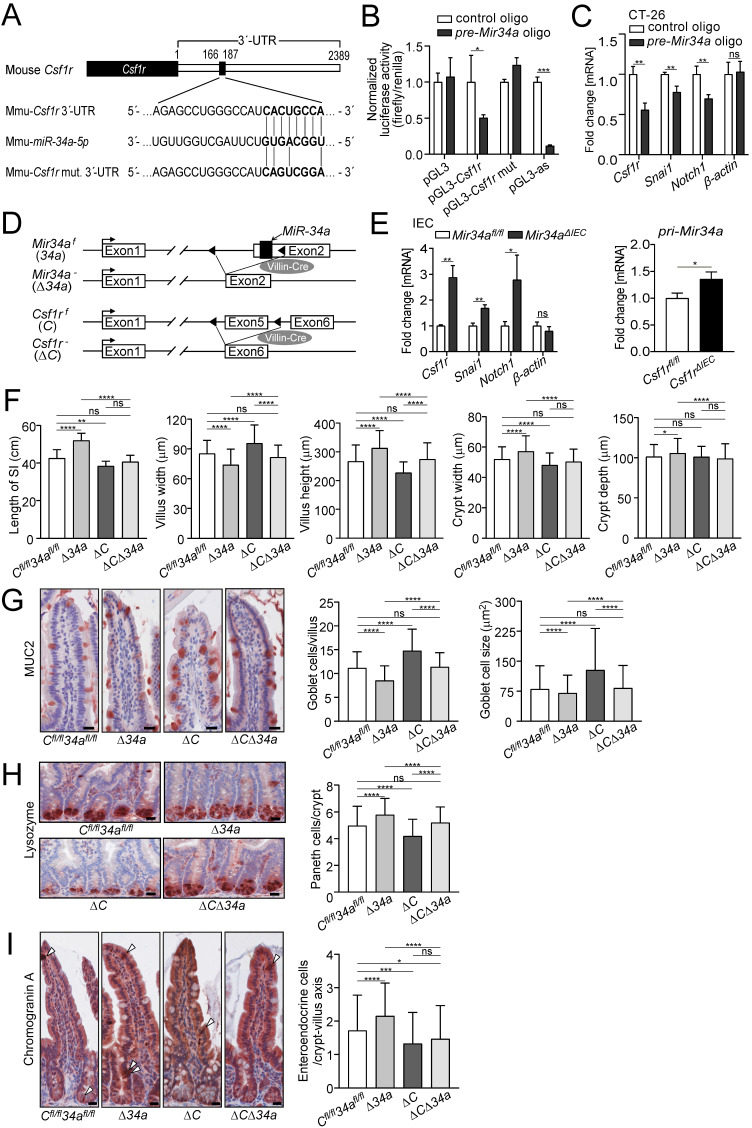
** Analysis of the *Csf1r/Mir34a* connection in mice. A** Scheme of the murine miR-34a seed, the seed-matching sequence and its targeted mutation in the 3'-UTR of the murine *Csf1r* mRNA. **B** Dual-reporter assay after transfection of CT26 cells with *pre-Mir34a* oligonucleotides and the murine *Csf1r* 3'-UTR reporter constructs. Data represent mean ± SD (n = 3). **C** qPCR analysis of the indicated mRNAs in CT26 cells after transfection with control or *pre-Mir34a* oligonucleotides for 48 hours. Data represent mean ± SD (n = 3). **D** Scheme showing the generation of mice with intestinal epithelial cell-specific deletions. **E** Analysis of the indicated mRNAs in intestinal epithelial cells (IECs) derived from the mice with the indicated genotypes. Data represent mean ± SD (n≥3). **F** Determination of the length of the small intestine, and the width and height of villi and the width and depth of crypts in the small intestine from *Apc*^Min/+^ mice with the indicated genotypes (≥ 160 ileum villi or crypts per group and n ≥ 4 mice per genotype). **G** Quantification of goblet cell number and size from the *Apc*^Min/+^ mice with the indicated genotypes after anti-MUC2 staining (≥ 200 villi and ≥ 3000 goblet cells per group, and n ≥ 3 mice per genotype). Scale bar: 20 μm. **H** Quantification of Paneth cell number per crypt from the *Apc*^Min/+^ mice with the indicated genotypes after staining with a Lysozyme-specific antibody (≥ 300 crypt bases per group and n ≥ 3 mice per genotype). Scale bar: 20 μm. **I** Quantification of entero-endocrine cells per crypt-villus axis from the *Apc*^Min/+^ mice with the indicated genotypes after staining with Chromogranin A specific antibodies (≥ 150 crypt-villus axes per group and n ≥ 3 mice per genotype). Scale bar: 20 μm. In (**B,C,E**), results are presented as mean ± SD using the two-tailed unpaired Student's t test. In (**F-I**), results are presented as mean ± SD using Tukey's multiple comparisons test. *P < 0.05, **P < 0.01, ***P <0.001, or ****P < 0.0001.

Similar to the human *CSF1R* the 3'-UTR of the murine *Csf1r* contains a conserved *Mir34a* seed-matching sequence (SMS) (Fig. 1A). In a reporter assay the murine *Csf1r* 3'-UTR was repressed by ectopic *Mir34a*, whereas a reporter with point mutations in the *Mir34a* SMS was refractory to repression by ectopic *Mir34a* in murine CT26 CRC cells (Fig. 1B). In addition, expression of the endogenous *Csf1r* was repressed by ectopic *pre-miR-34a* in murine CT26 cells, similar to the known miR-34a targets *Snai1* and *Notch1*, whereas *β-actin* expression was not affected (Fig. 1C). Therefore, *Csf1r* represents a direct, conserved target for repression by *Mir34a* in mice.

Next, we crossed mice harboring either *Mir34a* or *Csf1r* alleles flanked by two *loxP* sites with *Villin-Cre* mice (Fig. 1D). As a result, the respective alleles were inactivated in intestinal epithelial cells (IECs) from embryonic day 12.5 onwards. In IECs deficient for *Mir34a*, *Csf1r* expression, as well as expression of the known miR-34a targets *Snai1* and *Notch1*, was up-regulated, whereas *β-actin* expression was not affected (Fig. 1E). In addition, *pri-Mir34a* expression was up-regulated in *Csf1r*-deficient IECs (Fig. 1E). Therefore, the reciprocal repression between *miR-34a* and *CSF1R* previously detected in CRC cell lines was confirmed on the organismal level.

### *Csf1r* mediates effects of *Mir34a* loss on intestinal architecture and secretory cell homeostasis

In order to determine, whether inactivation of *Mir34a* affects intestinal tumor formation in a *Csf1r*-dependent manner, we generated *Apc*^Min/+^ mice with inactivation of *Mir34a*, *Csf1r* or of both genes in IECs. In *Mir34a*-deficient *Apc*^Min/+^ mice, we observed a significant increase in total length of the small intestine, and in the width and depth of crypts, as well as in the villus height, but a decrease in villus width of the small intestine (Fig. 1F). Deletion of *Csf1r*, except for depth of crypts, had the opposite effect on these parameters in *Apc*^Min/+^ mice. However, combined deletion of *Mir34a* and *Csf1r* had no significant effect on intestinal architecture when compared with the control group (Fig. 1F). The variations in the width of crypts presumably caused the differences in total length of the small intestine observed among the four genotypes. In addition, we evaluated goblet and Paneth cell numbers after immunohistochemical detection using specific markers as well as PAS staining (Fig. 1G and H, Supplementary Fig. S1A and B). A significant decrease in the number of goblet and an increase in Paneth cells was detected in *Mir34a*-deficient intestines. As reported previously 50, inactivation of *Csf1r* resulted in a decreased number of Paneth cells, while the number and size of goblet cells increased. In addition, the number of entero-endocrine cells was increased in *Mir34a*-deficient *Apc*^Min/+^ mice, but decreased in *Csf1r*-deficient *Apc*^Min/+^ mice (Fig. 1I). When both genes were deleted the effects on goblet, Paneth and entero-endocrine cells were neutralized.

### *Mir34a* loss enhances intestinal tumorigenesis in a *Csf1r*-dependent manner

**Figure 2:**
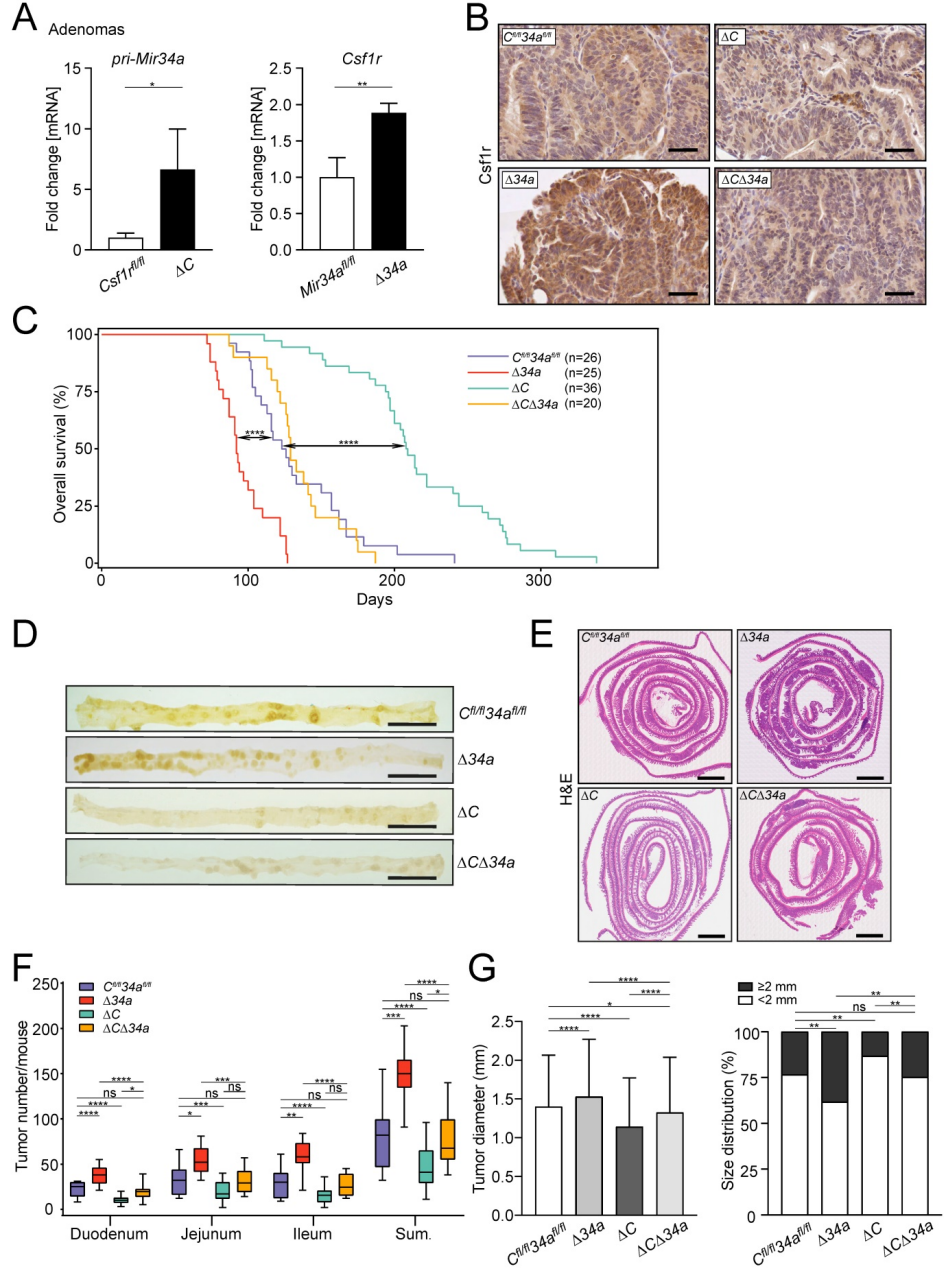
** Effects of *Mir34a* and/or *Csf1r* deficiency on intestinal tumorigenesis in *Apc*^Min/+^ mice. A** qPCR analysis of *Csf1r* and *pri-Mir34a* in adenomas of *Apc*^Min/+^ mice with the indicated genotypes. Results are presented as mean values ± SD (n ≥ 3). **B** IHC detection of Csf1r in intestinal adenomas in *Apc*^Min/+^ mice with the indicated genotypes. Scale bar: 50 μm. **C** Kaplan-Meier survival analysis of the *Apc*^Min/+^ mice with the indicated genotypes. Results were compared with a log-rank test. **D** Representative macroscopic images of polyps in resected small intestines in 18 weeks old *Apc*^Min/+^ mice with the indicated genotypes. Scale bar: 2 cm. **E** Representative “swiss-roll” sections of the small intestine of 18 weeks old *Apc*^Min/+^ mice by H&E staining. Scale bar: 2 mm. **F** Quantification of tumor numbers in the small intestine of 18 weeks old *Apc*^Min/+^ mice with the indicated genotypes. The box plot extends from the 25th to 75th percentiles. The line in the middle of the box indicates the median. The whiskers underneath or above the boxes range from min. to max. value, respectively (n ≥ 7 mice per genotype). **G** Quantification of tumor size and distribution in small intestine of 18 weeks old *Apc*^Min/+^ mice with the indicated genotypes (n ≥ 7 mice per genotype). In (**A**) results are presented as mean ± SD using the two-tailed unpaired Student's t test, in (**G**) results are presented as mean ± SD using Tukey's multiple comparisons test. *P < 0.05, **P < 0.01, ***P <0.001, or ****P < 0.0001.

**Figure 3:**
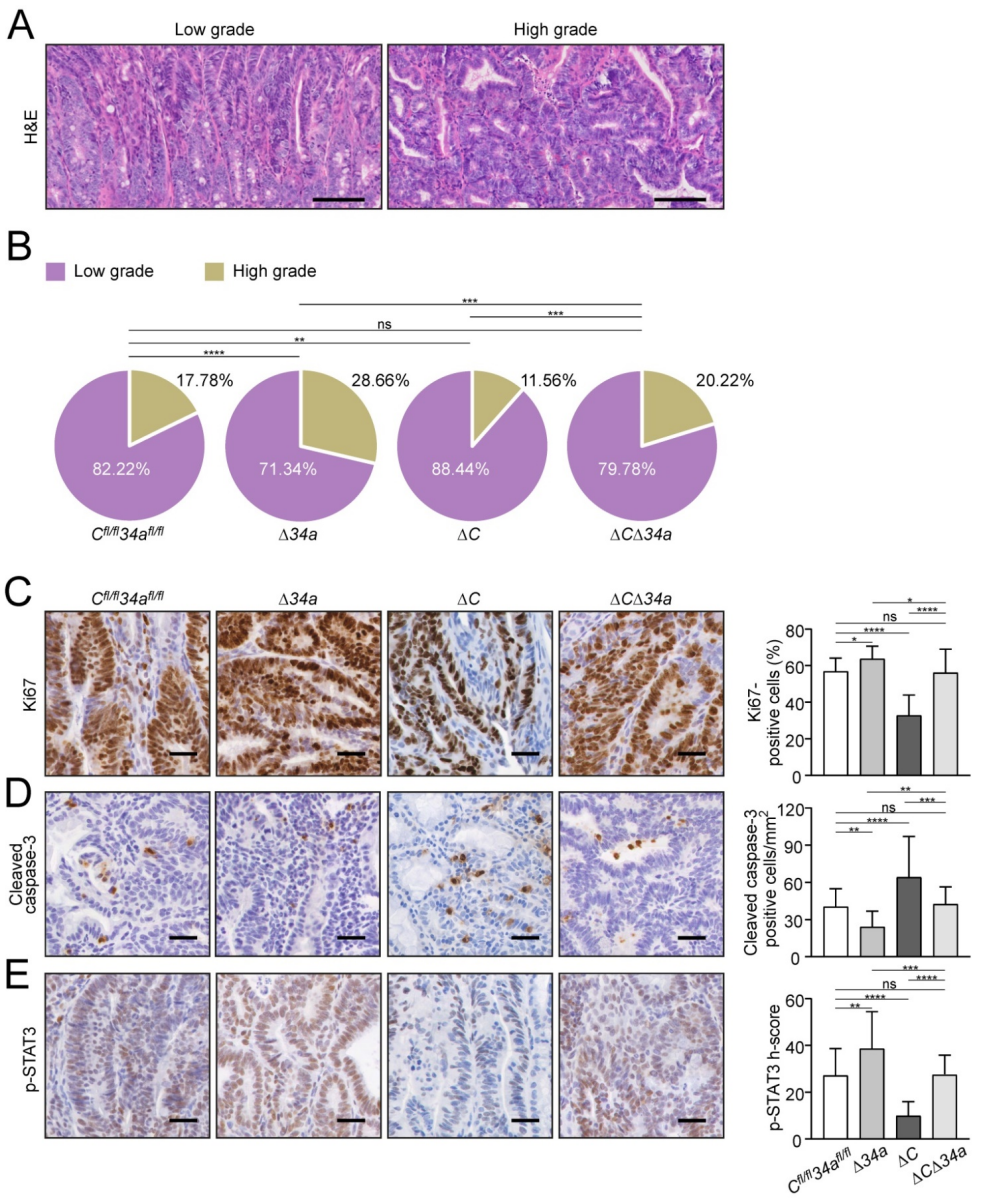
** Cellular effects of *Mir34a* and/or *Csf1r* deficiency in intestinal adenomas. A** Representative images of low- and high-grade adenomas. Scale bar: 100 μm. **B** Quantification of tumor stage in adenomas from the small intestine in 18 weeks old *Apc*^Min/+^ mice with the indicated genotypes (n = 6 mice per genotype). **C, D, E,** IHC detection of Ki67 (**C**), cleaved-caspase-3 (**D**) and phospho-STAT3 (**E**) in adenomas from the small intestine in 18 weeks old *Apc*^Min/+^ mice with the indicated genotypes. (n ≥ 3 mice per genotype). Scale bar: 30 μm. In **(B-E)**, results are presented as mean ± SD using Tukey's multiple comparisons test. *P < 0.05, **P < 0.01, ***P <0.001, or ****P < 0.0001.

The expression of *Csf1r* was up-regulated in *Mir34a*-deficient adenomas and *pri-Mir34a* expression was increased in *Csf1r*-deficient adenomas on the mRNA and protein levels (Fig. 2A and B). Notably, IEC-specific deletion of *Csf1r* in *Apc*^Min/+^ mice resulted in a significantly longer life-span, while loss of *Mir34a* resulted in a shorter overall survival (Fig. 2C). In contrast, *Apc*^Min/+^ mice deficient for both *Csf1r* and *Mir34a* did not show a statistically significant change in survival when compared to *Csf1r^fl/fl^;Mir34a^fl/fl^;Apc*^Min/+^ mice (Fig. 2C). When the entire small intestinal tract from 18 weeks old *Apc*^Min/+^ mice was examined, *Csf1r-*deficient *Apc*^Min/+^ mice showed a significantly reduced number of intestinal tumors, whereas *Mir34a*-deficient mice showed a dramatic increase in tumor numbers (Fig. 2D-F). Notably, *Apc*^Min/+^ mice with deletion of both *Mir34a* and *Csf1r* displayed similar frequencies of intestinal tumors as *Csf1r^fl/fl^;Mir34a^fl/fl^;Apc*^Min/+^ mice. The size of adenomas was significantly larger in *Mir34a*-deficient and smaller in *Csf1r*-deficient *Apc*^Min/+^ mice when compared to *Csf1r^fl/fl^;Mir34a^fl/fl^;Apc*^Min/+^ mice (Fig. 2G). Similarly, the frequency of large tumors (≥ 2 mm) was significantly higher in *Mir34a^ΔIEC^;Apc*^Min/+^ mice*,* but lower in *Csf1r^ΔIEC^;Apc*^Min/+^ mice. However, when *Mir34a* and *Csf1r* were inactivated concomitantly in IECs, the effects of single gene inactivations on tumor size and its distribution were largely neutralized (Fig. 2G). Furthermore, the deletion of *Mir34a* increased the number of tumors with high-grade dysplasia in *Apc*^Min/+^ mice, whereas deletion of *Csf1r* resulted in a lower percentage of tumors with high-grade dysplasia (Fig. 3A and B). However, concomitant deletion of both genes resulted in the compensation of both effects. Taken together, the effects of the single deletions of *Mir34*a and *Csf1r* were neutralized by simultaneous inactivation of both genes, implying that *Mir34a* and *Csf1r* functionally antagonize each other during intestinal tumor formation and progression. Therefore, *Csf1r* up-regulation caused by *Mir34a* deletion contributes to the increased number and size of intestinal adenomas observed in *miR-34a*-deficient mice, which ultimately determines the lifespan of these mice.

We hypothesized that the decreased tumor size observed after deletion of *Csf1r* in *Apc*^Min/+^ mice may be due to decreased tumor cell proliferation and increased apoptosis. Indeed, the proliferation-marker Ki67 was down-regulated and apoptosis was increased in adenomas* of Csf1r^ΔIEC^;Apc*^Min/+^ mice (Fig. 3C and D).

On the contrary, proliferation was increased and apoptosis was decreased in *Mir34a*-deficient adenomas (Fig. 3C and D). When both deletions were combined, the rate of proliferation and apoptosis was similar as in adenomas of *Apc*^Min/+^ mice without deletion of these genes (Fig. 3C and D). Consistent with the finding that activation of CSF1R induces STAT3 phosphorylation (p-STAT3) in CRC cell lines 21, the frequency of cells displaying STAT3 activation was decreased in adenomas of *Csf1r^ΔIEC^;Apc*^Min/+^ mice, whereas deletion of *Mir34a* increased the number of p-STAT3-positive tumor cells (Fig. 3E). *Apc*^Min/+^ mice with combined deletion *Csf1r* and *Mir34a* did not show a significant change in the frequency of p-STAT3-positive cells when compared to control *Apc*^Min/+^ mice.

### *Csf1r* loss largely reversed the effects of *Mir34a* deletion on tumor microenvironment

**Figure 4:**
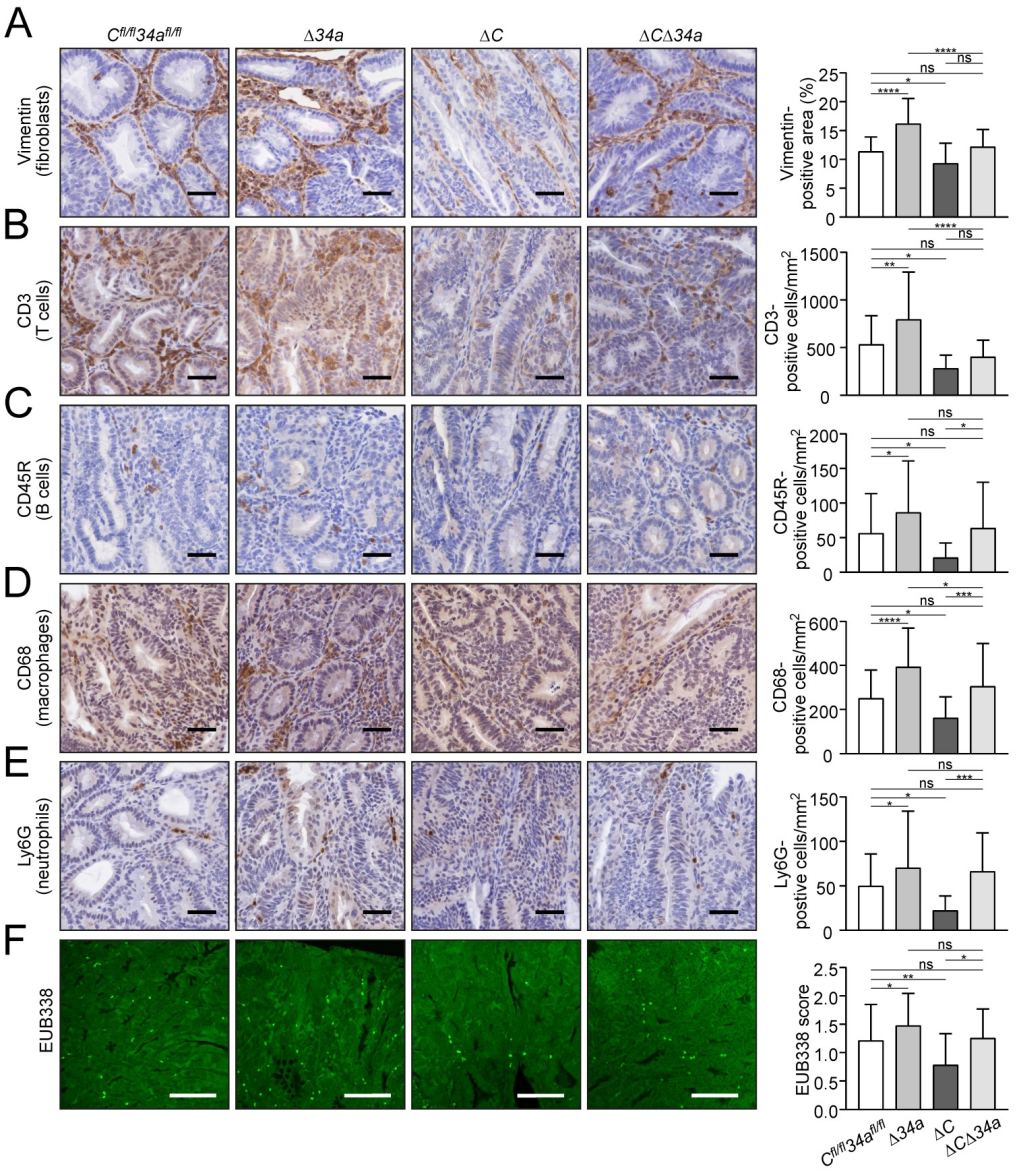
** Effects of* Mir34a* and/or* Csf1R* deficiency on infiltration by stromal cells and bacteria in *Apc*^Min/+^ adenomas. A**,** B**, **C**, **D**, **E** IHC detection of fibroblast cells (**A**) by Vimentin and T cells (**B**), B cells (**C**), macrophages (**D**), neutrophils (**E**) by CD3, CD45R, CD68, Ly6G, respectively, in adenomas. (n ≥ 3 mice per genotype). Scale bar: 40 μm. **F** Quantification of bacterial infiltration using FISH of universal eubacteria probe (EUB338) in adenomas. (n ≥ 3 mice per genotype). Scale bar: 100 μm. In (**A**-**F**) results are presented as mean ± SD using Tukey's multiple comparisons test. *P < 0.05, **P < 0.01, ***P <0.001, or ****P < 0.0001.

Tumor-associated fibroblasts were increased within adenomas in *Mir34a*-deficient adenomas, whereas deletion of *Csf1r* resulted in their decrease (Fig. 4A). Notably, concomitant *Mir34a* and *Csf1r* deletion resulted in unchanged numbers of fibroblasts within adenomas. Therefore, *Csf1r* is required for the recruitment of fibroblasts in *Mir34a*-deficient adenomas. Similarly, *Mir34a* inactivation resulted in a *Csf1r*-dependent increase in CD3-positive T-cells (Fig. 4B), CD45R-positive B-cells (Fig. 4C) and CD68-positive macrophages (Fig. 4D), as well as LY6G-positive neutrophils (Fig. 4E). Deletion of *Csf1r* decreased infiltration by these cell types (Fig. 4A-E). Furthermore, FISH with the universal eubacteria-specific probe (EUB338) revealed that *Mir34a*-deficient adenomas displayed more bacterial infiltration, whereas less bacterial infiltration was observed in *Csf1r*-deficient adenomas (Fig. 4F). The degree of bacterial infiltration was similar in adenomas with deletion of both genes when compared to control mice (Fig. 4F). Taken together, deletion of* Csf1r* largely reversed the effects of* Mir34a* loss on infiltration by fibroblasts, immune cells and bacteria in adenomas. Therefore, the up-regulation of *Csf1r* as a consequence of *Mir34a* inactivation in intestinal adenomas is an important mediator of tumor/stroma interactions, which may promote tumor initiation and progression.

### Role of* Csf1r* in *Mir34a*-loss induced stemness and Wnt signaling

**Figure 5:**
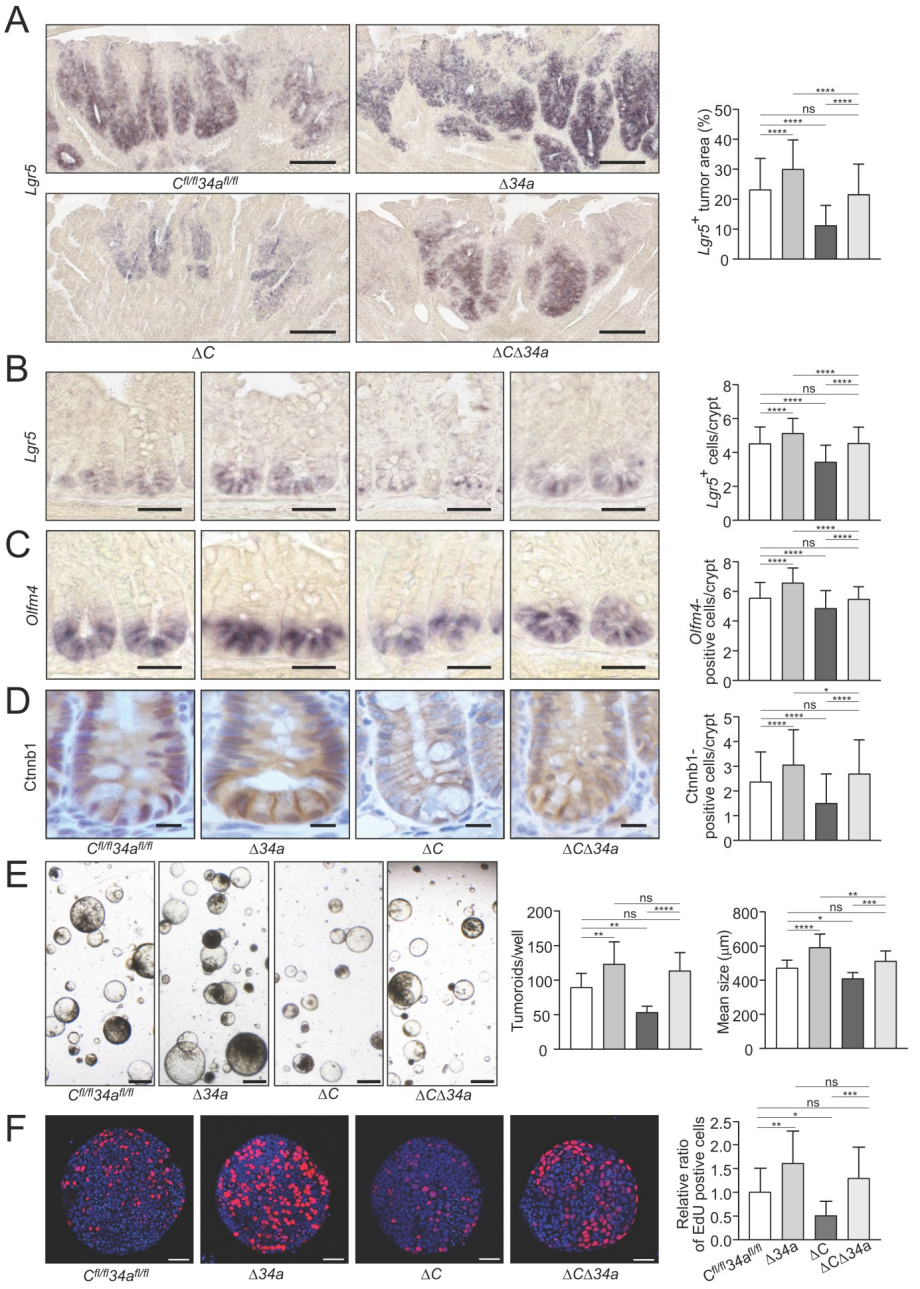
** Effects of* Mir34a and/or Csf1r* deficiency on stemness, β-catenin localization and tumoroid formation/proliferation. A** Quantification of *Lgr5*-positive area in ≥ 40 intestinal adenomas per group. (n= 4 mice per genotype). Scale bar: 140 μm. **B**, **C** *in situ* hybridization detection of *Lgr5*-positive cells (**B**) and *Olfm4*-positive cells (**C**) at the crypt base of 18 weeks old *Apc*^Min/+^ mice with the indicated genotypes. At least 40 crypts per mouse (n ≥ 3 mice per genotype) were counted. Scale bar: 40 μm. **D** Quantification of β-catenin/Ctnnb1 nuclear positive cells in ≥ 180 normal crypts per group (n= 3 mice per genotype). Scale bar: 15 μm. **E** Tumoroid formation assay of adenomas (three tumors per mouse) derived from *Apc*^Min/+^ mice with the indicated genotypes (n = 3 mice per genotype). Scale bar: 400 μm.** F** Quantification of EdU labeling of proliferating cells in 20 tumoroids derived from adenomas per group (n = 3 mice per genotype), relative ratio was normalized to the corresponding control. Scale bar: 40 μm. In (**A**-**F**) results are presented as mean ± SD using Tukey's multiple comparisons test. *P < 0.05, **P < 0.01, ***P <0.001, or ****P < 0.0001.

In order to assess effects on tumor cell stemness, we determined the expression of the stem cell marker *Lgr5* in adenoma sections using *in situ* hybridization (ISH) (Fig. 5A). *Lgr5*-positive areas were increased in intestinal adenomas of *Mir34a*^ΔIEC^*;Apc*^Min/+^ mice and decreased in adenomas of *Csf1r*^ΔIEC^*;Apc*^Min/+^ mice. However, the combined inactivation of *Mir34a* and *Csf1r* neutralized these effects (Fig. 5A). In addition, a significant increase in the number of ISCs at the crypt base of *Mir34a^ΔIEC^;Apc*^Min/+^ mice was determined by detection of the stem cell markers *Olfm4* and *Lgr5* by ISH, while *Csf1r^ΔIEC^;Apc*^Min/+^ mice showed a decrease in ISCs (Fig. 5B and C). Combined deletion of both genes resulted in an ISC frequency similar to that observed in wild-type *Apc*^Min/+^ mice. Therefore, the enhanced frequency of stem cells observed in *Mir34a*-deficient adenomas and normal intestinal crypts, was dependent on the increased expression of *Csf1r*.

We had previously observed that *miR-34a/b/c*-deletion in combination of hemizygous *APC* inactivation promotes nuclear accumulation of β-catenin 18. In order to assess the effect of the introduced deletions on Wnt signaling the β-catenin localization in the untransformed crypts of the *Apc*^Min/+^ mice with deletions of *Mir34a* or/and *Csf1r* was determined (Fig. 5D). As expected, an increased nuclear accumulation of β-catenin protein in cells at the crypt bases was observed after *Mir34a* deletion in *Apc*^Min/+^ mice. Interestingly, deletion of *Csf1r* decreased the number of cells with nuclear β-catenin at the crypt base and concomitant deletion of *Mir34a* and *Csf1r* resulted in similar numbers of cells with nuclear β-catenin as observed in the controls. Therefore, loss of *Mir34a* contributes to β-catenin activation in a *Csf1r*-dependent manner.

In order to obtain functional evidence for *Mir34a/Csf1r* mediated regulation of stemness in adenomas, we performed a tumoroid formation assay (Fig. 5E). Indeed, tumoroids derived from *Mir34a*-deficient adenomas displayed an increase in formation rate and mean size, whereas tumoroids derived from *Csf1r*-deficient adenomas formed at a decreased rate and were smaller.

When both genes were deleted concomitantly, tumoroids were similar in number and size to *Mir34a/Csf1r*- proficient tumoroids. *Mir-34a*-deficient tumoroids exhibited the highest frequency of actively proliferating cells as evidenced by EdU labeling (Fig. 5F, Supplementary Fig. S2), whereas *Csf1r*-deficient tumoroids showed the lowest rate of proliferation. The combined deletion of both genes nullified the effects of single gene deletions, implying that *Csf1r* is an important mediator of the increased proliferation resulting from *Mir-34a* inactivation. These effects on proliferation presumably explain the observed differences in tumoroid number and size among the genotypes.

### Expression profiling of *Mir34a-* and/or* Csf1r*-deficient adenomas and tumoroids

**Figure 6:**
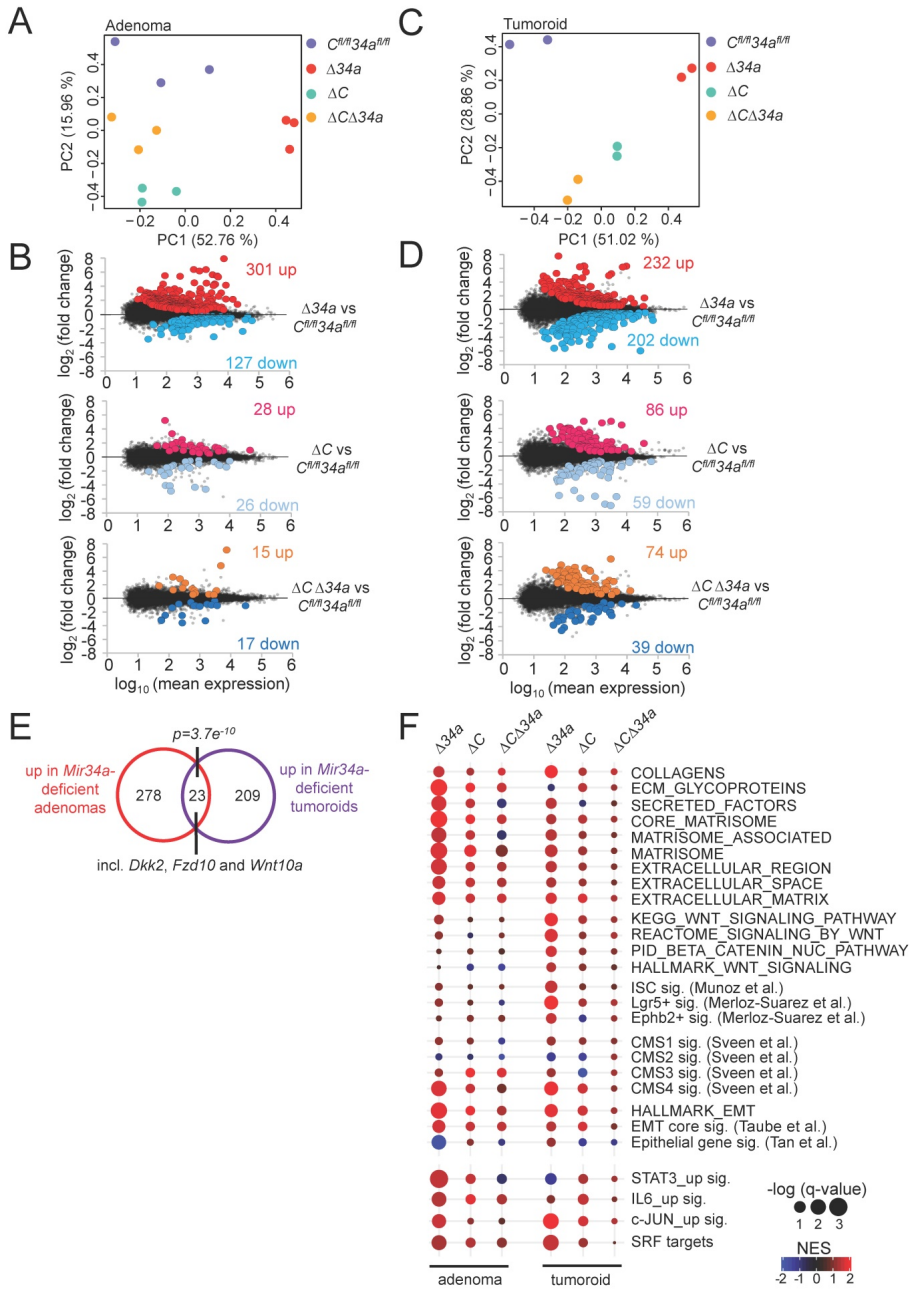
** Expression profiling of *Mir34a- and/or Csf1r*-deficient intestinal adenomas and tumoroids from *Apc*^Min/+^ mice. A** Principal component analysis (PCA) of RNA expression in adenomas from mice with the indicated genotypes. **B** MA-plots showing differential RNA expression (FDR q-value <0.05) between adenomas with the indicated genotypes from *Apc*^Min/+^ mice. Significantly up- and down-regulated RNAs are highlighted as indicated. Non-significantly regulated genes are shown in gray. The numbers of differentially regulated RNAs are indicated. **C** Principal component analysis (PCA) of RNA expression in tumoroids derived from adenomas from mice with the indicated genotypes. **D** MA-plots showing differential RNA expression (FDR q-value <0.05) between tumoroids derived from adenomas of the respective loss-of-function mice and from *Apc*^Min/+^ mice. Significantly up- and down-regulated RNAs are highlighted as indicated. Non-significantly regulated genes are shown in gray. The numbers of differentially regulated RNAs are indicated. **E** Venn diagram showing overlap between RNAs differentially up-regulated in *Mir34a^ΔIEC^;Apc*^Min/+^ adenomas or tumoroids. The numbers of differentially regulated RNAs are indicated. Statistical significance was determined by Fisher´s exact test. **F** Dot plot representation of Gene Set Enrichment Analyses (GSEA) of the indicated functional categories obtained from pair-wise comparisons of *Mir34a^ΔIEC^;Apc*^Min/+^, *Csf1r^ΔIEC^;Apc*^Min/+^, or *Mir34a^ΔIEC^;Csf1r^ΔIEC^;Apc*^Min/+^ with *Mir34a^fl/fl^;Csf1r^fl/fl^;Apc*^Min/+^ adenomas and tumoroids. The significance of enrichments is presented by normalized enrichment scores (NES) and false discovery rate-adjusted q values.

Next, we determined the mRNA expression profiles of *Mir34a*^ΔIEC^, *Csf1r^ΔIEC^* and *Mir34a*^ΔIEC^*;Csf1r^ΔIEC^* adenomas and compared them to *Mir34a^fl/fl^;Csf1r^fl/fl^* adenomas from 18-weeks old in *Apc*^Min/+^ mice. For each genotype, 3 libraries were generated from RNA isolated from adenomas of 3 mice (3 adenomas from each mouse were pooled) and subjected to RNA-Seq analysis with more than 30 million reads per library. Principal component analysis (PCA) showed that adenomas of *Mir34a^ΔIEC^* and *Csf1r^ΔIEC^* mice were indeed characterized by distinct transcriptomes, while the gene expression pattern in *Mir34a^ΔIEC^;Csf1r^ΔIEC^* adenomas was more similar to *Mir34a^fl/fl^;Csf1r^fl/fl^* adenomas (Fig. 6A). Differential gene expression analyses using DESeq2 showed that in adenomas from *Mir34a*-deficient mice, 301 genes were significantly up- and 127 genes were down-regulated when compared to adenomas from control mice (Fig. 6B, Supplementary Table S10). In *Csf1r-*deficient adenomas, rather moderate changes in gene expression with 28 significantly up- and 26 significantly down-regulated genes were observed when compared to adenomas from control mice (Fig. 6B, Supplementary Table S11). However, in adenomas from *Csf1r/Mir34a*-deficient mice *Apc*^Min/+^ mice only 15 genes were significantly up- and 17 genes were down-regulated, indicating that the gene expression changes observed in *Mir34a*-deficient adenomas were largely abrogated by the concomitant deletion of *Csf1r* (Fig. 6B, Supplementary Table S12). In addition, we performed NGS analyses of tumoroids derived from *Mir34a-* and/or *Csf1r*-deficient adenomas in order to identify cell autonomous changes in gene expression, which are not potentially confounded by interactions of tumor cells with the tumor-microenvironment, as in the adenomas. PCA showed that tumoroids of the respective genotypes were characterized by distinct transcriptomes (Fig. 6C). Differential gene expression analyses showed that in tumoroids from *Mir34a*-deficient mice, 232 genes were significantly up- and 202 genes were down-regulated (Fig. 6D, Supplementary Table S13). In tumoroids derived from *Csf1r-*deficient and *Csf1r/Mir34a*-deficient adenomas, moderate transcriptome changes with lower numbers of differentially regulated genes were observed (Fig. 6D, Supplementary Table S14 and Supplementary Table S15).

Interestingly, the overlap between mRNAs up-regulated in *Mir34a*-deficient adenomas and tumoroids, though limited, was statistically highly significant, and among the 23 mRNAs significantly up-regulated in both *Mir34a*-deficient adenomas and tumoroids were three factors involved in Wnt signaling (*Dkk2*, *Fzd10* and *Wnt10a*) (Fig. 6E), suggesting that the tumor cell-autonomous repression of Wnt signaling by miR-34a may be a critical mechanism of miR-34a mediated tumor suppression, as reported previously 51, 52. However, the divergent effects of *Mir34a*-deficiency in adenomas and in tumoroids may in part be due to interactions between* Mir34a*-deficient tumor cells in the adenomas and cells within the tumor microenvironment, such as infiltrating macrophages, which do not occur in tumoroids. In addition, tumoroids are cultured in an artificial matrix, which may not fully represent the *in vivo* environment of tumor cells in adenomas 53, and therefore influence gene expression.

Next we used Gene Set Enrichment Analyses (GSEA) to identify pathways that are differentially regulated in adenomas and tumoroids dependent on their *Mir34a* and *Csf1r* status (Fig. 6F). For this, we focused on processes relevant for tumor progression that are known to be suppressed by miR-34a, such as epithelial-mesenchymal transition (EMT), stemness, and Wnt signaling (Fig. 6F). In *Mir34a*-deficient adenomas, EMT-associated genes were strongly up-regulated. Moreover, extracellular matrix (ECM)-related factors, as well as Consensus Molecular Subtype (CMS) 4-associated genes indicative of mesenchymal tumors 54 were up-regulated (Fig. 6F). In *Mir34a*-deficient tumoroids, gene signatures characteristic for ISCs, Wnt signaling, and, to a lesser extent, EMT and ECM-related gene signatures were up-regulated (Fig. 6F). Remarkably, the up-regulation of factors involved in EMT, stemness, Wnt signaling and extracellular matrix components in *Mir34a*-deficient adenomas and/or tumoroids was largely abrogated by co-deletion of *Csf1r* (Fig. 6F). Deletion of *Csf1r* alone had a very limited effect on the analyzed gene signatures. Taken together, these results imply that the up-regulation of *Csf1r* expression in *Mir34a*-deficient tumors represents a central mediator of the effects of *Mir34a* loss on gene expression in intestinal adenomas and/or tumoroids.

### Analysis of *Mir34a* target expression

Next, we analyzed which Mir34a targets were significantly up-regulated in either *Mir34a*-deficient adenomas and/or tumoroids. For the identification of up-regulated Mir34a targets, we employed the miRNA target prediction tools TargetScanMouse 7.1 and the mirWalk2.0 (mouse) archive.

**Figure 7:**
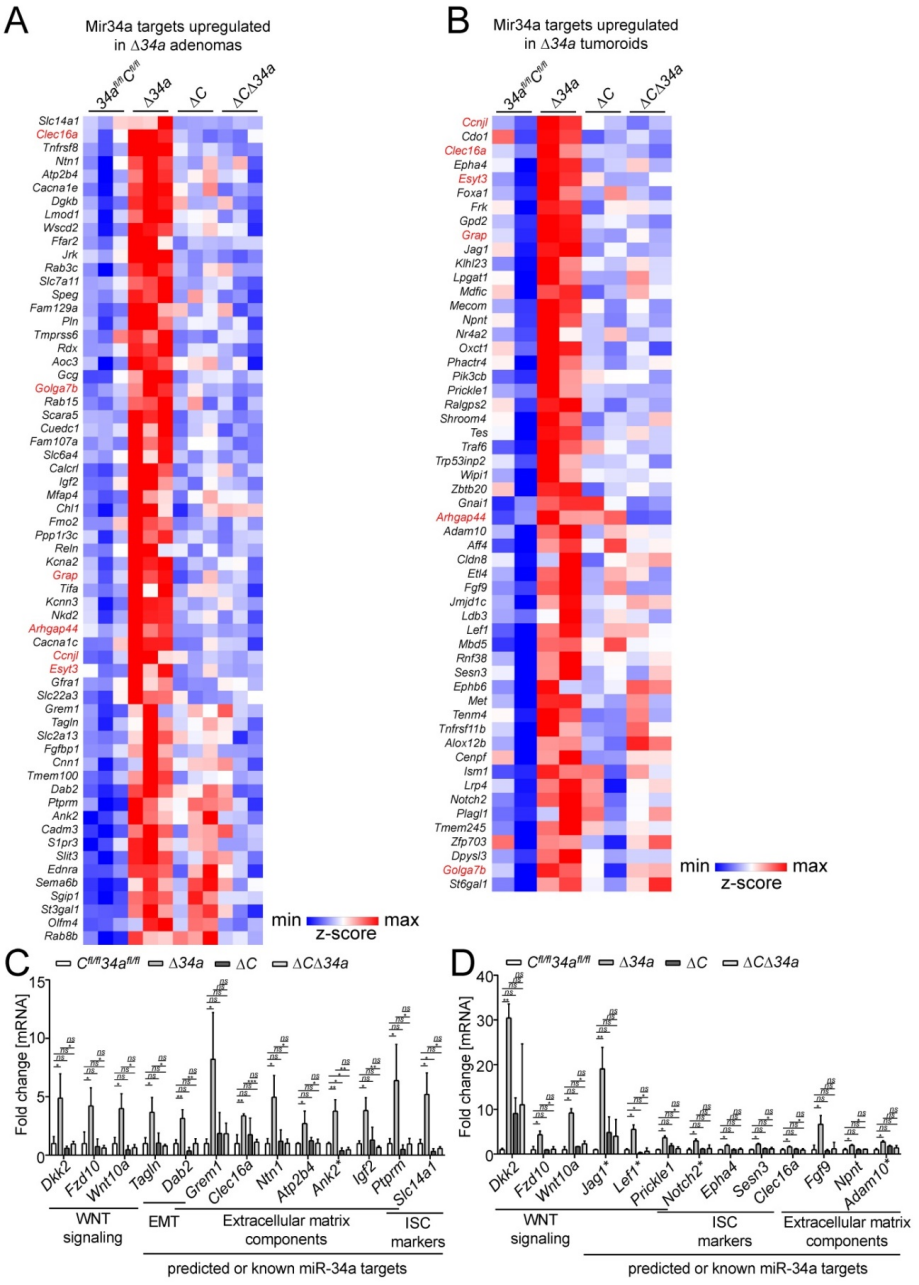
** Identification of Mir34a mRNA targets in *Mir34a- and/or Csf1r*-deficient intestinal adenomas and tumoroids from *Apc*^Min/+^ mice.** Heat-map of RNA expression of predicted Mir34a targets in **A**, adenomas or **B**, tumoroids with the indicated genotypes from* Apc*^Min/+^ mice. RNAs with upregulation in *Δ34a vs 34a^fl/fl^;C^fl/fl^* (FDR<0.05) are shown. RNAs up-regulated both in *Δ34a* adenomas and tumoroids are indicated in red. **C**, **D** Validation of the exemplary Mir34a target genes differently regulated in adenomas (**C**) or tumoroids (**D**) with the indicated genotypes by qPCR. Published miR-34a target are labeled with an asterisk (*). In (**C**, **D**), results are represented as mean ± SD, and data was subjected to an unpaired, two-tailed Student's t-test with *p*-values * < 0.05, ** < 0.01, *** < 0.001, ns: not significant.

In *Mir34a*-deficient adenomas, we identified a set of 62 significantly up-regulated mRNAs with *Mir34a* seed-matching sites in their 3'-UTR (Fig. 7A). In addition, we identified a set of 58 mRNAs with miR-34a seed-matching sites that were up-regulated in tumoroids derived from *Mir34a*-deficient adenomas (Fig. 7B). Among these were mRNAs, which encode factors relevant for the effects of *Mir34a* deletion described above, such as Jag1, Kitl, Lef1, OLFM4, Met and Notch2. Six predicted Mir34a targets (*Arhgap44, Ccnjl, Clec16a, Esyt3, Golga7b, Grap*) were up-regulated in both *Mir34a*-deficient adenomas and tumoroids (Fig. 7A and B). Remarkably, the up-regulation of predicted Mir34a targets in *Mir34a*-deficient adenomas was largely abrogated by co-deletion of *Csf1r* (Fig. 7A). In *Csf1r/Mir34a*-deficient tumoroids, up-regulation of the majority of predicted Mir34a targets was strongly reduced, and only a subset of predicted Mir34a targets was still up-regulated when compared to *Csf1r^fl/fl^; Mir34a^fl/fl^* tumoroids (Fig. 7B). RNA-Seq results were confirmed by qPCR analysis of selected RNAs up-regulated in *Mir34a*-deficient adenomas and/or tumoroids (Fig. 7C and D).

In order to understand the effect of co-deletion of *Csf1r* on *Mir34a* loss induced changes in gene expression, we analyzed if genes up-regulated after inactivation of *Mir34a* are potentially subject to opposing regulation by Mir34a and Csf1r signaling. Activation of Csf1r is known to induce several signaling pathways, such as the JAK-STAT, MAPK and Rho-actin cascades 55, which ultimately result in the activation of several downstream transcription factors (TFs), such as STAT3, AP1 (JUN:FOS) and SRF 56-59. Therefore, we analyzed whether expression signatures comprising RNAs commonly up-regulated after induction of these TFs were associated with loss of *Mir34a* in adenomas and/or tumoroids. Indeed, GSEA showed that loss of *Mir34a* in adenomas, and to a lesser extent in tumoroids, was associated with the induction of STAT3, JUN and SRF expression signatures. Furthermore, loss of *Mir34a* in adenomas was associated with the induction of RNAs commonly up-regulated after IL6 treatment, which includes STAT3 activation (Fig. 6F). Remarkably, this effect was largely abrogated by co-deletion of *Csf1r* (Fig. 6F).

**Figure 8:**
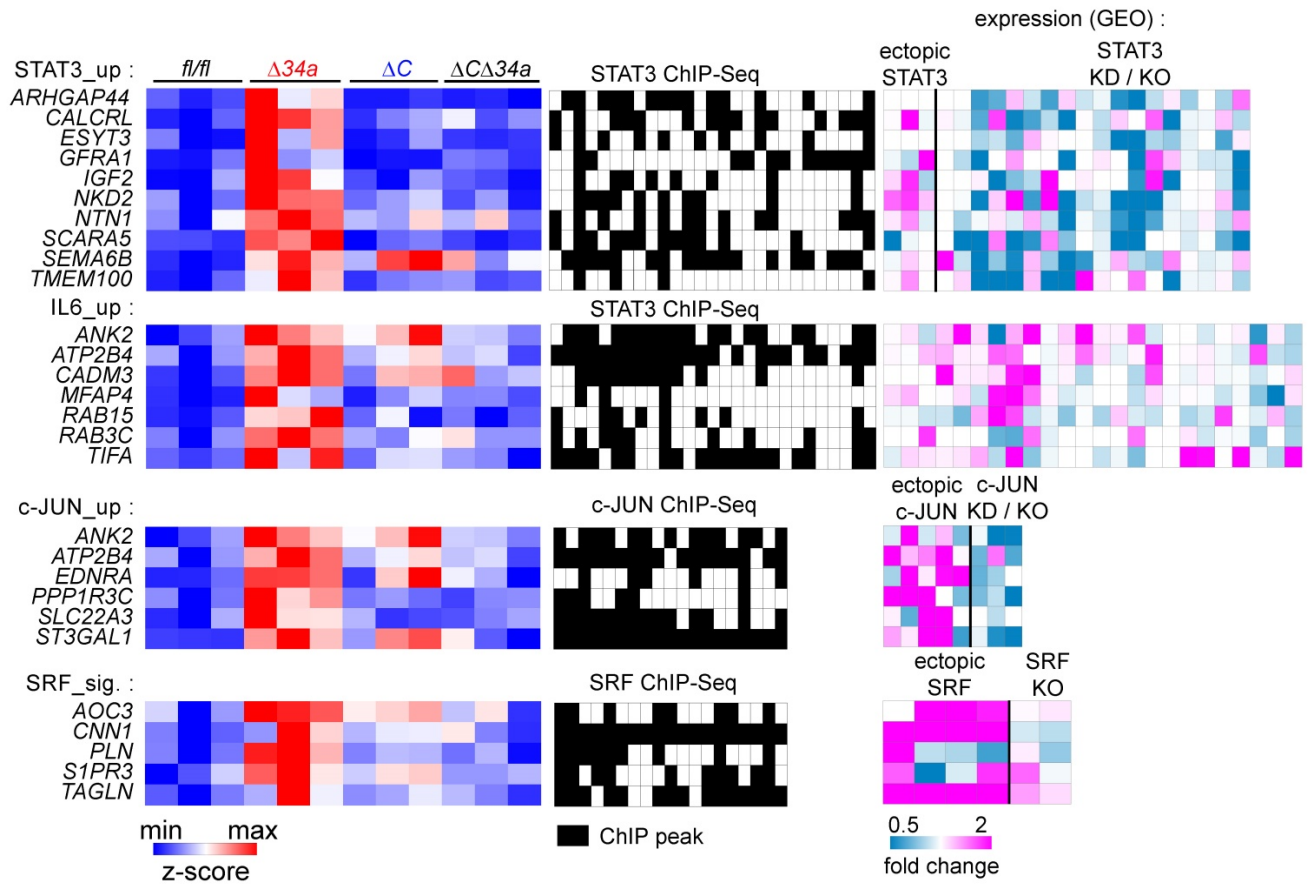
** Characterization of coordinated regulation of Mir34a target mRNA expression by Mir34a and the transcription factors STAT3, AP1 (JUN:FOS) and SRF.** Left: Heat-maps showing the expression of indicated genes in adenomas from *Mir34a^fl/fl^;Csf1r^fl/fl^;Apc*^Min/+^, *Mir34a^ΔIEC^;Apc*^Min/+^, *Csf1r^ΔIEC^;Apc*^Min/+^, or *Mir34a^ΔIEC^;Csf1r^ΔIEC^;Apc*^Min/+^ mice. Middle: Heatmaps showing promoter occupancy by STAT3, c-JUN, or SRF according to GEO ChIP-seq datasets. Right: Heat-maps showing the fold change in expression of the indicated mRNAs in GEO datasets after STAT3 ectopic expression or knockdown (KD)/knockout (KO), IL6 treatment, and c-JUN or SRF ectopic expression or knockdown (KD)/knockout (KO). GEO data are shown from left to right in the order of the underlying datasets listed in Supplementary Table S6-9.

In order to further characterize how concomitant deletion of *Csf1r* affects transcriptome changes induced by loss of *Mir34a* in adenomas, we determined which Mir34a targets may be coordinately regulated by both Mir34a and STAT3, AP1 (JUN:FOS) and SRF (Fig. 8) in a coherent feed-forward manner (hereafter referred to as “Mir34a/TF targets”). Thereby, we identified 26 predicted Mir34a targets that are presumably directly regulated by either STAT3, JUN or SRF, as evidenced by analysis of previously published ChIP-Seq and RNA expression datasets (Fig. 8). Of note, only two (*Ank2, Gfra1*) of the identified targets have been characterized as direct miR-34a targets previously 60, 61. Next, two of these targets, *Ntn1/Netrin* and *Tagln/Transgelin* were selected for further analysis (Supplementary Fig. S3 and Supplementary Fig. S4): Ntn1 is known to mediate survival signals that contribute to tumorigenesis 62-64. Tagln may exert oncogenic functions by regulation of multiple tumor-relevant processes, such as EMT, invasion and metastasis 65, 66. The 3'-UTR of murine *Ntn1* contains three Mir34a SMSs (Supplementary Fig. S3A). Ectopic *pre-Mir34a* significantly repressed a murine *Ntn1* 3'-UTR reporters and mutations of the 3 SMS abrogated their repression by ectopic Mir34a (Supplementary Fig. S3B). In addition, *Ntn1* mRNA and protein expression was significantly repressed by ectopic Mir34a in the murine CRC cell line CT26 (Supplementary Fig. S3C). Similar results were obtained for *Tagln* (Supplementary Fig. S3D-F). Interestingly, the 3'-UTR of the human *NTN1* and *TAGLN* mRNA also harbors a miR-34a seed-matching site (Supplementary Fig. S4A and D). Human *NTN1* and *TAGLN* 3'-UTR-reporters were significantly repressed after co-transfection of *pre-miR-34a* in an SMS-dependent manner (Supplementary Fig. S4B and E). Furthermore, ectopic expression of *pri-miR-34a* significantly decreased *NTN1* and *TAGLN* mRNA and protein expression levels in the human CRC cell line SW480 (Supplementary Fig. S4C and F). Taken together, these results show that *Ntn1* and *Tagln* are conserved and direct targets of miR-34a.

### Clinical associations of *Mir34a*-related expression signatures

**Figure 9:**
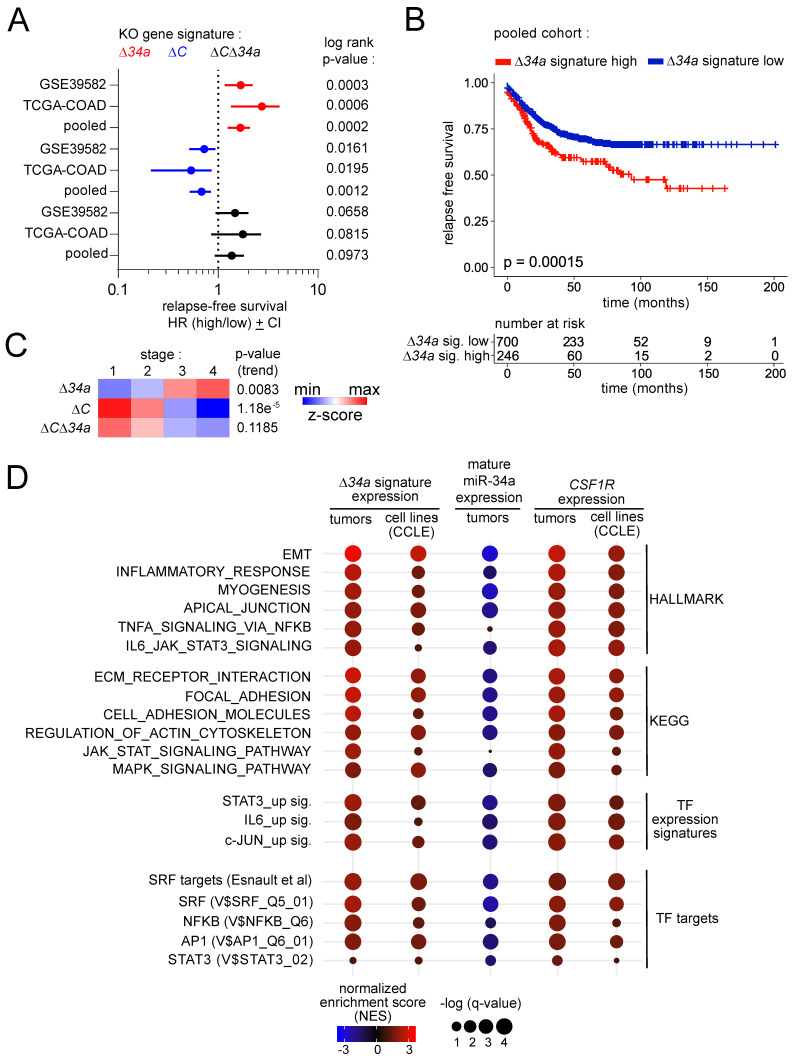
** Clinical associations of *Mir34a*-related expression signatures. A** Forest plots showing hazard ratios for relapse free survival by comparing patients with high versus low expression of the *Δ34a, ΔC* and* Δ34aΔC* expression signatures in the TCGA and GSE39582 patient cohorts, and the pooled cohort comprising both individual cohorts. Dots represent Hazard ratios and horizontal lines show 95% CI. P-values were calculated using the log-rank method. **B** Kaplan-Meier analysis of relapse free survival for patients with high or low expression of the *Δ34a* expression signature using the pooled dataset of the TCGA and GSE39582 patient cohorts (n = 946 patients). The significance was calculated with the log-rank test and the x-axis represents relapse free survival in months. Below the graph the numbers of patients at risk with high or low expression of the *Δ34a* expression signature at the respective time point is provided. **C** Heat-map showing the expression of the *Δ34a, ΔC* and* Δ34aΔC* expression signatures in the indicated tumor stages using the pooled dataset of the TCGA and GSE39582 patient cohorts. The p-values for linear trend in expression from stage 1 to stage 4 are provided. **D** Gene Set Enrichment Analysis (GSEA) of the indicated functional categories showing their association with the *Δ34a* signature, mature *miR-34a* expression and *Csf1r* expression in tumors using the pooled dataset of the TCGA and GSE39582 patient cohorts (tumors) and cancer cell lines (CCLE). The significance of enrichments is presented by normalized enrichment scores (NES) and false discovery rate-adjusted q values.

Next, we determined whether the expression signatures we identified in *Mir34a*^ΔIEC^, *Csf1r^ΔIEC^* and *Mir34a*^ΔIEC^*;Csf1r^ΔIEC^* adenomas are associated with clinical parameters, such as patient survival and tumor stage, in two independent CRC patient cohorts (TCGA-COAD and GSE39582). Interestingly, in primary CRCs the *Mir34a*^ΔIEC^ signature was associated with poor relapse free survival in both patient cohorts (Fig. 9A). Moreover, a pooled patient cohort comprising 946 patients from both patient cohorts recapitulated these findings with increased statistical significance (Fig. 9 B). Conversely, the *Csf1r^ΔIEC^* signature was associated with improved relapse free survival (Fig. 9A). The *Mir34a*^ΔIEC^*;Csf1r^ΔIEC^* expression signature was not associated with a significant difference in relapse free survival (Fig. 9A). Moreover, the *Mir34a*^ΔIEC^ signature was elevated in advanced tumor stages, whereas the *Csf1r^ΔIEC^* and* Mir34a*^ΔIEC^*;Csf1r^ΔIEC^* expression signatures were elevated in less advanced tumor stages (Fig. 9C).

Next, we analyzed whether the *Mir34a*^ΔIEC^ adenoma signature, mature miR-34a expression and *CSF1R* expression is associated with HALLMARK, KEGG, as well as TF expression and TF target signatures in human CR tumors or CRC cell lines (Fig. 9D). In human CRCs, the* Mir34a*^ΔIEC^ adenoma gene signature was associated with EMT, inflammation and actin cytoskeleton signatures, as well as with the TNFα/NFKB, IL6/STAT3 and MAPK signaling pathways. Moreover, it was associated with STAT3 and JUN expression signatures. Remarkably, several SRF, AP1 and NFKB target signatures were strongly associated with the *Mir34a*^ΔIEC^ adenoma signature. Moreover, these associations could also be found in CRC cell lines, which strongly suggests tumor cell intrinsic regulations. Conversely, expression of mature miR-34a displayed a strong negative correlation with the large majority of the analyzed gene signatures. Furthermore, *CSF1R* expression positively correlated with the majority of the analyzed gene signatures, which largely reflected the associations found for the *Mir34a*^ΔIEC^ adenoma signature. These findings indicate that loss of *miR-34a*, and the resulting elevated expression of CSF1R in human CRCs is also associated with the signaling pathways and TF expression signatures identified in *Mir34a*-deficient, murine adenomas in this study.

**Figure 10:**
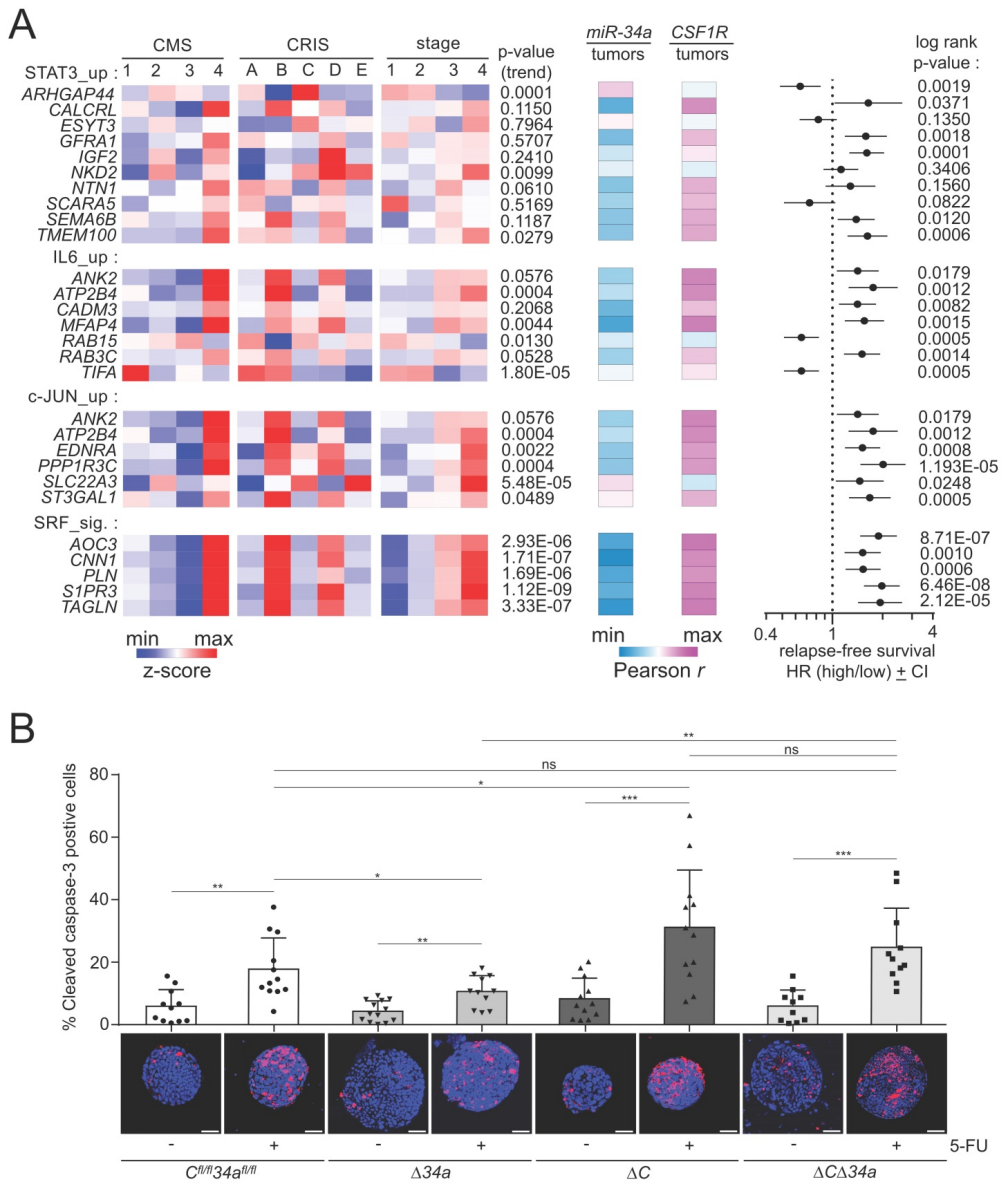
** Associations of miR-34a target mRNA expression with clinico-pathological parameters and effects of *Mir34a/Csf1r* loss on the response to 5-FU. A** Left: Heat-maps showing the expression of indicated mRNAs in CMS and CRIS molecular subtypes and tumor stages. The p-values for linear trend in expression from stage 1 to stage 4 are indicated. Middle: Heat-maps showing the expression correlation between the indicated mRNAs and mature *miR-34a* and *CSF1R*. Right: Forest plot showing hazard ratios for relapse free survival by comparing patients with high versus low expression of the indicated mRNAs. Dots represent hazard ratios and horizontal lines show 95% CI. P-values were calculated using the log-rank method. **B** Tumoroids were cultured for 3 days and then treated with or without 5-FU (25 µg/ml) for 48 hours. ≥ 10 tumoroids from 3 mice per group are analyzed. Scale bar: 50 μm. Results are presented as mean ± SD using a two-tailed unpaired Student's t test. *P < 0.05, **P < 0.01, ***P <0.001, or ****P < 0.0001.

Next, we analyzed whether elevated expression of the “Mir34a/TF” targets (Fig. 8) is associated with molecular subtypes and stages of CRC, or CRC patient survival (Fig. 10A). Remarkably, the majority of the Mir34a/TF targets showed elevated expression in CMS4 tumors. Moreover, numerous targets showed elevated expression in CRIS-B and -D subtypes. The CMS4, CRISB and CRISD molecular subtypes display mesenchymal and WNT-associated expression signatures, respectively 47, 54. Furthermore, the majority of Mir34a targets displayed elevated expression in the advanced tumor stages 3 and 4. While this pattern was less evident for STAT3 target genes, it was found for the majority of direct JUN and SRF targets. Strikingly, the large majority of the Mir34a/TF targets displayed a negative correlation with mature miR-34a expression, as well as a positive correlation with *CSF1R* expression in human CRCs (Fig. 10A), suggesting that the proposed feed-forward regulation of these genes is conserved between murine and human cells. Moreover, elevated expression of the majority of “Mir34a/TF” target mRNAs was significantly associated with poor relapse free survival of CRC patients (Fig. 10A).

### *Mir34a* and *Csf1r* influence therapeutic responses in tumoroids

We have previously reported a role of the miR-34/CSF1R/STAT3 axis in the response to 5-Fluorouracil (5-FU) in human CRC cell lines *ex vivo* 21. Therefore, we asked whether the genetic inactivation of *Mir34a* and/or *Csf1r* would modulate the response to 5-FU in tumoroids (Fig. 10B). Interestingly, we found that *Mir34a*-deficient tumoroids showed less apoptosis in response to 5-FU, while *Csf1r*-deficient tumoroids displayed more apoptosis in response to 5-FU. When both genes were deleted the response to 5-FU was not significantly different from tumoroids with intact *Mir34a* and *Csf1r* genes (Fig. 10B). Since the effects of deleting *Mir34a* and *Csf1r* abrogated the effect of single gene deletions, these results showed that *Csf1r* is an important mediator of the 5-FU-resistance caused by *Mir34a* inactivation.

## Discussion

Here, we determined a tumor suppressive role for *Mir34a*, whereas *Csf1r* displayed a tumor-promoting function in intestinal epithelial cells. The combined deletion of *Mir34a* and *Csf1r* demonstrated that activation of Csf1r is required for the effects of *Mir34a* loss during intestinal tumorigenesis. Therefore, the up-regulation of Csf1r expression that results from the inactivation of *Mir34a* is an important mediator of the pro-tumorigenic effects of *Mir34a* inactivation in mice and presumably also in human CRC. We had previously identified *CSF1R* as a direct target of miR-34a in human CRC cells, and characterized a CSF1R-STAT3-miR-34a feedback regulation 21. Here we confirmed that *Csf1r* also represents a direct target of *Mir34a* in mice. Furthermore, we provide genetic evidence that this regulation occurs *in vivo*, since *Mir-34a* and *Csf1r* displayed reciprocal repression in murine intestinal epithelium and derived adenomas.

In this study, we show that the absence of *miR-34a* in IECs results in changes of the cellular composition of the small intestine, i.e. an increase of Paneth cells and *Olfm4-*positive stem cells, and an increased tumor burden as well as decreased survival in *Apc*^Min/+^ mice. In addition, we detected an increase in entero-endocrine cells in *Mir34a*^ΔIEC^*;Apc*^Min/+^ mice. Entero-endocrine cells control microbial and intestinal homeostasis via innate immune signaling 67.

So far, the role of *Csf1r* has not been studied in mouse models of intestinal tumorigenesis. In this study, *Apc*^Min/+^ mice with IEC-specific deletion of *Csf1r* via *Villin-*Cre were established and analyzed. Notably, less and smaller adenomas as well as a prolonged survival time were observed in *Csf1r^ΔIEC^;Apc*^Min/+^ mice*.* Besides the modulation of the SI architecture and changes in the number of secretory cells, a decrease in the frequency of intestinal stem cells as well as a decrease in the *Lgr5*-positive tumor area was detected in *Apc*^Min/+^ mice with IECs-specific deletion of *Csf1r*. Furthermore, the number and mean size of tumoroids that could be derived from *Csf1r*-deficient adenomas was decreased. Our results imply that *Csf1r* critically contributes to tumor formation caused by loss of *Apc* in intestinal epithelial cells.

Here, we observed that the phenotypes of* Apc*^Min/+^ mice with a combined deletion of *Csf1r and Mir34a* were similar as that of *Apc*^Min/+^ mice, indicating that the effects of *Mir34a* deletion on intestinal tumorigenesis are mediated, at least in part, by the up-regulation of *Csf1r* expression. It should however be mentioned that the deletion of *Csf1r* studied here could have a dominant effect in the context of *Mir34a* inactivation which is not identical to reverting the elevation of *Csf1r* expression caused by *Mir34a* loss. To formally proof that *Mir34a* suppresses tumorigenesis by down-regulation of *Csf1r* expression a deletion of the SMS for *Mir34a* in the 3'-UTR of *Csf1r* should be generated and studied similarly in the future.

Apart from the opposing effects of *Mir34a* and *Csf1r* on proliferation, apoptosis and STAT3 signaling, their antagonistic effects on the tumor-environment and intestinal cancer stem cells might also be responsible for the compensatory effect of deleting both genes. Here, *Mir34a* inactivation resulted in a *Csf1r*-dependent increase in tumor-associated fibroblasts, macrophages, neutrophils, T- and B-cells. Therefore, the up-regulation of *Csf1r* presumably mediated the effects of *Mir34a* loss on the tumor microenvironment.

In this study, we showed that *Csf1r* is an important mediator of the effects of *Mir34a* loss on stemness and Wnt signaling. Intestinal stem cells are thought to represent the tumor initiating cells during intestinal tumorigenesis 68, and multiple signaling pathways, including Wnt/β-catenin pathways, regulate the cell-cell and cell-matrix interactions in the intestinal stem cell niche 69, 70. Interestingly, *Lgr5,* which was up-regulated in* Mir34a*-deficient adenomas and down-regulated in *Csf1r*-deficient adenomas, is not only a stem cell marker, but also potentiates Wnt/β-catenin signaling 71. In addition, our expression analyses showed that, among potential Mir34a targets, genes associated with the induction of STAT3, JUN and SRF expression signatures are presumably involved in the opposing regulation of intestinal homeostasis and tumor formation by Mir34a and Csf1r signaling. In this study, we identified and characterized *Ntn1/Netrin-1* and *Transgelin/Tagln* as direct targets of miR-34a in both murine and human cells. *Ntn1/Netrin-1* and *Transgelin/Tagln* are coordinately regulated by both, miR-34a and the CSF1R-induced JAK-STAT, MAPK and Rho-actin signaling pathways. In addition, we also showed *Mir34a*-deletion-associated expression signatures correlate with clinico-pathological features of primary CRC and poor patient survival. Furthermore, inactivation of *Mir34a* and/or *Csf1r* modulated the cellular response to 5-FU in tumoroids, suggesting a role the miR-34a/CSF1R axis in resistance to 5-FU and warranting further studies on their potential role in chemo-resistance.

Netrin-1 exerts its functions through interaction with its receptors, including deleted in colorectal cancer (DCC) 72. Notably, DCC, is localized on chromosome 18q, where frequent deletions are observed in CRC 73, and is down-regulated in more than half of CRCs 74. Contrary to the effect of Netrin-1, DCC acts as an inhibitor of cell invasion, tumor growth and metastasis 75, and limits the progression of intestinal tumors in mouse models 76. DCC regulates apoptosis as a dependency receptor 77: it suppresses cell apoptosis when engaged by Netrin-1, while it triggers apoptosis in the absence of Netrin-1. Therefore, DCC represents a conditional tumor suppressor. Furthermore, Netrin-1 regulates cancer cell motility and tumorigenesis via multiple pathways, including YAP signaling 78, ERK/MAPK signaling 79, 80 and Notch signaling 81. Therefore, the down-regulation of Netrin-1 by miR-34a may inhibit multiple pro-tumorigenic pathways in CRC.

Transgelin, belongs to a family of actin-binding proteins (ABPs) and may represent a promising target for treating multiple cancers 66, 82, including CRC 83. High expression of transgelin is associated with tumor progression and poor prognosis for CRC patients 65, 84. Transgelin is involved in remodeling of the actin cytoskeleton and promoting cell motility 85. Enhanced expression of transgelin promotes tumor cell proliferation, migration, growth and EMT 65, 86, 87. In addition, transgelin promotes the progression and metastasis of CRC through a series of complex signaling pathways, including Rho 88, AKT and JNK 89, and TGFβ signaling pathways 65, 90. Repression of transgelin may therefore mediate tumor suppressive effects of miR-34a in CRC.

In conclusion, our results show that deregulation of the Mir34a/Csf1r double-negative feedback loop plays an important role during intestinal tumorigenesis. Our findings suggest that it may be beneficial to suppress CSF1R activity to obtain a therapeutic effect in CRCs that display inactivation of *miR-34a* and therefore activation of the CSF1R pathway. Notably, 75% of CRCs were shown to display epigenetic silencing of *miR-34a* 12. Furthermore, restauration of miR-34a function by mimics and concomitant inhibition of CSF1R by small drugs may represent a feasible approach to treat CRC in the future.

## Supplementary Material

Supplementary figures and tables.
