# Supplementary material for: Csf1r mediates enhancement of intestinal tumorigenesis caused by inactivation of Mir34a
Source: Int J Biol Sci. 2022 Aug 29;18(14):5415–37. doi: 10.7150/ijbs.75503 (PMC9461672; doi:10.7150/ijbs.75503)
Supplement: Supplementary file 1 — Supplementary figures and tables. [file ijbsv18p5415s1.pdf]

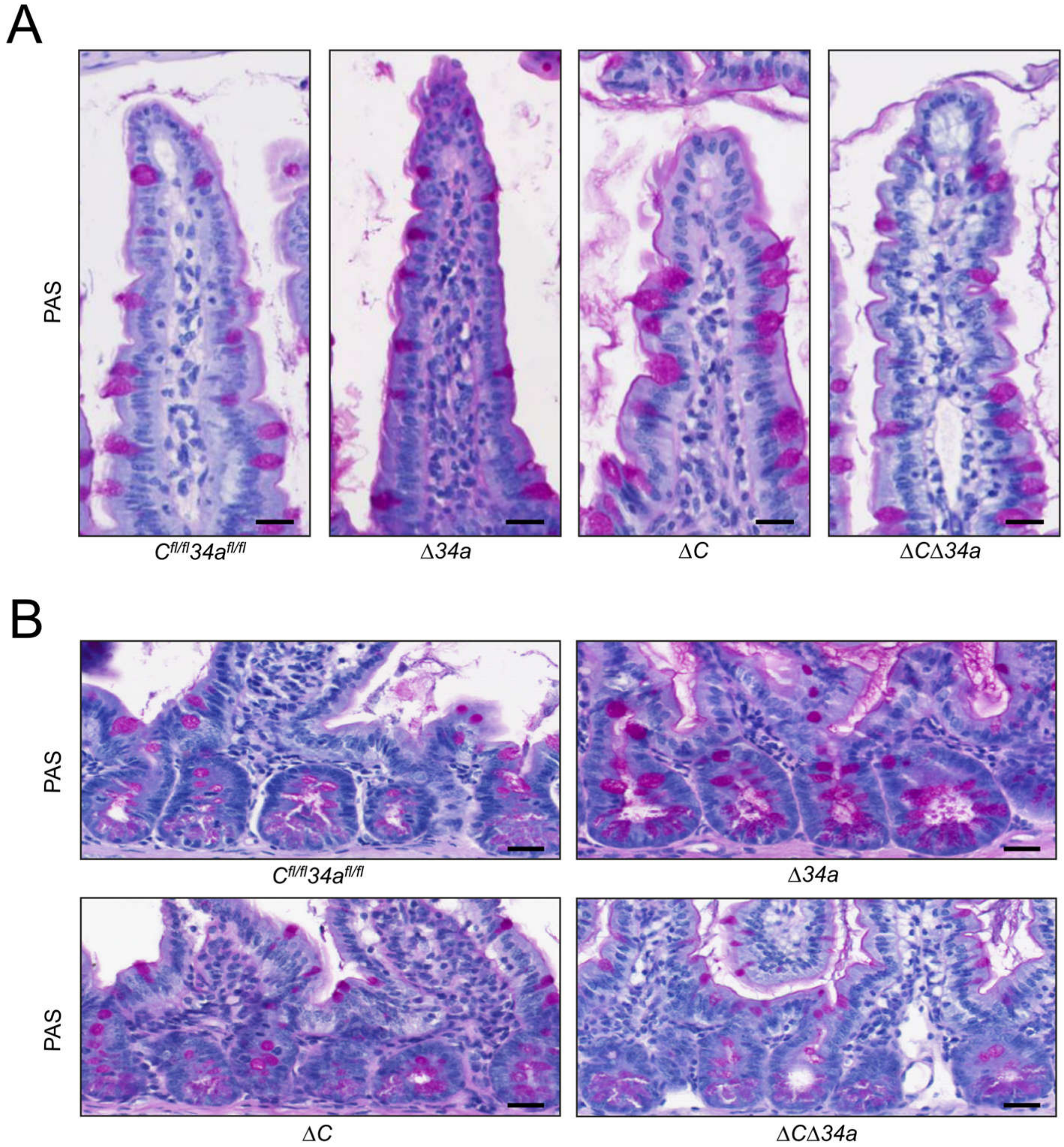

Figure S1. Effect of *Mir34a* and/or *Csf1r* inactivation on goblet and Paneth cells in the 18 weeks old *Apc*$^{\mathrm{Min/+}}$ mice.

A, B Detection of goblet cells on normal villus (**A**) and Paneth cells at normal crypt (**B**) from *Apc*$^{\mathrm{Min/+}}$ mice with the indicated genotype by Periodic acid-Schiff (PAS) staining. Scale bar: 20 µm.

Figure S1

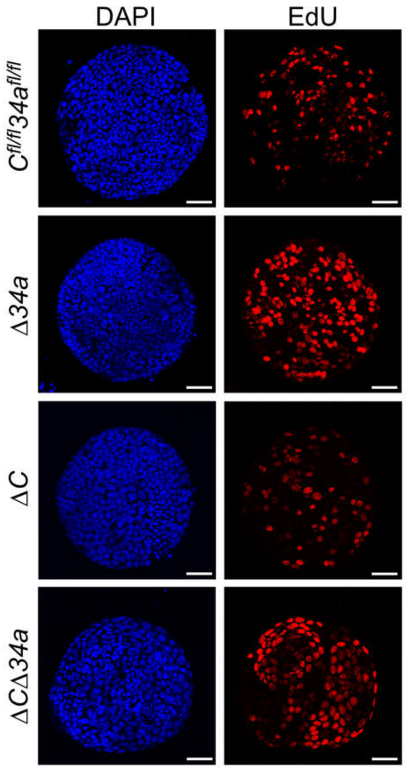

Figure S2, related to Figure 5F

Representative images of DAPI and EdU labeling tumoroids derived from adenomas with the indicated genotypes. Scale bar: 40 μm.

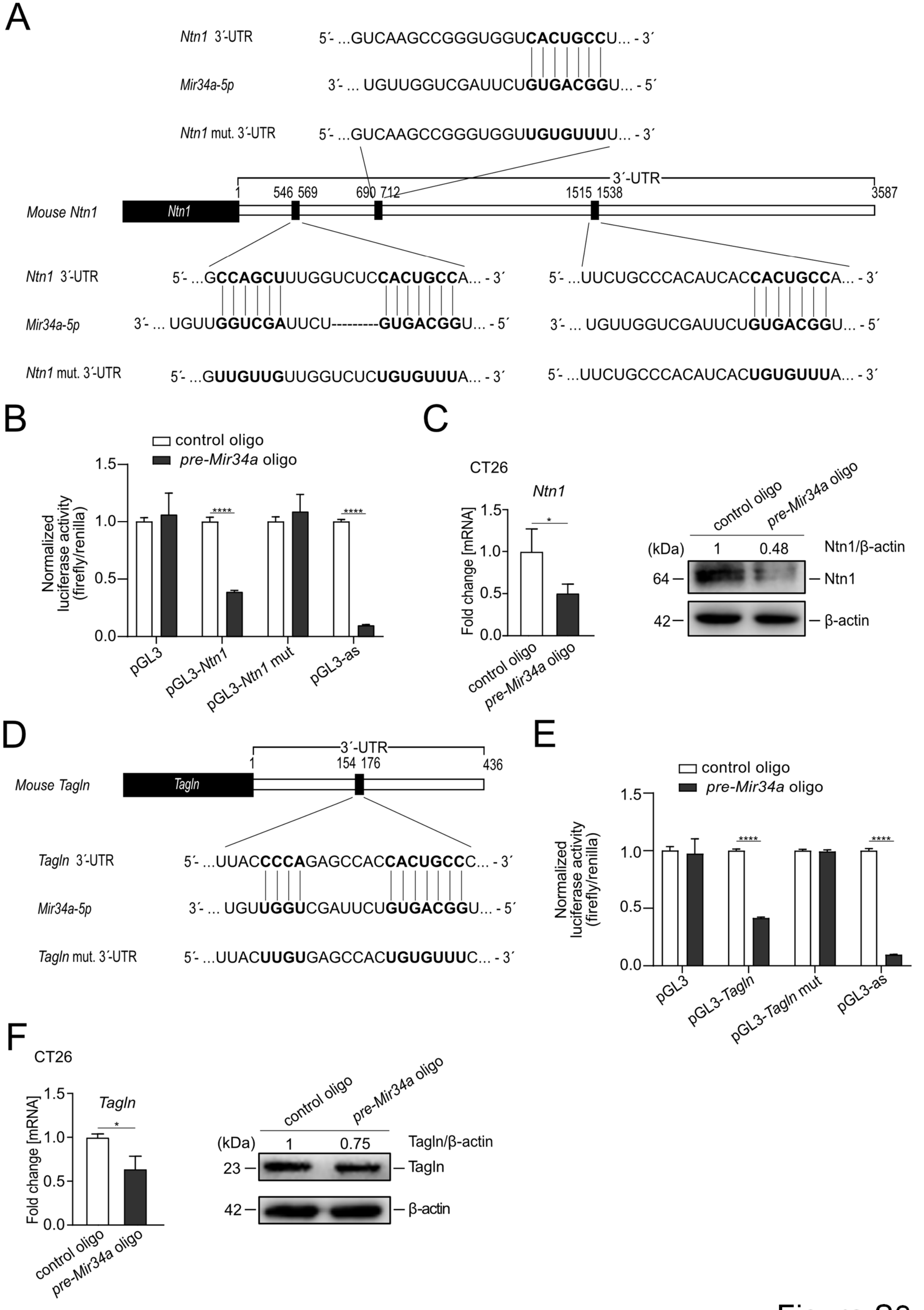
A
Ntn1 3´-UTR 5´- ...GUCAAGCCGGGUGGUCACUGCCU... - 3´
Mir34a-5p 3´- ... UGUUGGUCGAUUCUGUGACGGU... - 5´
Ntn1 mut. 3´-UTR 5´- ...GUCAAGCCGGGUGGUUGUGUUUU... - 3´
3´-UTR
1 546 569 690 712 1515 1538 3587
Mouse Ntn1
Ntn1
Ntn1 3´-UTR 5´- ...GCCAGCUUUGGUCUCCACUGCCA... - 3´
Mir34a-5p 3´- ... UGUUGGUCGAUUCU---------GUGACGGU... - 5´
Ntn1 mut. 3´-UTR 5´- ...GUUGUUGUUGGUCUCUGUGUUUA... - 3´
5´- ...UUCUGCCCACAUCACCACUGCCA... - 3´
3´- ... UGUUGGUCGAUUCUGUGACGGU... - 5´
5´- ...UUCUGCCCACAUCACUGUGUUUA... - 3´
B
control oligo
pre-Mir34a oligo
Normalized luciferase activity (firefly/renilla)
1.5 1.0 0.5 0.0
****
****
pGL3
pGL3-Ntn1
pGL3-Ntn1 mut
pGL3-as
C
CT26
Ntn1
Fold change [mRNA]
1.5 1.0 0.5 0.0
*
control oligo
pre-Mir34a oligo
(kDa) 1 0.48 Ntn1/β-actin
64 — Ntn1
42 — β-actin
D
3´-UTR
1 154 176 436
Mouse Tagln
Tagln
Tagln 3´-UTR 5´- ...UUACCCCAGAGCCACCACUGCCC... - 3´
Mir34a-5p 3´- ... UGUUGGUCGAUUCUGUGACGGU... - 5´
Tagln mut. 3´-UTR 5´- ...UUACUUGUGAGCCACUGUGUUUC... - 3´
E
control oligo
pre-Mir34a oligo
Normalized luciferase activity (firefly/renilla)
1.5 1.0 0.5 0.0
****
****
pGL3
pGL3-Tagln
pGL3-Tagln mut
pGL3-as
F
CT26
Tagln
Fold change [mRNA]
1.5 1.0 0.5 0.0
*
control oligo
pre-Mir34a oligo
(kDa) 1 0.75 Tagln/β-actin
23 — Tagln
42 — β-actin

Figure S3

Figure S3. Characterization of *Ntn1* and *Tagln* as direct targets of *Mir34a*.

A Scheme of the Mir34a seed, the seed-matching sequences and its targeted mutation in the 3'-UTR of mouse *Ntn1* mRNA.

B Dual-reporter assay after transfection of H1299 cells with the indicated *pre-Mir34a* oligonucleotides using the murine *Ntn1* 3'-UTR reporter.

C qPCR (left panel) and Western blot analysis (right panel) of *Ntn1* in CT26 cells after addition of *pre-Mir34a* oligonucleotides.

D Scheme of the Mir34a seed, the seed-matching sequences and its targeted mutation in the 3'-UTR of mouse *Tagln* mRNA.

E Dual-reporter assay after transfection of H1299 cells with the indicated *pre-Mir34a* oligonucleotides using the murine *Tagln* 3'-UTR reporter.

F qPCR (left panel) and Western blot analysis (right panel) of *Tagln* in CT26 cells after addition of *pre-Mir34a* oligonucleotides.

Data information: In (**B, C, E, F**), results are presented as mean ± SD (n=3) using the two-tailed unpaired Student's t-test. **P* < 0.05, ***P* < 0.01, ****P* <0.001, or *****P* < 0.0001.

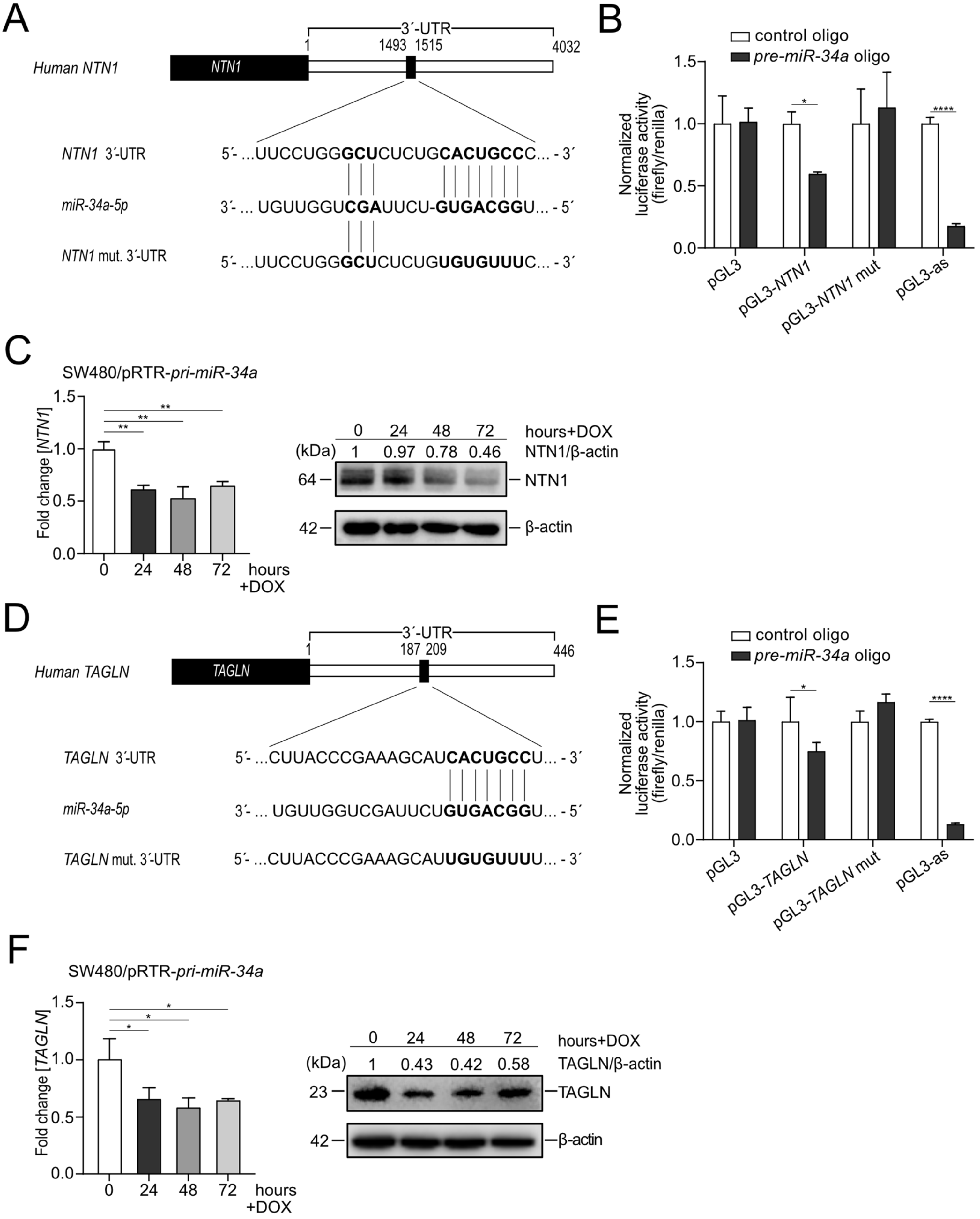
A
3´-UTR
1
1493 1515
4032
Human NTN1
NTN1
NTN1 3´-UTR
5´- ...UUCCUGGGCUCUCUGCACUGCCC... - 3´
miR-34a-5p
3´- ... UGUUGGUCGAUUCU-GUGACGGU... - 5´
NTN1 mut. 3´-UTR
5´- ...UUCCUGGGCUCUCUGUGUGUUUC... - 3´
B
control oligo
pre-miR-34a oligo
Normalized luciferase activity (firefly/renilla)
pGL3
pGL3-NTN1
pGL3-NTN1 mut
pGL3-as
C
SW480/pRTR-pri-miR-34a
Fold change [NTN1]
hours +DOX
0
24
48
72
hours+DOX
(kDa)
1
0.97
0.78
0.46
NTN1/β-actin
64
NTN1
42
β-actin
D
3´-UTR
1
187 209
446
Human TAGLN
TAGLN
TAGLN 3´-UTR
5´- ...CUUACCCGAAAGCAUCACUGCCU... - 3´
miR-34a-5p
3´- ... UGUUGGUCGAUUCUGUGACGGU... - 5´
TAGLN mut. 3´-UTR
5´- ...CUUACCCGAAAGCAUUGUGUUUU... - 3´
E
control oligo
pre-miR-34a oligo
Normalized luciferase activity (firefly/renilla)
pGL3
pGL3-TAGLN
pGL3-TAGLN mut
pGL3-as
F
SW480/pRTR-pri-miR-34a
Fold change [TAGLN]
hours +DOX
0
24
48
72
hours+DOX
(kDa)
1
0.43
0.42
0.58
TAGLN/β-actin
23
TAGLN
42
β-actin

Figure S4

Figure S4. *NTN1* and *TAGLN* are the direct targets of miR-34a in human.

A Scheme of the miR-34a seed, the seed-matching sequences and its targeted mutation in the 3'-UTR of human *NTN1* mRNA.

B Dual-reporter assay after transfection of H1299 cells with the indicated *pre-miR-34a* oligonucleotides using the human *NTN1* 3'-UTR reporter.

C qPCR (left panel) and Western blot analysis (right panel) of *NTN1* in SW480/pRTR-*pri-miR-34a* cells after addition of DOX.

D Scheme of the miR-34a seed, the seed-matching sequences and its targeted mutation in the 3'-UTR of human *TAGLN* mRNA.

E Dual-reporter assay after transfection of H1299 cells with the indicated *pre-miR-34a* oligonucleotides using the human *TAGLN* 3'-UTR reporter.

F qPCR (left panel) and Western blot analysis (right panel) of *TAGLN* in SW480/pRTR-*pri-miR-34a* cells after addition of DOX.

Data information: In (**B, C, E, F**), results are presented as mean ± SD (n=3) using the two-tailed unpaired Student's t-test. *$P < 0.05$, **$P < 0.01$, ***$P < 0.001$, or ****$P < 0.0001$.

Supplementary Tables

Table of contents:

Table S1. Oligonucleotides used for mouse genotyping.

Table S2. List of antibodies and reagents.

Table S3. List of the oligonucleotides used for fluorescence in situ hybridization (FISH).

Table S4. List of the oligonucleotides and the primers used for cloning and mutagenesis.

Table S5. List of primers used for qPCR.

Table S6. STAT3-related public datasets used to identify potential STAT3 target genes.

Table S7. IL6/STAT3-related public datasets used to identify potential STAT3 target genes.

Table S8. c-JUN-related public datasets used to identify potential c-JUN target genes.

Table S9. SRF-related public datasets used to identify potential SRF target genes.

Table S10, related to Figure 6B (upper panel).

List of the significantly up-regulated and down-regulated mRNAs in *Mir34a*-deficient adenomas compared to *Apc*$^{Min/+}$ adenomas.

Table S11, related to Figure 6B (middle panel).

List of the significantly up-regulated and down-regulated mRNAs in *Csf1r*-deficient adenomas compared to *Apc*$^{Min/+}$ adenomas.

Table S12, related to Figure 6B (lower panel).

List of the significantly up-regulated and down-regulated mRNAs in *Csf1r/Mir34a*-deficient adenomas compared to *Apc*$^{Min/+}$ adenomas.

Table S13, related to Figure 6D (upper panel).

List of the significantly up-regulated and down-regulated mRNAs in *Mir34a*-deficient tumoroids compared to *Apc*$^{Min/+}$ tumoroids.

Table S14, related to Figure 6D (middle panel).

List of the significantly up-regulated and down-regulated mRNAs in *Csf1r*-deficient tumoroids compared to *Apc*$^{Min/+}$ tumoroids.

Table S15, related to Figure 6D (lower panel).

List of the significantly up-regulated and down-regulated mRNAs in *Csf1r/Mir34a*-deficient tumoroids compared to *Apc*$^{Min/+}$ tumoroids.

Table S1, related to Materials and Methods.

Oligonucleotides used for mouse genotyping.

**Name**	**Sequence (5'-3')**
Csf1r For	CATGGCTGTGGCCTAGAGA
Csf1r Rev	GGACTAGCCACCATGTCTCC
miR-34a For	ACCTTGCAGGTGCTCAGAAT
miR-34a Rev-a	TGGAGCTAACGGAGTGTGTG
miR-34a Rev-b	CTACCCAAGCTCGACGAAGT
miR-34a Rev-c	TGCAGCACTTCTAGGGCAGT
Vil-Cre For	CGCGAACATCTTCAGGTTCT
Vil-Cre Rev	CAAGCCTGGCTCGACGGCC
Apc^{Min} wt	GCCATCCCTTCACGTTAG
Apc^{Min} com	TTCCACTTTGGCATAAGGC
Apc^{Min} mut	TTCTGAGAAAGACAGAAGTTA

Table S2, related to Materials and Methods.

List of antibodies and reagents.

**Name**	**Species**	**Catalog No.**	**Company**	**Use**	**Dilution**	**Source**
CSF1R	Mouse	# SAB4500500	Sigma-Aldrich	IHC	1:100	Rabbit
MUC2	Mouse	E-AB-70212	Elabscience	IHC	1:2000	Rabbit
Lysozyme	Mouse	ab108508	Abcam	IHC	1:1000	Rabbit
Chromogranin A	Mouse	E-AB-40339	Elabscience	IHC	1:800	Rabbit
Ki-67	Mouse	#12202	Cell Signaling	IHC	1:400	Rabbit
Cleaved-Caspase-3	Mouse	#9664	CST	IHC, IF	1:500, 1:100	Rabbit
p-STAT3	Mouse	#9145	CST	IHC	1:200	Rabbit
Vimentin	Mouse	ab92547	Abcam	IHC	1:500	Rabbit
CD3	Mouse	A 0452	DAKO	IHC	1:100	Rabbit
CD45R	Mouse	550286	BD	IHC	1:100	Rat
CD68	Mouse	E-AB-70389	Elabscience	IHC	1:300	Rabbit
Ly6G	Mouse	E-AB-70094	Elabscience	IHC	1:400	Rabbit
β-catenin	Mouse	ab32572	Abcam	IHC	1:500	Rabbit
ImmPRESS REAGENT Anti-Rabbit IgG	Rabbit	MP-7401	Vector	IHC	Ready-to use	Horse
ImmPRESS REAGENT Anti-Rat IgG	Rat	MP-7444	Vector	IHC	Ready-to use	Goat
DAB Substrate Kit		SK-4100	Vector	IHC		

AEC Substrate Kit		ab64252	Abcam	IHC		
Anti-Rabbit-Cy3	Rabbit	711-165-152	Jackson Immuno-Research	IF	1:100	donkey
NTN1	Human/ mouse	bs-1858R	Bioss Antibodies	WB	1:1000	Rabbit
TAGLN	Human/ mouse	PA5-29767	Invitrogen	WB	1:1000	Rabbit
β-actin	Human/ mouse	# A2066	Sigma-Aldrich	WB	1:1000	Rabbit
Anti-rabbit HRP	Rabbit	# A0545	Sigma	WB	1:10000	Goat

Table S3, related to Materials and Methods.

List of the oligonucleotides used for fluorescence in situ hybridization (FISH).

**Name**	**Sequence (5’-3’)**	**Company**
universal eubacteria probe (EUB338)	[FITC]-5’-GCTGCCTCCCGTAGGAGT-3’	Metabion
negative control probe (NON338)	[Cy3]-5’-CGACGGAGGGCATCCTCA-3’	Metabion

Table S4, related to Materials and Methods.

List of the oligonucleotides and the primers used for cloning and mutagenesis.

**Name**	**Sequence (5’-3’)**	**Company**
Murine *Csf1r* 3’-UTR For	ATTACCGGTACATATGGACTTCGC CCTCA	Metabion
Murine *Csf1r* 3’-UTR Rev	ATTCTGCAGGGTGTTTGT TGGTGTGGTCA	Metabion
Murine *Csf1r* 3’-UTR mutant For	CCCAGAGCCTGGGCCATCAGTCG GAGTGGGGTTCTCACAGT	Metabion
Murine *Csf1r* 3’-UTR mutant Rev	ACTGTGAGAACCCCACTCCGACTG ATGGCCCAGGCTCTGGG	Metabion
Murine *Ntn1* 3’-UTR For	ATTACCGGTTCTCCATCACCCGCT GTCTAGG	Metabion
Murine *Ntn1* 3’-UTR Rev	ATTCTGCAGAGAGTGAATCCCTGC CTCGCAG	Metabion
Murine *Ntn1* 3’-UTR mutant For-1	GTTGTTGGTCTCTGTGTTTACCTGC TGGGCTGGTCTCC	Metabion

Murine *Ntn1* 3'-UTR mutant Rev-1	ACACAGAGACCAACAACAACTGAT CCCAGTGTCATCGGG	Metabion
Murine *Ntn1* 3'-UTR mutant For-2	GTGGTTGTGTTTTCTGCTGGAGCT GCCTGTG	Metabion
Murine *Ntn1* 3'-UTR mutant Rev-2	GCAGAAAACACAACCACCCGGCTT GACTTCA	Metabion
Murine *Ntn1* 3'-UTR mutant For-3	CCCACATCACTGTGTTTACTTACTG AGCACCCTCTTGGTG	Metabion
Murine *Ntn1* 3'-UTR mutant Rev-3	AAACACAGTGATGTGGGCAGAAGT GGAG	Metabion
Murine *Tagln* 3'-UTR For	ATTGAATTCGCCTGCCTCACAAAT GCCTATG	Metabion
Murine *Tagln* 3'-UTR Rev	ATTCTGCAGTGGGCTGGGTCTCCT TCAAAGG	Metabion
Murine *Tagln* 3'-UTR mutant For-1	AGCCACTGTGTTTCTGGCCCCTGT TCCCAGCT	Metabion
Murine *Tagln* 3'-UTR mutant Rev-1	CCAGAAACACAGTGGCTCTGGGGT AAGATGCT	Metabion
Murine *Tagln* 3'-UTR mutant For-2	AGCCTGGCTGTAGGCCAGCCCACT GTCCTT	Metabion
Murine *Tagln* 3'-UTR mutant Rev-2	TGGCCTACAGCCAGGCTACCCCAG C	Metabion
Human *NTN1* 3'-UTR For	ATTGAATTCGTCTCCACTGCTACCT GCTG	Metabion
Human *NTN1* 3'-UTR Rev	ATTGATATCTCCCACAGGGTTGTC ATGAG	Metabion
Human *NTN1* 3'-UTR mutant For-1	GTGGTCACCGCCTCATGCTGGAGC TGCC	Metabion
Human *NTN1* 3'-UTR mutant Rev-1	CATGAGGCGGTGACCACCCGGCT TGGG	Metabion
Human *NTN1* 3'-UTR mutant For-2	TCTCTGTGTGTTTCGGGCCTCTGG CCCACAT	Metabion
Human *NTN1* 3'-UTR mutant Rev-2	CCCGAAACACACAGAGAGCCCAG GAAGGCA	Metabion
Human *TAGLN* 3'-UTR For	ATTGAATTCCTTAGCCTGCCTCACC CACAC	Metabion
Human *TAGLN* 3'-UTR Rev	ATTGATATCACGGCAGCCAGGAAC ACATAC	Metabion

Human *TAGLN* 3'-UTR mutant For-1	GCATTGTGTTTTTGGCCCCTCCCT CCCGG	Metabion
Human *TAGLN* 3'-UTR mutant Rev-1	GGCCAAAAACACAATGCTTTCGGG TAAGAAGTTGG	Metabion
Human *TAGLN* 3'-UTR mutant For-2	TTTGCCCTGGTCACTTTTGTTATGG TTTCAGATCTG	Metabion
Human *TAGLN* 3'-UTR mutant Rev-2	AAAGTGACCAGGGCAAATCAAACT CTGCCA	Metabion
pre-miR-34a oligo	GGCCAGCUGUGAGUGUUUCUUUG GCAGUGUCUUAGCUGGUUGUUGU GAGCAAUAGUAAGGAAGCAAUCA GCAAGUAUACUGCCCUAGAAGUG CUGCACGUUGUGGGGCCC	Thermo Fisher Scientific

Table S5, related to Materials and Methods.

List of primers used for qPCR.

**Name**	**Sequence (5' - 3')**
Mouse-*Cyclophilin*-for	ATGGTCAACCCCACCGTGT
Mouse-*Cyclophilin*-rev	TTCTGCTGTCTTTGGAACTTTGTC
Mouse-*β-actin*-for	CTAAGGCCAACCGTGAAAAG
Mouse-*β-actin*-rev	ACCAGAGGCATACAGGGACA
Mouse-*B2M*-for	CCGGCCTGTATGCTATCC
Mouse-*B2M*-rev	CTTGCTGAAGGACATATCTGACA
Mouse-*Csf1r*-for	CCCCACAGATAAAATTGGAGCC
Mouse-*Csf1r*-rev	TTGAATCCCACTTCGGCGTT
Mouse-*pri-mir-34a*-for	CTGTGCCCTCTTGCAAAA
Mouse-*pri-mir-34a*-rev	GGACATTCAGGTGAGGGT
Mouse-*Dkk2*-for	CGGCATAGAGATCGCAACCATG
Mouse-*Dkk2*-rev	GCAGTCTGATGACCGTAGGCAT
Mouse-*Fzd10*-for	CTGGCTTGCTACCTAGTCATCG
Mouse-*Fzd10*-rev	TGCGTACCATGAGCTTCTCCAG
Mouse-*Wnt10a*-for	GCTCCTGTTCTTCCTACTGCTG
Mouse-*Wnt10a*-rev	ATGTCAGGCACACTGTGTTGGC

Mouse-*Clec16a*-for	GAACACCACAGACGAGGAGAAG
Mouse-*Clec16a*-rev	CATACAGGAGGCAGAGCACGAA
Mouse-*Slc14a1*-for	TGGCTGTGTAGGAACTGTGGTC
Mouse-*Slc14a1*-rev	GGTGGCATTGTAACCTTGGAGC
Mouse-*Ptprm*-for	AGAGGAAGGAGACGATGAGCAG
Mouse-*Ptprm*-rev	AGAAGGCTTCGTCGCAGTTGGT
Mouse-*Ntn1*-for	GTCTGGTGTGTGACTGTAGGCA
Mouse-*Ntn1*-rev	CCGAGCATGGAGGTTGCAGTTG
Mouse-*Tagln*-for	ATATGGAGCCTGTGTGGAGTG
Mouse-*Tagln*-rev	CACTGGCTTCGATCCCTCAG
Mouse-*Dab2*-for	CTCTTCAAAGGCAATGCTCCTGC
Mouse-*Dab2*-rev	TATGGCTCCTGGGACCACAGTT
Mouse-*Grem1*-for	AGGTGCTTGAGTCCAGCCAAGA
Mouse-*Grem1*-rev	TCCTCGTGGATGGTCTGCTTCA
Mouse-*Atp2b4*-for	CACCATCTCACTAGCCTACTCTG
Mouse-*Atp2b4*-rev	AGTGTGCCTGTCTTATCGGAGC
Mouse-*Ank2*-for	ATCGGAGTCAGATCAAGAGCCG
Mouse-*Ank2*-rev	AAGCCAGCCTTTCTTCCATCCG
Mouse-*Igf2*-for	CTTCAGTTTGTCTGTTCGGACCG
Mouse-*Igf2*-rev	GTGGCACAGTATGTCTCCAGGA
Mouse-*Notch2*-for	CCACCTGCAATGACTTCATCGG
Mouse-*Notch2*-rev	TCGATGCAGGTGCCTCCATTCT
Mouse-*Epha4*-for	GGCTATACTGACAAGCAGAGGAG
Mouse-*Epha4*-rev	GGAAAGCATCCAAGGAGCCGTT
Mouse-*Sesn3*-for	GCGCATGTATGACAGCTACTGG
Mouse-*Sesn3*-rev	TCAGATGCCGAGTTATGGCTCG
Mouse-*Lef1*-for	ACTGTCAGGCGACACTTCCATG
Mouse-*Lef1*-rev	GTGCTCCTGTTTGACCTGAGGT
Mouse-*Jag1*-for	TGCGTGGTCAATGGAGACTCCT
Mouse-*Jag1*-rev	TCGCACCGATACCAGTTGTCTC
Mouse-*Prickle1*-for	AACAGCTCCTGTACCAGTTGCC

Mouse-*Prickle1*-rev	CTTCCTCTGAGCACTGAACACC
Mouse-*Fgf9*-for	ACAGTGGACTCTACCTCGGCAT
Mouse-*Fgf9*-rev	GGTTGGAAGAGTAGGTGTTGTAC
Mouse-*Npnt*-for	GGAGCTACATCTGCAAGTGTCAC
Mouse-*Npnt*-rev	GCTACACTGGTGCTGTCCAAGA
Mouse-*Adam10*-for	TGCACCTGTGCCAGCTCTGATG
Mouse-*Adam10*-rev	GATAGTCCGACCACTGAACTGC
Mouse-*Notch1*-for	GCAGATGCTCAGGGTGTCTT
Mouse-*Notch1*-rev	GCCAGGATCAGTGGAGTTGT
Mouse-*Snai1*-for	CACACGCTGCCTTGTGTCT
Mouse-*Snai1*-rev	GGTCAGCAAAAGCACGGTT
Human-*TAGLN*-for	CTTCCCTCTGACACATGCGG
Human-*TAGLN* -rev	GTCAGTGGGACACAGTGAGGC
Human-*NTN1*-for	CTGTCCCTCGGCAAGAAGTT
Human-*NTN1*-rev	GTAGATGGCCATGGACTCGG
Human-*β-actin*-for	TGACATTAAGGAGAAGCTGTGCTAC
Human-*β-actin*-rev	GAGTTGAAGGTAGTTTCGTGGATG

Table S6, related to Materials and Methods.

STAT3-related public datasets used to identify potential STAT3 target genes

**Microarray/RNA-seq datasets :**	**ectopic STAT3 expression**	
Homo sapiens	MKN28, const. active STAT3	GSE78714
Mus musculus	mammary tumor, const. active STAT3	GSE17182
Mus musculus	MEFs, const. active STAT3	GSE21507
**Microarray/RNA-seq datasets :**	**STAT3 knockout/knockdown**	
Homo sapiens	DU145 STAT3 siRNA	GSE25944
Homo sapiens	A375 STAT3 siRNA	GSE31534
Homo sapiens	HCC1143 STAT3 siRNA	GSE85579
Homo sapiens	HCC70 STAT3 siRNA	GSE85579
Homo sapiens	MDA231 STAT3 siRNA	GSE85579
Homo sapiens	MDA157 STAT3 siRNA	GSE85579
Homo sapiens	MDA468 STAT3 siRNA	GSE85579
Homo sapiens	LY10 STAT3 siRNA	GSE106844
Homo sapiens	TMD8 STAT3 siRNA	GSE106844
Homo sapiens	Du145 STAT3 siRNA	GSE17482
Homo sapiens	Hela - STAT3 KO	GSE108495
Homo sapiens	SKOV STAT3 KO	GSE134375
Homo sapiens	OV3 STAT3 KO	GSE134375
Homo sapiens	OV8 STAT3 KO	GSE134375
Homo sapiens	CWR STAT3 siRNA	GSE17482
Homo sapiens	SKOV STAT3 siRNA	GSE20597
Mus musculus	STAT3 KO	GSE6846
Mus musculus	STAT3 KO	GSE151447
**STAT3 ChIP-Seq datasets**		
Homo sapiens	Tumor cells	GSM2278006
Homo sapiens	OCI-Ly7; B cell lymphoma; Blood	GSM1227207
Homo sapiens	MCF-10A; Epithelium; Breast	GSM935457
Homo sapiens	MDA-MB-231; mDA	GSM2278002
Homo sapiens	HCC1143	GSM2278010
Homo sapiens	U-2932; B Lymphocyte	GSM1227212
Homo sapiens	OCI-Ly3; B Lymphocyte; Bone Marrow	GSM1227206
Homo sapiens	H358; Lung	GSM2752894
Homo sapiens	SU-DHL4; B Lymphocyte; Peritoneal Effusion	GSM1227210
Homo sapiens	HCC70; Epithelium; Breast	GSM2278004
Homo sapiens	OCI-Ly10; B Lymphocyte; Bone Marrow	GSM1227204
Homo sapiens	MDA-MB-157; mDA	GSM2278001
Homo sapiens	MDA-MB-468; mDA	GSM2278009

Homo sapiens	SU-DHL2; B Lymphocyte	GSM1227209
Mus musculus	Mammary Gland	GSM2300477
Mus musculus	Th17	GSM1004860
Mus musculus	AtT-20; Corticotroph; Pituitary	GSM926625
Mus musculus	Embryonic Stem Cell	GSM2561450
Mus musculus	T Lymphocyte	GSM1601733
Mus musculus	T Lymphocyte; Blood	GSM494691
Mus musculus	Dendritic Cell; Spleen	GSM671415
Mus musculus	AtT-20; Corticotroph; Pituitary	GSM2445278
Mus musculus	T Lymphocyte; Spleen	GSM1543812
Mus musculus	T Lymphocyte; Blood	GSM580756
Mus musculus	in vitro polarized Th17 T cells	GSM540722
Mus musculus	primary CD4+ T cells	GSM652877
Mus musculus	Primary Cortical Oligodendrocyte Progenitor (OLP) cells	GSM2650745

Table S7, related to Materials and Methods.

IL6/STAT3-related public datasets used to identify potential STAT3 target genes

**Microarray/RNA-seq datasets :**	**IL6-treatment**	
Homo sapiens	MCF7 + IL6 1h	GSE126003
Homo sapiens	T47D + IL6 1h	GSE126003
Homo sapiens	DLD1 +IL6 24h	GSE149262
Homo sapiens	CMEC/D3 +IL6 72h	GSE138309
Homo sapiens	airways epithelial cells +IL6	GSE113185
Homo sapiens	macrophages +IL6	GSE123603
Homo sapiens	ANBL6 MM +IL6 24h	GSE115558
Homo sapiens	FLAM76 MM +IL6 24h	GSE115558
Homo sapiens	HUVEC +IL6	GSE163649
Homo sapiens	endothelial cells +IL6 1h	GSE19082
Homo sapiens	macrophages +IL6 4h	GSE8515
Homo sapiens	HepG2 +IL6 4h	GSE411
Homo sapiens	HK2 +IL6 1.5h	GSE68826
Homo sapiens	HK2 +IL6 1.5h	GSE68940
Homo sapiens	keratinocytes +IL6 24h	GSE53751
Homo sapiens	trachea cells +IL6 24h	GSE67361
Mus musculus	macrophages +IL6 6h	GSE411
Mus musculus	hepatocytes +IL6 4h	GSE21031
Mus musculus	liver +IL6 1h	GSE21060
Mus musculus	gastric tumors +IL6 1h	GSE43800
Mus musculus	hepatocytes +IL6 24h	GSE69928
**STAT3 ChIP-Seq datasets**		
Homo sapiens	Tumor cells	GSM2278006
Homo sapiens	OCI-Ly7; B cell lymphoma; Blood	GSM1227207
Homo sapiens	MCF-10A; Epithelium; Breast	GSM935457
Homo sapiens	MDA-MB-231; mDA	GSM2278002
Homo sapiens	HCC1143	GSM2278010
Homo sapiens	U-2932; B Lymphocyte	GSM1227212
Homo sapiens	OCI-Ly3; B Lymphocyte; Bone Marrow	GSM1227206
Homo sapiens	H358; Lung	GSM2752894
Homo sapiens	SU-DHL4; B Lymphocyte; Peritoneal Effusion	GSM1227210
Homo sapiens	HCC70; Epithelium; Breast	GSM2278004
Homo sapiens	OCI-Ly10; B Lymphocyte; Bone Marrow	GSM1227204
Homo sapiens	MDA-MB-157; mDA	GSM2278001
Homo sapiens	MDA-MB-468; mDA	GSM2278009
Homo sapiens	SU-DHL2; B Lymphocyte	GSM1227209
Mus musculus	Mammary Gland	GSM2300477
Mus musculus	Th17	GSM1004860
Mus musculus	AtT-20; Corticotroph; Pituitary	GSM926625
Mus musculus	Embryonic Stem Cell	GSM2561450
Mus musculus	T Lymphocyte	GSM1601733

Mus musculus	T Lymphocyte; Blood	GSM494691
Mus musculus	Dendritic Cell; Spleen	GSM671415
Mus musculus	AtT-20; Corticotroph; Pituitary	GSM2445278
Mus musculus	T Lymphocyte; Spleen	GSM1543812
Mus musculus	T Lymphocyte; Blood	GSM580756
Mus musculus	in vitro polarized Th17 T cells	GSM540722
Mus musculus	primary CD4+ T cells	GSM652877
Mus musculus	Primary Cortical Oligodendrocyte Progenitor (OLP) cells	GSM2650745

Table S8, related to Materials and Methods.

c-JUN-related public datasets used to identify potential c-JUN target genes

**Microarray/RNA-seq datasets :**	**ectopic c-JUN expression**	
Homo sapiens	141 cells cJUN OE	GSE57520
Homo sapiens	510 cells cJUN OE	GSE57520
Homo sapiens	LPS12 cells cJUN OE	GSE57520
Mus musculus	mouse ESC cJUN OE	GSE50776
Mus musculus	mouse MEF cJUN OE	GSE50776
**Microarray/RNA-seq datasets :**	**c-JUN knockout/knockdown**	
Homo sapiens	BT549 cJUN siRNA	GSE71915
Mus musculus	mouse MEF cJUN KO	GSE26205
Mus musculus	mouse ESC cJUN KO	GSE127925
**c-JUN ChIP-Seq datasets**		
Homo sapiens	A549; Epithelium; Lung	GSM2437886
Homo sapiens	K562; Erythroblast; Bone Marrow	GSM935467
Homo sapiens	MDA-MB-231; Epithelium; Breast	GSM3070218
Homo sapiens	MDA-MB-231; Epithelium; Breast	GSM1700785
Homo sapiens	JHU-06; Endothelial Cell	GSM2576177
Homo sapiens	K562; Erythroblast; Bone Marrow	GSM935569
Homo sapiens	hESC; Embryonic Stem Cell	GSM2945834
Homo sapiens	Coronary artery smooth muscle	GSM1503219
Homo sapiens	LoVo; Colon	GSM1239467
Homo sapiens	Calu-3; Lung	GSM2266291
Homo sapiens	MCF-7; Epithelium; Breast	GSM2736190
Mus musculus	BMDM; Bone Marrow	GSM2974800
Mus musculus	Bone Marrow	GSM2974851
Mus musculus	CH12; Lymphoblastoid; Blood	GSM912901
Mus musculus	T Lymphocyte; Blood	GSM978770
Mus musculus	Chondrocyte; Rib	GSM1891979
Mus musculus	3T3-L1; Preadipocyte; Adipose	GSM1370451
Mus musculus	Th17; Spleen	GSM978770
Mus musculus	Myoblast; Muscle	GSM1354747

Table S9, related to Materials and Methods.

SRF-related public datasets used to identify potential SRF target genes

**Microarray/RNA-seq datasets :**	**ectopic SRF expression / SRF induction**	
Mus musculus	MEFs, serum-induction	GSE45888
Mus musculus	MEFs, TPA-treatment	GSE75667
Mus musculus	cardiomyocytes, Srf OE	GSE116030
Mus musculus	Neural progenitor cells, Srf OE	GSE90034
**Microarray/RNA-seq datasets :**	**SRF knockout/knockdown**	
Mus musculus	Srf KO cardiomyocyte, postnatal day 14	GSE109425
Mus musculus	Srf KO cardiomyocyte, postnatal day 90	GSE109425
**SRF ChIP-Seq datasets**		
Homo sapiens	HCT-116; Colon	GSM1010851
Homo sapiens	HUES64; Embryonic Stem Cell; Embryo	GSM1505777
Homo sapiens	ECC-1; Epithelium; Endometrium	GSM1010762
Homo sapiens	MCF-7; Epithelium; Breast	GSM1010839
Homo sapiens	A673; Polygonal; Muscle	GSM2436678
Homo sapiens	H1; Embryonic Stem Cell; Embryo	GSM803425
Homo sapiens	K562; Erythroblast; Bone Marrow	GSM803520
Homo sapiens	GM12878; B Lymphocyte; Blood	GSM803477
Homo sapiens	HepG2; Epithelium; Liver	GSM803502
Mus musculus	Neural Progenitor Cell	GSM2835909
Mus musculus	HL-1; Cardiomyocyte; Muscle	GSM471926
Mus musculus	Macrophage; Bone Marrow	GSM1645124
Mus musculus	MEFs; Embryonic Fibroblast	GSM1963110
Mus musculus	10T1/2; Fibroblast; Embryo	GSM992343
Mus musculus	Smooth Muscle Cell; Muscle	GSM3069844
Mus musculus	HL-1; Cardiomyocyte; Muscle	GSM558907
Mus musculus	NIH-3T3; Fibroblast; Embryo	GSM1118304
Mus musculus	Cornea	GSM1310233
Mus musculus	C2C12; Myoblast; Muscle	GSM915168
Mus musculus	Cornea	GSM1310232

Table S10, related to Figure 6B (upper panel).

List of the significantly up-regulated and down-regulated mRNAs in *Mir34a*-deficient adenomas compared to *Apc*$^{Min/+}$ adenomas.

Significantly up-regulated mRNAs				Significantly down-regulated mRNAs			
Gene symbol	Base mean	Log_2 fold change	padj	Gene symbol	Base mean	Log_2 fold change	padj
Rps3a3	7365.848	7.900	5.03E-192	*Gabarapl2*	3202.215	-0.303	0.029481797
Fabp6	767.475	6.368	0.01538984	*Ergic3*	5896.202	-0.307	0.004985113
Plb1	1915.384	6.354	0.022477423	*Spns1*	1388.700	-0.316	0.027956778
Defa2	1181.559	6.155	0.000655073	*Irf2*	2226.794	-0.321	0.039577983
Rpl3-ps1	3154.752	5.619	7.35E-29	*Prdx1*	43626.677	-0.328	0.013437061
Ighv1-14	88.538	5.483	0.004199661	*Gstp1*	9014.622	-0.333	0.013909296
Gm15308	4457.504	5.439	0.003596828	*Ndufs2*	11298.639	-0.370	0.045873581
Rps15a-ps8	276.792	5.394	0.002694744	*Ifngr2*	8121.770	-0.371	0.046564398
Rps3a2	4774.443	5.315	8.82E-134	*Pias4*	1121.766	-0.373	0.047914688
Gm3608	805.717	4.473	3.25E-17	*Cldn3*	8208.716	-0.376	0.036137412
Rps13-ps1	1983.568	4.431	0.007955448	*Tarbp2*	1136.451	-0.378	0.031695187
Defa21	3706.476	4.322	0.006304795	*Yeats4*	1733.899	-0.383	0.012563872
AA465934	77.127	4.297	2.13E-20	*Clta*	7721.149	-0.397	0.009153475
Defa22	3855.396	4.265	0.002466613	*Reps1*	1045.399	-0.428	0.030962392
Defa20	8548.141	4.142	0.004196561	*Atox1*	1836.608	-0.429	0.028218178
Gm7861	3360.437	4.073	0.000323885	*Cers2*	5373.356	-0.441	0.01538984
Art2a-ps	183.008	3.650	8.50E-13	*Pgap2*	1416.924	-0.442	0.006777366
Gm45187	65.610	3.650	0.001411018	*Gadd45a*	1024.789	-0.448	0.009020599
Gm14851	1259.746	3.649	0.000145422	*Rpl23a-ps3*	10540.275	-0.453	0.029481797
Defa26	114.578	3.503	0.000417858	*Ptms*	8250.755	-0.454	0.043934443
Ighv1-79	14.107	3.476	0.01538984	*Mknk2*	5343.088	-0.463	0.014760848
Myo18b	134.365	3.398	6.65E-25	*Chp1*	12646.494	-0.464	0.007879368
BC021767	64.456	3.391	0.001147298	*Erbb2*	3870.487	-0.479	0.033014213
Aqp4	261.910	3.359	1.54E-07	*Slc22a18*	2124.810	-0.485	0.048792327
Defa-rs1	1201.228	3.163	2.14E-11	*Atg2a*	1473.816	-0.488	0.008790228
Miat	22.298	3.108	0.002253683	*Mrpl48*	1607.691	-0.489	0.013402095
Defa5	2904.156	3.066	0.01475314	*Gins4*	846.685	-0.490	0.011241874
Gm14850	6012.373	3.039	0.035921244	*Hmgb1*	4378.909	-0.490	0.000655073
Hand1	27.773	3.007	0.01278381	*Prr15l*	4483.967	-0.498	0.009259377
Nrcam	42.075	2.995	0.00586178	*Sdf2*	2426.141	-0.518	0.012190125
Thbs4	83.274	2.955	0.020008038	*Gsto1*	11944.443	-0.523	0.021332972
AY761184	1350.587	2.947	0.027503502	*Tpra1*	959.220	-0.524	0.014760848
Defa3	550.794	2.911	0.02480173	*Ccdc34*	4462.785	-0.525	0.000782987
Kcne4	199.865	2.890	0.007303028	*Irf6*	7954.086	-0.535	0.011338462
Duox1	26.797	2.844	0.002836718	*Prune*	1687.158	-0.542	2.91E-05
Golga7b	25.428	2.817	0.004387201	*1700037H04Rik*	874.154	-0.546	0.045873581
Lipf	657.991	2.750	0.036908696	*Mrpl9*	2542.514	-0.559	0.000380263
Mamdc2	20.509	2.746	0.011941856	*Lfng*	859.884	-0.559	0.02934946
Gpr182	156.057	2.742	7.82E-10	*Rnf41*	972.869	-0.564	0.027503502
Dgkb	29.136	2.692	0.01250952	*Phldb3*	328.312	-0.571	0.037543532
Calb2	19.154	2.634	0.006097704	*Cdc42ep5*	2133.673	-0.578	0.025068965
Rfx6	37.882	2.583	0.030360614	*Plin2*	3099.749	-0.588	0.002088621
Pcdhgb7	62.021	2.582	0.023879709	*Tcaf2*	1069.726	-0.589	0.00238409
Scn7a	72.802	2.579	0.003431915	*Grb7*	3886.749	-0.605	0.009630487
Grem1	2077.216	2.567	0.018053765	*Fbln1*	3926.405	-0.611	0.008493992
Gm20633	26.420	2.558	0.025806832	*Gm19680*	629.006	-0.618	0.041547738
Gm15284	4958.415	2.554	0.01250952	*Atg101*	1338.362	-0.621	0.007961059
Kcna2	34.843	2.538	0.009144102	*Krt23*	8932.446	-0.622	0.034723281
Angptl1	20.343	2.514	0.008806327	*Mettl23*	485.021	-0.644	0.037857791
Scgb3a1	19.532	2.486	0.00689615	*Aamdc*	916.824	-0.648	0.004578594
Susd2	1132.678	2.446	0.02934946	*Ing4*	1571.023	-0.658	0.00173874
Retnlb	332.232	2.417	2.35E-05	*St3gal6*	1880.357	-0.659	0.032240436
Tmem116	34.355	2.416	0.000245695	*Apobec3*	3317.659	-0.667	0.019296155
Scara5	53.498	2.414	0.001512289	*Shisa2*	1372.872	-0.688	0.001646867
Tmem100	33.667	2.409	0.000481879	*Rarg*	694.579	-0.710	0.009020599
Chrm2	171.085	2.345	0.004196561	*Capza1*	7491.254	-0.714	0.018573937
Scn3b	93.753	2.345	0.006795926	*Slc26a6*	4021.419	-0.714	0.013402095
Mfap4	133.454	2.313	8.80E-05	*Eif3j1*	1293.122	-0.716	0.013192269
Kcnn3	38.473	2.286	0.002203296	*Rpl28*	10034.868	-0.719	0.001462614
Slit3	99.178	2.284	5.76E-06	*Nectin4*	653.866	-0.721	0.024939507
Lgr6	39.258	2.284	0.009153475	*Ptgr1*	11085.631	-0.724	0.007876924
Slc22a3	38.608	2.277	0.014064026	*Zfpm1*	3487.555	-0.728	0.000199802
Kcnip2	27.307	2.270	0.040486325	*Cdc25b*	1056.552	-0.730	0.012586633
6330403A02Rik	65.759	2.268	0.000265291	*Rpl3*	36457.453	-0.735	0.000664812
Ackr1	38.321	2.264	0.008687209	*Ppfibp2*	1386.982	-0.749	0.009020599
Gcg	89.144	2.260	9.86E-05	*Ovol2*	398.038	-0.763	0.035524674
Fbln2	128.008	2.256	1.25E-05	*AA467197*	5407.425	-0.775	0.01191737
Pgm5	344.438	2.237	6.07E-06	*Klhl42*	882.789	-0.788	0.030360614
Diras2	29.314	2.220	0.008493992	*Sytl4*	215.104	-0.791	0.012035597
Tnfrsf8	14.841	2.219	0.009020599	*Scnm1*	685.689	-0.807	0.000499371
Vstm2b	53.779	2.219	0.045873581	*Gm42528*	215.563	-0.876	0.034723281
Adamtsl3	50.448	2.211	0.010044251	*Rplp0*	60447.318	-0.879	0.033505958
Sdk1	32.962	2.210	0.027503502	*Rhebl1*	169.444	-0.893	0.039189471
Chrdl1	107.589	2.198	0.023700698	*Ltbp4-1*	2946.853	-0.909	0.034223382
Olfm4	1117.010	2.197	0.000180627	*Epn3*	992.638	-0.914	0.004728021
Hsd11b1	145.683	2.191	2.25E-06	*Pop4*	934.059	-0.925	2.09E-08
Kcnma1	39.678	2.189	0.006171304	*Gsta4*	2253.318	-0.933	0.017731618
Ildr2	215.074	2.181	0.002088621	*Gnal*	1382.861	-0.940	0.001540291
Popdc2	47.047	2.174	0.034723281	*Ckb*	6138.712	-0.957	0.017165872

Defa17	8516.947	2.172	1.61E-06
Slc14a1	456.593	2.170	0.027503502
Chrdl2	79.967	2.168	0.02682833
Mgp	177.467	2.166	0.000792201
Hcar1	27.790	2.164	0.017165872
Myh11	3067.121	2.153	0.000414054
Serpina3f	119.859	2.150	0.040486325
Wscd2	23.518	2.148	0.030689281
Bmpr1b	11.583	2.142	0.040486325
Fgfbp1	190.898	2.141	0.000732548
Fmo2	207.033	2.136	0.035524674
Defa24	14430.425	2.133	1.74E-05
Fgl1	47.000	2.116	0.043273222
Pyroxd2	139.202	2.108	5.62E-05
Cd177	2520.281	2.094	5.94E-06
Chl1	58.221	2.093	0.0010807
Cadm3	45.212	2.064	0.000198378
Ifi205	88.261	2.058	1.71E-08
Pln	42.448	2.057	0.025068965
Egfl6	68.381	2.035	0.037466502
Exd1	39.342	2.033	0.000128272
Adamts8	36.693	2.019	0.007876924
Arhgap44	77.808	1.992	0.000481879
Rspo3	79.339	1.966	0.00230325
Adcy5	124.989	1.963	0.002029719
Hmcn2	129.101	1.933	0.009630487
Cxcl12	571.126	1.917	0.001150611
St6galnac3	39.810	1.911	0.009306822
Fam107a	21.798	1.876	0.012331324
Clip4	59.985	1.869	0.001641294
Plscr2	71.482	1.869	0.002612845
Gm14434	128.696	1.868	0.009115883
Vip	278.915	1.861	0.000193318
Gfra1	126.998	1.849	0.000582725
Sgip1	28.069	1.839	0.005147696
Mptx2	2685.756	1.834	0.005050337
Lrrn1	51.574	1.833	0.014213123
Slc24a3	163.056	1.825	0.009354026
Dpt	184.713	1.797	0.023879709
Sspn	92.837	1.793	9.85E-07
4930481B07Rik	39.901	1.793	0.046988981
Lama2	49.077	1.787	0.036740585
P3h2	67.029	1.780	0.031695187
Synm	619.552	1.773	0.002232397
Clca3a1	230.328	1.765	0.035436368
Slc5a12	1187.123	1.753	0.027581575
Ogn	111.501	1.733	0.046312028
Gm16685	29.011	1.722	0.022957788
Ceacam10	616.562	1.710	0.000792201
Scg3	27.950	1.708	0.030360614
Ldhb	293.015	1.702	6.05E-06
Crispld2	439.633	1.701	0.005944366
Ror1	49.156	1.692	0.040486325
Rims1	164.029	1.688	0.025068965
Sfrp1	365.529	1.688	0.000433835
Rdh16	399.418	1.687	0.026866872
Actg2	2107.656	1.682	0.027503502
Lgi2	135.524	1.663	0.025806832
Tbx1	349.553	1.659	3.67E-06
Lipg	161.669	1.654	0.041007313
Cnn1	906.427	1.652	0.026062685
Nkd2	79.037	1.652	0.009354026
Tchh	113.748	1.650	2.41E-05
Galnt15	59.749	1.631	0.030962392
Tnxb	119.838	1.631	0.005682432
Hoxb8	28.826	1.630	0.027503502
Gpm6a	31.285	1.601	0.025621987
Fibin	48.407	1.601	0.049876245
Col8a1	81.304	1.589	0.010542741
Cygb	256.459	1.580	0.007694842
Mrgprf	38.265	1.571	0.006304795
Mmrn1	269.169	1.566	0.011338462
Wnt2b	51.620	1.561	0.006467812
Slc7a11	468.273	1.546	0.007879368
Pcdh20	71.666	1.541	0.017165872
Phactr1	42.458	1.537	0.03856856
Tmem252	437.823	1.535	0.025590556
Slc1a1	401.313	1.527	0.000713188
Cap2	149.616	1.526	0.014975205
Tgfbr3	208.448	1.519	0.020749332
Tgm4	139.989	1.518	0.041336149
Tpm2	2129.924	1.514	0.027754834
Ak1	125.795	1.505	0.001512289
Jph2	199.656	1.499	0.00989613
Rgs7bp	81.145	1.489	0.000200618
Kcnmb1	96.798	1.476	0.020749515
C4b	1110.783	1.468	0.002760684
9130208D14Rik	664.824	1.465	0.046922751
Adgrd1	428.596	1.464	0.01250952
Svep1	63.346	1.461	0.005050337
Col23a1	135.872	1.461	0.005653967
Atp1a2	272.819	1.459	0.014345091
Wnt10a	52.028	1.457	0.031681957
Ntn1	180.815	1.454	0.041329523
Frzb	67.584	1.452	0.017165872
Il18r1	32.299	1.448	0.027087442
Pccb	4874.959	-0.960	8.08E-05
Foxq1	2665.362	-1.002	0.034223382
Plcd3	525.744	-1.025	0.002775875
Rbbp8nl	144.988	-1.050	0.041007313
Prss12	1416.146	-1.084	0.000631404
4930523C07Rik	763.511	-1.086	0.001240333
Sord	6041.751	-1.090	0.014345091
Jmjd7	190.205	-1.093	0.014064026
Slc46a1	2152.544	-1.101	0.008724678
Fer1l4	931.099	-1.102	0.029481797
T	1084.098	-1.117	0.014064026
Hist1h2bc	3224.411	-1.118	0.012331324
Hist2h2be	252.395	-1.208	0.023134605
Aadac	1301.692	-1.230	0.030962392
Prr18	1073.154	-1.232	8.34E-05
Cyp2c68	991.413	-1.315	0.019743052
Hr	1810.114	-1.320	0.044858355
2310058D17Rik	36.065	-1.332	0.035243839
2210407C18Rik	11009.390	-1.376	0.020749515
Car2	1009.671	-1.403	0.046094557
Grhl3	703.120	-1.430	4.69E-05
Spag4	37.618	-1.471	0.027503502
1700020L24Rik	208.739	-1.608	0.027581575
Gm13408	91.275	-1.610	0.007879368
Mmp28	600.427	-1.641	0.002754647
Nlrp10	339.620	-1.650	0.001512289
5830444B04Rik	90.065	-1.652	0.005218856
RP23-359B23.11	535.444	-1.669	0.025418747
Ighv1-26	618.296	-1.673	0.041664063
Rn18s-rs5	248.780	-1.706	0.009203985
Tmprss11e	160.150	-1.886	0.001937704
Ighv1-85	220.677	-2.036	0.013402095
Acat3	66.353	-2.041	0.01002361
Slc9a4	154.024	-2.057	0.030251791
Gm5586	64.144	-2.120	0.0010807
Ighv1-72	346.927	-2.156	0.006304795
1500015A07Rik	486.008	-2.158	7.35E-29
Cldn18	516.076	-2.171	0.008724678
Ighv5-4	238.273	-2.224	0.005218856
Pgc	278.417	-2.311	0.006328943
Ivl	244.012	-2.333	0.009306822
Igkv17-121	331.207	-2.368	0.017165872
Rps3a1	30160.999	-2.394	2.90E-45
Ighm	177.569	-2.597	0.017552181
Anxa9	66.465	-2.848	1.10E-05
Tff1	315.259	-3.129	0.032889928
Reg1	3434.803	-3.154	0.025068965
Marcksl1-ps4	25.306	-3.486	0.02934946

Slc6a4	664.185	1.443	0.005815348
Rab3c	58.671	1.418	0.003431915
Reg4	20307.094	1.411	0.035436368
Serpina3g	324.859	1.405	0.046090485
Tagln	3364.471	1.403	0.01288103
Colec12	194.425	1.402	6.01E-05
Scpep1	1808.205	1.391	3.56E-08
Atp2b4	442.674	1.387	0.008790228
Nsg2	47.760	1.383	0.034223382
Afap1l1	419.403	1.373	4.85E-08
Madcam1	75.981	1.361	0.024205518
Slc13a2os	42.923	1.357	0.036661955
Ctsk	160.736	1.355	0.042074167
Cacna1e	34.799	1.350	0.034723281
Map3k8	58.561	1.344	0.015838215
Aoc3	174.877	1.342	0.029481797
Slc16a7	66.883	1.338	0.018875724
Ccnjl	244.400	1.320	0.00024649
Bhlha15	144.712	1.314	1.03E-05
Robo2	113.323	1.307	0.034223382
Lcn2	3451.916	1.297	2.86E-10
Gatm	207.704	1.284	0.005850832
Fzd10	473.166	1.281	0.002754647
Spon1	679.469	1.279	0.01538984
Gm8995	148.133	1.270	0.018622276
Islr	125.884	1.263	0.027956778
Scube1	516.106	1.254	0.025068965
Acta2	3799.431	1.251	0.035436368
Gem	398.075	1.248	0.024541839
Lmod1	246.029	1.239	0.015245364
Tmprss6	131.498	1.233	0.044018441
Reln	175.611	1.233	0.036740585
Gm10221	1353.661	1.229	0.049493666
Slc2a13	114.435	1.213	0.035524674
Ppp1r3c	59.817	1.203	0.011161326
Cacna1c	180.309	1.196	0.034723281
Ffar2	247.244	1.189	0.001932919
Abcc9	627.487	1.186	0.038751848
Bpifb5	4212.674	1.180	0.030708576
Cdh11	281.891	1.167	0.009144102
Clmp	550.804	1.161	0.00989613
Alpl	118.604	1.159	0.022581697
Ank2	52.010	1.145	0.037319884
Igf2	233.996	1.137	0.00388279
Aebp1	199.246	1.136	0.039577983
Dab2	348.936	1.134	0.000199329
S1pr3	110.543	1.130	0.012190125
Ly6c1	360.606	1.123	0.01538984
Esyt3	446.819	1.121	0.005302339
Grb10	352.096	1.119	0.045824379
Fxyd6	365.293	1.103	0.038520262
Enpp2	269.676	1.088	0.034723281
Naip6	1361.947	1.078	0.000664812
Map9	183.603	1.077	0.019837731
1810041L15Rik	61.867	1.070	0.038751848
Dennd2a	174.331	1.067	0.035524674
Myom1	97.922	1.064	0.010297742
Ednra	248.043	1.063	0.036661955
Speg	71.379	1.050	0.030345512
Rab15	571.196	1.038	0.004988689
Cps1	6236.874	1.037	0.000169271
Lbp	350.987	1.036	0.000364096
Abhd3	1886.627	1.029	0.021616191
Rasgrp3	271.629	1.028	0.009825326
Dram1	143.411	1.028	0.046090485
Timp3	2118.665	1.026	0.012586633
2610528A11Rik	999.919	1.026	0.03856856
Dlc1	367.866	1.021	0.017165872
Zcchc24	455.524	1.016	0.014064026
Gm8797	4024.288	1.013	0.000180627
Dkk2	749.595	1.012	0.036137412
C3	1230.079	1.005	0.029481797
Ramp2	148.665	0.999	0.009144102
Tmem154	136.608	0.998	0.028315757
Ddr2	416.252	0.996	0.003596828
Rgs5	3589.823	0.995	0.008335317
Tifa	2135.205	0.994	0.014760848
Calcrl	300.951	0.988	7.13E-08
Adgra2	205.363	0.984	0.027246381
Fam129a	744.024	0.977	0.006586479
Ptprm	189.448	0.970	0.030813463
Prickle2	130.732	0.969	0.001932919
Naip3	158.740	0.968	0.046528149
Rab3il1	85.077	0.958	0.036661955
Rdx	912.403	0.952	0.000256631
Bbs12	92.916	0.950	0.026408953
Nr1h4	897.947	0.939	0.002709267
Socs1	131.853	0.926	0.034223382
Scnn1a	296.339	0.904	0.031055812
Trps1	134.942	0.904	0.04636375
Sparcl1	2663.433	0.901	0.037543532
Sorbs1	332.042	0.895	0.00589292
Msr1	312.164	0.891	0.015757774
Aldh1b1	5403.622	0.876	0.004988689
Itih5	419.200	0.838	0.037056702
Xrcc3	154.468	0.835	0.006343772

Lss	1720.971	0.831	0.024205518				
Zeb2	283.027	0.825	0.034723281				
Apod	185.497	0.824	0.016268569				
9130409J20Rik	1488.501	0.791	0.00213228				
Nrbp2	466.891	0.787	6.07E-06				
Itga9	772.020	0.769	0.037319884				
Cuedc1	784.792	0.767	0.019743052				
Grap	211.826	0.759	0.023455023				
Hsd17b7	1653.746	0.750	0.003431915				
Man2a2	647.197	0.735	0.002775875				
Acad10	345.546	0.731	0.00571129				
St3gal1	703.224	0.720	0.043418421				
Frmd6	553.840	0.719	0.003689416				
Sema6b	300.766	0.692	0.027503502				
Heg1	1533.529	0.662	0.019743052				
Mylk	7195.280	0.650	0.034723281				
Fgf1	386.073	0.646	0.030962392				
Kdm2b	915.433	0.639	0.006304795				
Pwwp2a	677.469	0.633	0.000323885				
Jrk	225.394	0.630	0.003689416				
Pqlc3	633.138	0.629	0.000200618				
Tfip11	1944.493	0.536	0.000582725				
Prnp	694.032	0.527	0.032795982				
Ikbip	789.909	0.517	0.045693103				
Fam46c	1216.773	0.495	0.031695187				
Guf1	829.006	0.463	0.027956778				
Clec16a	716.946	0.459	0.013402095				
Thg1l	705.235	0.458	0.036740585				
Rab8b	1037.103	0.445	0.023879709				
Txndc11	1424.988	0.435	0.015757774				

Table S11, related to Figure 6B (middle panel).

List of the significantly up-regulated and down-regulated mRNAs in *Csf1r*-deficient adenomas compared to *Apc*$^{Min/+}$ adenomas.

Significantly up-regulated mRNAs				Significantly down-regulated mRNAs			
Gene symbol	Base mean	Log_2 fold change	padj	Gene symbol	Base mean	Log_2 fold change	padj
Gm5855	79.263	5.220	3.23E-06	*Fam195b*	991.830	-0.451	0.04707577
Myo18b	134.365	3.328	1.93E-27	*Snx33*	514.073	-0.539	0.04912398
Gm10073	284.797	2.487	0.03412056	*Rpl28*	10034.868	-0.581	0.04152231
Art2a-ps	183.008	2.095	0.00086333	*Gm12396*	471.435	-0.652	0.04083381
Igkv4-57	276.786	1.980	2.83E-09	*Sytl1*	365.210	-0.652	0.03078012
Lgi2	135.524	1.784	0.02721687	*Pllp*	3145.911	-0.675	0.00297421
RP23-359B23.11	535.444	1.731	0.03412056	*Tacstd2*	998.534	-0.983	0.03643623
Olfm4	1117.010	1.645	0.02751039	*Dynlt1b*	4265.072	-1.050	0.0122282
Cxcl5	327.741	1.624	0.01837011	*Gm10116*	149.433	-1.159	0.00707855
Ccr10	43.466	1.595	0.04519266	*Rps3a2*	4774.443	-1.208	1.63E-05
Ccdc109b	83.369	1.586	0.04912398	*A930005H10Rik*	136.660	-1.270	0.00689268
Lcn2	3451.916	1.392	3.96E-13	*Gm44364*	427.610	-1.362	0.02262992
Ceacam10	616.562	1.323	0.04707577	*4930452B06Rik*	120.028	-1.410	0.0224567
Pgk1-rs7	4328.375	1.276	0.00299794	*Slc30a2*	7831.799	-1.448	0.01527869
Igkv10-96	1302.762	1.173	0.02944119	*5830444B04Rik*	90.065	-1.508	0.02948249
Zc3h12a	744.107	1.169	0.00305893	*Otof*	270.110	-1.590	0.01123515
Emp3	171.801	1.148	0.01428724	*Prom2*	510.368	-1.613	0.00462801
Igkv1-110	1678.083	1.095	0.02632293	*Gm44639*	23.103	-2.046	0.02191538
Pigr	45894.595	0.963	0.01123515	*Upk3a*	58.324	-2.069	0.04733272
Ccdc88b	894.810	0.939	0.0122282	*4930480K23Rik*	106.807	-2.299	5.85E-05
Msr1	312.164	0.911	0.02721687	*Psca*	75.779	-2.658	0.00037792
Cps1	6236.874	0.876	0.00508292	*Gm3716*	82.965	-4.069	0.00028973
Parp8	110.106	0.857	0.03822121	*Gm5292*	114.493	-4.124	0.00023307
Elovl6	4376.025	0.796	0.006574	*Gdpd3*	744.287	-4.319	4.60E-05
Pqlc3	633.138	0.694	1.52E-05	*Gm10020*	1658.950	-4.611	0.00707855
Tfip11	1944.493	0.614	2.07E-05	*Gm14094*	123.550	-4.882	2.07E-05
Rab8b	1037.103	0.525	0.00689268				
Tbc1d1	2199.426	0.424	0.04774773				

Table S12, related to Figure 6B (lower panel).

List of the significantly up-regulated and down-regulated mRNAs in *Csf1r/Mir34a*-deficient adenomas compared to *Apc*$^{Min/+}$ adenomas.

Significantly up-regulated mRNAs				Significantly down-regulated mRNAs			
**Gene symbol**	**Base mean**	**Log_2 fold change**	**padj**	**Gene symbol**	**Base mean**	**Log_2 fold change**	**padj**
Rps3a3	7365.848	7.080	1.94E-148	*Tet3*	3028.933	-0.398	0.04349876
Rps3a2	4774.443	4.781	1.31E-104	*Snx33*	514.073	-0.599	0.04718123
Myo18b	134.365	3.104	1.60E-19	*2310007B03Rik*	208.929	-0.837	0.00630993
Igkv4-74	228.277	2.848	3.45E-13	*Tst*	1714.678	-0.880	0.00069462
Igkv8-21	338.034	2.234	0.03279428	*Ihh*	2353.000	-1.001	0.02624866
Zik1	47.770	1.831	0.03447439	*Grhl3*	703.120	-1.065	0.04349876
Igkv4-57	276.786	1.468	0.00093579	*Pitx1*	3637.836	-1.084	0.0285036
Dclk1	574.268	1.376	0.03469885	*Rps3a1*	30160.999	-1.088	1.03E-07
Tmem154	136.608	1.204	0.02596738	*Nectin4*	653.866	-1.120	0.00013334
Ighv3-6	676.428	1.128	0.04349876	*Tacstd2*	998.534	-1.185	0.0117698
Lcn2	3451.916	1.120	7.04E-07	*Smtnl2*	126.920	-1.389	0.0174334
Ythdc2	714.383	0.721	0.04065494	*Psca*	75.779	-2.489	0.00713858
Tfip11	1944.493	0.575	0.00080029	*Amd2*	266.155	-2.525	0.01024457
Cacna2d1	598.594	0.540	0.00796881	*Prss56*	56.568	-3.182	0.02468076
Yipf5	2860.125	0.491	0.03164122	*Mrpl23*	56.739	-3.261	0.0174334
				Gpx5	1509.304	-3.309	7.04E-07
				Otof	270.110	-3.610	1.86E-14

Table S13, related to Figure 6D (upper panel).

List of the significantly up-regulated and down-regulated mRNAs in *Mir34a*-deficient tumoroids compared to *Apc*$^{Min/+}$ tumoroids.

Significantly up-regulated mRNAs				Significantly down-regulated mRNAs			
Gene symbol	Base mean	Log_2 fold change	padj	Gene symbol	Base mean	Log_2 fold change	padj
Gm26983	58.479	7.7867	2.13E-06	*Rplp1*	38053.806	-0.4389	0.02686749
Rps15a-ps8	388.681	6.3442	6.85E-26	*Pfdn2*	3141.819	-0.4619	0.039301847
Rps3a3	9251.682	6.3149	0.033622703	*Gm10288*	23568.680	-0.4624	0.01254258
Eif5al3-ps	407.649	6.2895	3.27E-23	*Txn2*	4596.942	-0.4629	0.03226749
Pcna-ps2	288.898	6.2178	0.035074212	*Atp5g1*	3889.207	-0.4699	0.025179762
Rpl3-ps1	7061.173	6.1786	3.47E-73	*Pdlim1*	3650.079	-0.5009	0.043745185
Dkk2	453.441	5.4580	6.61E-11	*Polr2f*	2310.410	-0.5103	0.031096841
Map2	50.565	5.2654	0.00029331	*Yars*	3734.939	-0.5143	0.044491147
Gm10182	3021.766	5.2066	5.50E-05	*Farsb*	3720.800	-0.5321	0.031486728
Rps13-ps1	2526.239	4.9347	1.24E-35	*Exosc5*	1997.158	-0.5329	0.040526834
Gna14	19.121	4.8714	0.017247803	*Mrps35*	2076.005	-0.5401	0.011986291
Zfp462	209.491	4.6563	0.000535601	*Avpi1*	2223.613	-0.5433	0.044447523
Osr2	353.031	4.6128	4.01E-07	*Uqcr11*	2683.728	-0.5457	0.03593397
Gm13067	24.628	4.5651	0.003838555	*Ak2*	6996.502	-0.5471	0.041861089
Gm29865	63.006	4.1001	1.56E-06	*Nars*	12209.446	-0.5560	0.018804648
Lce6a	31.040	4.1000	0.001224934	*Gm12191*	5621.323	-0.5564	0.044447523
Lrrn3	81.537	4.0977	9.01E-07	*Josd2*	965.835	-0.5688	0.047747905
Ncf2	100.728	4.0479	7.67E-05	*Phf10*	2539.081	-0.5741	0.032323626
Sh3tc2	164.916	4.0409	0.000377131	*Cd3eap*	1211.518	-0.5833	0.030189793
Gm4742	17.256	4.0299	0.021414412	*Sepw1*	3144.474	-0.5844	0.009181118
Prickle1	102.734	3.9337	3.87E-08	*Bpnt1*	3236.641	-0.5868	0.007005518
Pcdh7	133.184	3.9325	2.76E-05	*Ifrd2*	3023.251	-0.5956	0.017055556
Col14a1	201.523	3.8356	0.00040056	*Tspan13*	1561.084	-0.6035	0.017247803
Adamts16	65.187	3.8186	0.008705004	*Htra2*	1798.309	-0.6037	0.006506994
Cd1d2	19.639	3.7583	0.021414412	*Rdm1*	864.919	-0.6062	0.033216916
Clstn2	124.617	3.6177	0.012789462	*Ndufc2*	3251.713	-0.6075	0.016166281
Gm10167	119.470	3.5994	5.36E-09	*Sars*	9547.795	-0.6082	0.008705004
Gm20765	30.015	3.5701	0.038940547	*Cisd3*	3970.507	-0.6089	0.020028688
Gm12669	217.052	3.5623	0.006482775	*Pla2g16*	1816.482	-0.6106	0.005569682
Tmprss13	233.005	3.5214	6.94E-08	*Mrpl48*	1501.526	-0.6201	0.044491147
Syt14	41.957	3.4989	0.002399799	*Lgals9*	3997.136	-0.6225	0.025439644
C2cd4b	53.132	3.4960	0.003073485	*Ablim1*	8085.867	-0.6251	0.028181189
Krt6a	622.517	3.3619	1.95E-07	*Adi1*	2129.068	-0.6357	0.032846535
Irx5	654.455	3.3569	8.77E-07	*Fau*	5507.797	-0.6359	0.004346964
5830418P13Rik	29.126	3.3070	0.010918488	*Hspe1*	6125.856	-0.6373	0.00029331
Wnt10a	134.731	3.2749	7.23E-06	*Sephs2*	6156.994	-0.6399	0.007281661
A730046J19Rik	20.756	3.2640	0.031145921	*Slc25a33*	587.962	-0.6475	0.046495889
RP23-145I16.5	350.152	3.1835	0.000857117	*Etfb*	3480.838	-0.6508	0.00608095
Ism1	66.649	3.0383	0.001851081	*Stc2*	3233.118	-0.6572	0.044447523
Sema3e	46.535	2.9431	0.017645334	*Ccdc115*	959.053	-0.6577	0.023385194
Gpr157	136.064	2.9149	2.01E-06	*Srm*	2837.704	-0.6725	0.02074829
Sema3a	20.760	2.9028	0.044491147	*Slc1a5*	8623.988	-0.6786	0.010463607
H2-Q1	76.012	2.9001	0.000442197	*Pgm1*	2955.085	-0.6830	0.00437696
Ldb3	148.811	2.7480	0.008773847	*Comtd1*	941.625	-0.6882	0.008705004
AA465934	54.151	2.7433	0.000199587	*Psat1*	10218.800	-0.7085	0.022622615
Rassf4	342.221	2.7352	0.00017682	*Eif4ebp1*	2434.218	-0.7108	0.017375236
Col16a1	348.416	2.6824	5.65E-06	*Aldh18a1*	4267.609	-0.7233	0.022622615
Hydin	54.207	2.6007	0.022622615	*Mrps14*	1319.839	-0.7265	0.001851081
Znf41-ps	118.132	2.5969	0.000226429	*Stra13*	1024.293	-0.7281	0.024255873
Tpbg	262.197	2.5805	7.57E-07	*Ankrd54*	1071.513	-0.7291	0.002533876
Gm12603	116.628	2.5030	0.011619079	*Rangrf*	932.487	-0.7395	0.008320802
Crnde	233.410	2.5025	0.019067493	*Uqcrq*	4420.500	-0.7404	0.001084068
Gm20463	77.245	2.4822	0.00044797	*Gsdmd*	5121.295	-0.7415	0.017247803
Foxc1	135.177	2.4768	0.014067655	*Abhd14b*	804.064	-0.7447	0.044447523
Cald1	235.175	2.4538	7.10E-05	*Hacd1*	580.779	-0.7524	0.008705004
Htra1	58.451	2.4441	0.005027141	*1700021F05Rik*	743.671	-0.7640	0.00438845
Pla2g4c	131.244	2.4269	0.00634789	*Paox*	1104.511	-0.7659	0.02857225
Alox12b	34.856	2.4038	0.038461557	*Bcat2*	3863.999	-0.7695	0.005720174
Dpysl3	1071.417	2.3457	1.99E-12	*Mib2*	1440.506	-0.7833	0.039139573
Cubn	189.357	2.3409	0.024723452	*Ldhd*	709.184	-0.7901	0.023877748
Bmp3	143.041	2.3184	0.006394847	*Creg1*	1175.404	-0.7967	0.021093708
St6gal1	576.819	2.3022	1.31E-06	*Vkorc1*	499.561	-0.7980	0.009621267
Map9	73.987	2.2764	0.003995557	*Shmt2*	8341.442	-0.8005	0.001851081
Tenm4	1017.163	2.2691	1.25E-07	*Fam195a*	559.201	-0.8018	0.038582858
Golga7b	475.245	2.2624	1.55E-07	*Aldh2*	10127.622	-0.8041	0.030315134
Cldn8	448.057	2.2237	0.047326482	*Alg8*	1148.273	-0.8088	0.026951216
Grap	127.581	2.2232	5.67E-06	*Palld*	1415.799	-0.8168	0.045827358
Arhgap44	637.247	2.2028	4.23E-08	*Ifi27l2b*	898.811	-0.8213	0.002872761
Vim	3736.819	2.1884	9.87E-05	*Cd63*	7825.030	-0.8334	0.017645334
Neurl1a	408.601	2.1805	0.000951084	*Slc18a1*	546.427	-0.8381	0.042408344
Dst	784.736	2.1662	7.22E-05	*Slc12a8*	1296.432	-0.8390	0.000517548
Scube1	254.954	2.1612	0.002540607	*Rpp25*	401.410	-0.8401	0.045979893
Irx3	66.315	2.1591	0.02314818	*Gm15459*	8150.535	-0.8479	0.025941192
Gm28036	366.737	2.1545	0.007841188	*Aamdc*	394.177	-0.8761	0.007005518
Ccdc80	533.932	2.1180	0.000277774	*Iah1*	931.838	-0.8809	0.01144003
Mreg	60.312	2.1104	0.046880565	*Rps15a*	18242.775	-0.8809	0.0001203
Zc3h12c	495.719	2.1073	2.21E-09	*Gas5*	12386.082	-0.8864	5.25E-05
RP23-457I3.2	223.702	2.1043	0.00628501	*Ifitm3*	22541.062	-0.9191	0.004900801
Fgf9	101.482	2.0671	0.013851881	*Gstm1*	6780.965	-0.9263	1.82E-05
Atp7a	148.126	2.0521	0.044491147	*Mocs1*	1177.157	-0.9298	0.027529268

Sytl2	173.480	2.0187	0.006394847	Cbr1	2799.021	-0.9358	0.016536621
Tnfrsf11b	2360.995	2.0046	1.87E-10	Pck2	4748.555	-0.9458	8.09E-05
Ccnjl	98.451	2.0013	0.002360541	Tada2a	1147.561	-0.9552	0.000339326
Wnt6	1342.586	1.9839	0.003112336	Mtfp1	505.204	-0.9609	0.025941192
Gm38394	961.573	1.9562	0.009621267	Car12	979.140	-0.9641	0.003540452
Shroom4	178.786	1.9402	0.026951216	Gm26825	61907.963	-0.9710	1.49E-07
4933404O12Rik	106.656	1.9371	0.00608095	Gpt2	530.673	-0.9735	0.022633197
Fzd10	328.381	1.9228	0.007069839	Mthfd2	2306.739	-0.9916	1.31E-05
Lifr	290.619	1.9062	0.032767491	Slc6a9	1851.847	-0.9916	0.041861089
Arhgap6	259.923	1.9023	0.009710561	Retsat	1850.948	-0.9997	0.000845633
Myh10	302.782	1.8871	0.030258457	Homer2	2889.558	-1.0006	0.013223948
Rdh9	777.053	1.8738	1.69E-10	Myo1a	4369.252	-1.0129	0.016228803
Gm10676	66.753	1.8565	0.045811139	Atp2a3	788.180	-1.0291	0.043027834
R3hdml	326.455	1.8513	0.011986291	Fuom	253.021	-1.0540	0.015409889
Gcnt1	348.333	1.8203	0.002586935	Camkk2	451.907	-1.0580	0.021074871
Apcdd1	2075.022	1.8154	0.008705004	Prr18	478.717	-1.0713	0.037051369
Lef1	234.227	1.8088	0.000315284	Ggh	685.781	-1.0778	0.003798507
Enpp2	201.155	1.7969	0.009212052	Galnt6	637.388	-1.0882	0.026951216
Ptprd	2082.306	1.7372	9.57E-09	Gm8355	6799.964	-1.0959	0.042305672
Entpd3	1195.274	1.6964	0.001323435	Fabp2	693.495	-1.1017	0.020560865
Slc16a10	682.693	1.6864	4.46E-05	Asns	5033.910	-1.1032	0.000946951
Fam89a	321.894	1.6851	0.000517548	Cbx7	2098.511	-1.1244	9.40E-07
Hspa8	35831.502	1.6831	1.45E-10	Gsto1	17551.616	-1.1913	0.041770402
Gchfr	76.956	1.6731	0.047442082	Reep6	2576.645	-1.2026	0.000377131
Cd244	155.590	1.6603	0.009784393	Pfkfb4	280.570	-1.2163	0.004467261
Klhl23	77.846	1.6510	0.021506823	Ppm1h	268.301	-1.2230	0.000407839
Sema5a	933.533	1.6253	8.36E-07	Abcg2	197.057	-1.2414	0.04771045
Hspb1	415.251	1.6239	0.002677615	Gpd1	1454.474	-1.2708	0.000179201
Nuak1	145.986	1.6185	0.007005518	Hpdl	276.112	-1.2941	0.04771045
Mmp7	12865.237	1.6130	1.87E-10	Foxa2	480.382	-1.2960	0.014962492
Mecom	1168.648	1.5897	1.07E-10	Trib3	452.548	-1.2983	0.000105424
Nr4a2	365.375	1.5884	0.028630607	Tmem158	693.071	-1.3071	0.00011563
Wdfy1	1219.487	1.5460	4.83E-06	Slfn9	242.798	-1.3140	0.0351581
Cd1d1	227.936	1.5404	0.013629106	Dync2li1	172.505	-1.3270	0.033345276
Irs1	210.175	1.5232	0.011986291	Psph	1242.742	-1.3478	3.14E-06
Map6	790.800	1.5231	0.001226583	Apob	1304.153	-1.3750	0.002465045
Plagl1	476.784	1.5219	0.04771045	Ptgr1	14991.733	-1.3912	0.002424496
Esyt3	97.694	1.5182	0.021968632	Grb14	174.911	-1.4271	0.03141421
Mm2610528A11Rik	283.786	1.5101	0.009420265	Itpripl2	689.463	-1.4348	0.026819819
Ephb6	566.105	1.5079	0.004711752	Gstm2	730.663	-1.4493	0.00702117
Gdpd3	1422.754	1.4990	0.009673225	Tm6sf2	150.051	-1.4720	0.015236993
Tbx1	2699.388	1.4848	0.031486728	Tmie	147.575	-1.4915	0.038582858
Cep112	256.866	1.4838	0.00376951	St3gal6	1449.498	-1.4952	0.001851081
Ccser1	340.704	1.4759	0.007131688	Epdr1	334.421	-1.5181	0.021494353
Mtmr11	740.967	1.4281	1.59E-06	Agr2	2132.400	-1.5266	0.000123572
RP24-390A22.1	939.211	1.4092	0.028630607	Atf5	2080.136	-1.5354	1.92E-08
Slc17a4	594.914	1.3995	0.000187708	Rpl3	67237.730	-1.5519	2.80E-26
Mtmr7	299.410	1.3851	0.010034771	Aspa	1155.638	-1.5672	0.002829565
Cdo1	2147.931	1.3732	0.007841188	Cth	1414.812	-1.6361	4.64E-07
Slc22a1	309.731	1.3672	0.005559578	Slc7a3	80.778	-1.6496	0.024412673
Kcnu1	298.891	1.3517	0.001740226	Anxa13	1085.666	-1.6500	0.031793623
Fgfr3	289.820	1.3397	0.026951216	Cbx6	3439.443	-1.6879	1.58E-07
Tcf4	1757.854	1.3391	0.002335503	Pard3b	294.829	-1.7039	0.023997461
Frmd6	809.722	1.3317	2.81E-06	Lgals1	189.230	-1.7053	0.031793623
Gpcpd1	788.994	1.3171	7.11E-06	Glt1d1	193.059	-1.7113	0.008705004
Gpc1	1164.617	1.3135	0.001593348	Rpl11	2971.549	-1.7943	0.000385464
Casp12	234.405	1.2904	0.031486728	Gsta1	2811.844	-1.8094	2.99E-06
Clec16a	532.059	1.2598	0.003557959	Apobec3	3684.982	-1.8317	8.87E-16
Zbtb20	2818.947	1.2440	0.003253948	Muc6	318.750	-1.8736	4.92E-06
Mdfic	336.098	1.2433	0.02141478	Sox17	7712.417	-1.9922	1.49E-05
Pkhd1	261.251	1.2274	0.026836288	Vwf	1279.576	-1.9982	0.007516341
Ceacam1	7830.912	1.2195	0.024255873	Etv5	1381.747	-2.0014	7.14E-11
Slc5a9	868.032	1.2151	0.010376921	Gstm3	679.570	-2.0361	1.74E-09
Epha4	1218.758	1.2108	0.000139306	Arhgap10	215.362	-2.0389	0.00724401
Gata6	1582.668	1.2014	0.020028688	3110045C21Rik	61.440	-2.0467	0.020789776
Ly6g	574.137	1.1990	0.002012077	Ugt1a7c	2168.057	-2.0718	1.92E-05
Tle4	830.484	1.1987	0.000199721	Sytl4	126.776	-2.1443	0.002677615
Wls	2773.564	1.1872	0.024412673	Dnah2os	82.659	-2.1699	0.002162978
Evl	417.657	1.1841	0.005632033	Ripply3	79.970	-2.1949	0.041861089
Met	1175.914	1.1778	5.76E-07	Cdh5	401.295	-2.1979	1.82E-10
Ralgps2	3013.544	1.1476	8.15E-11	Ces2c	254.584	-2.2508	2.31E-05
Nbea	1074.953	1.1284	0.006333272	Gata4	2081.080	-2.2968	5.36E-09
Serpinb11	1597.953	1.1226	0.020948649	Khdrbs3	150.130	-2.3533	0.041877058
Sesn3	1609.353	1.1221	0.026168086	Cyp2c55	317.265	-2.4327	0.027809854
Gm8797	2848.687	1.1031	0.004043122	Epha3	149.757	-2.5006	0.010750799
Pdlim2	796.960	1.0960	0.004755074	Ifitm6	84.501	-2.5174	0.037116376
Pbx1	3957.006	1.0858	7.33E-05	Cep85	3224.738	-2.5444	4.51E-17
Mex3b	236.609	1.0814	0.022299697	Ihh	3761.400	-2.5703	1.56E-17
Npnt	2706.981	1.0667	2.17E-07	Adgrg2	170.771	-2.6128	0.008609952
Lmo7	6853.652	1.0329	8.40E-05	Glb1l2	42.797	-2.6280	0.041861089
Zfp618	734.995	1.0262	0.015051676	Ugt1a6a	1246.516	-2.7007	6.02E-05
Mfap3l	351.835	1.0158	0.01478608	Trim47	110.299	-2.7381	0.031486728
Arl5b	575.591	0.9980	0.029535976	Gm7357	64.423	-2.7593	0.004872248
Trio	1492.193	0.9917	0.000198067	Gabre	176.990	-2.7720	0.004446303
Gnai1	775.339	0.9905	0.02697543	Oprd1	73.005	-2.7929	0.01104423
Fam168a	1115.710	0.9826	0.000253632	Tmem266	1261.409	-2.8620	6.80E-09
Sema3c	4214.939	0.9820	0.000510395	Arg1	39.507	-2.9121	0.014368032
Itpkb	367.206	0.9769	0.03593397	Apoc2	59.957	-2.9698	0.008609952
Frk	1101.942	0.9758	0.028606055	Adh1	565.692	-3.0239	0.00017682
Ptk7	2294.898	0.9755	0.038582858	Fam183b	18.756	-3.1630	0.042716155
Serinc5	2956.797	0.9633	0.000989153	Akr1c14	66.426	-3.1630	0.004517555
Mcam	4053.665	0.9583	0.007841188	Gldc	38.050	-3.1831	0.036816825
Tgfbr1	1150.426	0.9428	0.010918488	Cyp2c65	286.693	-3.2278	7.22E-05
Mgat4c	1198.926	0.9413	9.01E-06	Gm14851	75.935	-3.2336	0.04771045
Dsp	16442.643	0.9273	3.43E-05	Cyp4b1	726.770	-3.2546	1.96E-06
Rgs12	1601.375	0.9150	0.024709039	Slc28a2	430.714	-3.2624	1.06E-17

Zfp26	471.377	0.9044	0.035705253
Peli1	863.636	0.9025	0.02141478
Pik3cb	939.780	0.8940	0.007127572
Zcchc11	983.508	0.8834	0.007438679
Cblb	428.432	0.8826	0.013754336
Fam83d	462.082	0.8799	0.025555099
Gp1bb	1250.692	0.8687	0.02857225
Atp11a	2297.482	0.8678	0.001309896
Ptprj	3344.064	0.8642	0.002424496
Lrp4	3121.582	0.8556	0.026951216
Btbd7	530.295	0.8427	0.045348902
Mbd5	355.041	0.8382	0.045155934
Nhsl1	2443.992	0.8274	0.003138478
Apaf1	1833.873	0.8269	0.000229283
Ptpre	1241.255	0.8163	0.00017682
Arid5b	1035.497	0.8140	0.013203002
Gpd2	4167.941	0.8070	2.84E-05
Pam	3607.388	0.7900	0.00702117
Cenpf	1858.433	0.7896	0.02408616
Grhl3	1997.402	0.7876	0.046874231
Wsb1	2356.787	0.7865	0.008320802
Foxa1	2979.478	0.7829	0.04771045
Notch2	1487.157	0.7827	0.010090541
Wipi1	1142.096	0.7775	0.017247803
Cdk17	656.058	0.7688	0.012053334
Tfip11	2752.693	0.7642	0.048431765
Farp1	1989.846	0.7497	0.005302767
Ptbp2	831.446	0.7494	0.024255873
Cobl	2719.000	0.7472	0.022622615
Trp53inp2	1819.459	0.7456	0.04771045
Jag1	1452.110	0.7341	0.032767491
Bcl9	1586.685	0.7316	0.002533876
Kitl	4642.004	0.7041	0.038002848
Afap1l1	5835.163	0.6953	0.049142352
Scpep1	2850.151	0.6916	0.001657384
Epc2	894.154	0.6909	0.040526834
Zfp703	6066.750	0.6892	0.047975601
Etl4	2288.212	0.6878	0.04148622
Jmjd1c	1522.959	0.6875	0.017395901
Adam10	4150.597	0.6834	0.020912016
Itga6	6482.326	0.6694	0.00029331
Nudt4	5160.221	0.6608	0.0295267
Fryl	2750.545	0.6502	0.006904233
Rnf38	2177.811	0.6082	0.036627682
Aff4	2951.017	0.6057	0.039242954
Lpgat1	2062.510	0.6047	0.020926743
Phactr4	1961.552	0.5829	0.032767491
Zdhhc21	1329.859	0.5713	0.045439476
Tmem245	1702.351	0.5654	0.022622615
Lrrc16a	2282.516	0.5601	0.047178664
Oxct1	5867.987	0.5593	0.017127546
Exoc6b	1550.392	0.5561	0.036816825
Traf6	1239.292	0.5518	0.044447523
Tes	4735.285	0.5420	0.022622615
Macf1	3543.230	0.5079	0.024255873
Anxa4	32898.066	0.4631	0.042305672

Ugt8a	111.884	-3.2883	0.003995557
Gm37788	23.518	-3.2988	0.041861089
Sult1c2	166.486	-3.3588	0.005632033
T	62.162	-3.4451	0.000111932
St3gal5	52.834	-3.4674	0.02314818
Prap1	811.752	-3.5589	2.38E-09
Aqp5	58.878	-3.6262	0.042414975
Tcf23	160.267	-3.6895	9.00E-05
Dmtn	53.711	-3.7410	0.041861089
Akp3	236.491	-3.7470	0.000115093
Clec2f	103.898	-3.7685	0.010219868
Anxa10	59.517	-3.8672	0.000664969
Prl2c3	137.452	-3.9051	0.008445669
Smoc1	109.444	-3.9095	0.02697543
Pcdhgb7	21.423	-4.0649	0.017290909
RP23-359B23.11	692.619	-4.2458	5.00E-60
Cd59a	27.208	-4.2570	0.0295267
Adh7	109.722	-4.5185	0.031808902
Olfml2b	274.122	-4.5860	9.80E-12
Myo7a	152.766	-4.7548	6.61E-11
Vsig2	77.927	-4.9937	1.82E-12
Tff2	602.197	-5.1415	0.009420265
Lipf	3117.599	-5.3635	0.005027141
Cfh	42.786	-5.6052	0.000194505
Rps4l	93.687	-5.6304	5.15E-06
Gm43305	26435.609	-5.9909	3.86E-05

Table S14, related to Figure 6D (middle panel).

List of the significantly up-regulated and down-regulated mRNAs in *Csf1r*-deficient tumoroids compared to *Apc*$^{Min/+}$ tumoroids.

Significantly up-regulated mRNAs				Significantly down-regulated mRNAs			
Gene symbol	Base mean	Log_2 fold change	padj	Gene symbol	Base mean	Log_2 fold change	padj
Eno1b	2115.547	8.545	0.005264977	*Taldo1*	12320.003	-0.552	0.006527818
Lipf	3117.599	5.033	0.032217819	*Slc12a8*	1296.432	-0.660	0.049604636
Eif5al3-ps	407.649	5.032	1.19E-11	*Cda*	919.923	-0.739	0.012908849
Reg3b	1578.899	4.797	0.010926405	*Gm26825*	61907.963	-0.768	0.000605867
Chd9	6859.116	4.488	0.00024203	*2810428I15Rik*	1027.442	-0.773	0.029781772
Nid2	70.791	4.246	0.008182885	*Septin5*	11872.794	-0.783	0.004852742
Ildr2	148.520	4.046	2.85E-05	*Bmp8b*	1501.902	-0.786	0.001513872
Nyap1	122.601	4.019	0.01115132	*Tmem180*	948.149	-0.813	0.037485635
Gm11942	32.764	3.965	0.005278321	*Phlda1*	12248.270	-0.890	0.000988203
Slco5a1	105.067	3.951	5.87E-10	*Rccd1*	1050.616	-0.892	0.001657048
Tmod2	291.368	3.857	0.000331018	*Mgst2*	1191.236	-0.912	0.017159244
Klhl13	105.530	3.856	7.07E-09	*Ldhd*	709.184	-0.919	0.013846932
Dio1	115.796	3.846	0.000782316	*Fhdc1*	744.435	-1.028	0.005452637
Trpm6	93.381	3.828	0.027130829	*Aim1l*	926.467	-1.042	0.005425664
Ackr3	147.564	3.643	0.022826051	*Eefsec*	834.524	-1.044	0.004060918
Enpp2	201.155	3.575	1.54E-09	*Gp1bb*	1250.692	-1.047	0.011612686
Gm10052	628.537	3.389	0.000307527	*Acsl1*	1160.101	-1.083	0.001623629
Gm28036	366.737	3.315	1.02E-05	*Cdhr2*	3046.022	-1.192	8.90E-05
1700003F12Rik	82.768	3.311	0.001158154	*Lrrc66*	631.904	-1.210	0.000971269
Gm5148	40.408	3.283	0.017159244	*Gm12744*	453.349	-1.218	0.003689542
Olfml2b	274.122	3.168	9.71E-06	*AI506816*	6888.954	-1.235	8.16E-11
Arhgap44	637.247	2.901	2.39E-12	*Galnt6*	637.388	-1.257	0.018379296
Dkk2	453.441	2.822	0.013846932	*Itln1*	2362.524	-1.293	5.43E-05
Ifit1	77.962	2.802	0.0079409	*Tm4sf5*	885.553	-1.336	0.005393062
Ccdc33	170.411	2.785	0.005262043	*3930402G23Rik*	132.081	-1.371	0.034288645
Cyp2f2	128.239	2.700	0.039165153	*Il3ra*	451.682	-1.477	0.000440972
Reg3g	983.822	2.668	4.13E-06	*Gm8355*	6799.964	-1.511	0.003618566
Plac9b	304.759	2.646	0.000156357	*Cwh43*	526.966	-1.613	0.014464497
Cd200	80.474	2.643	0.007590836	*Tm6sf2*	150.051	-1.642	0.013846932
Hip1	583.885	2.633	0.001285767	*2610528A11Rik*	283.786	-1.657	0.009428004
RP24-390A22.1	939.211	2.600	1.15E-06	*Fabp2*	693.495	-1.837	9.14E-06
2210418O10Rik	195.445	2.482	1.08E-05	*Ces2c*	254.584	-1.845	0.005262043
Rhbdl2	240.854	2.443	0.001158154	*2210407C18Rik*	2916.869	-1.920	0.021286363
Rarb	360.379	2.437	5.87E-10	*Oit1*	951.318	-1.954	0.001623629
Gm10073	349.913	2.394	9.49E-13	*Bcas1*	324.096	-2.000	2.87E-05
Htra1	58.451	2.382	0.019673334	*Prap1*	811.752	-2.067	0.013846932
Serpinb7	58.653	2.309	0.036645791	*Gm10036*	1791.573	-2.118	6.41E-06
Trib2	47.392	2.289	0.042319558	*Bnipl*	107.914	-2.215	0.041163186
RP23-359B23.11	692.619	2.217	3.03E-15	*Gsta1*	2811.844	-2.329	1.71E-09
Irx5	654.455	2.163	0.022448647	*Gm20699*	73.768	-2.389	0.001742256
Tbx3os1	128.414	2.091	0.000397301	*Trim29*	293.204	-2.443	0.019660527
Cep85	3224.738	2.087	4.88E-10	*Ereg*	168.653	-2.645	3.03E-05
Tmem254c	2306.541	2.075	0.019673334	*Gdpd3*	1422.754	-2.729	3.21E-08
Socs3	975.398	2.064	0.008239514	*4930452B06Rik*	125.072	-2.735	0.001158154
Tmem254b	675.334	1.988	0.009428004	*Gm37335*	96.624	-2.784	0.001847746
Slc4a3	136.680	1.985	0.000228556	*Rpgrip1*	85.153	-2.794	0.005262043
Tmem254a	700.793	1.951	0.005264977	*RP23-457I3.2*	223.702	-3.019	2.15E-05
Lrch4	467.626	1.858	0.001272615	*Gm26377*	2536.232	-3.124	0.045318862
Hspa8	35831.502	1.842	1.66E-11	*Gm8885*	55.853	-3.292	0.000152853
Jdp2	355.774	1.686	0.021552668	*Anxa10*	59.517	-3.568	0.008747472
Mtmr7	299.410	1.636	0.004774663	*Fut10*	72.175	-3.582	0.000458363
Rilpl1	176.654	1.612	0.006527818	*Gm5292*	199.915	-4.934	4.20E-30
Rpl11	2971.549	1.596	0.010208959	*Cyp2c29*	64.591	-5.655	0.000379312
Gm37376	1504.388	1.585	0.032217819	*Gm10704*	940.745	-5.777	1.18E-48
Rnf24	314.334	1.564	0.013846932	*Gm10020*	4645.692	-5.822	1.07E-06
Gm14226	548.793	1.556	0.015813486	*Gm14094*	315.824	-6.057	8.90E-39
Chac1	391.144	1.493	0.010749668	*Gm10093*	1986.903	-6.911	6.46E-120
Atp2b4	284.465	1.411	0.000417447	*Gm10182*	3021.766	-7.110	2.24E-08
Eno3	790.059	1.402	0.043479661	*Gm8420*	2800.915	-8.425	1.47E-132
Cdh5	401.295	1.402	0.001158154				
Ifi27	555.921	1.388	0.042022706				
Zfp532	391.439	1.387	0.039165153				
Smarca2	1084.414	1.379	0.009636562				
Plat	2312.407	1.343	0.005262043				
Gm4294	547.551	1.337	0.000704746				
Cmtm3	284.671	1.322	0.000873517				
Rpl15-ps3	8562.884	1.287	0.005262043				
Gm6206	321.412	1.193	0.019673334				
Asah1	3488.842	1.145	0.002067016				
Gpt2	530.673	1.140	0.012014694				
Sema6a	676.229	1.123	0.045072294				
Pisd-ps1	3559.954	1.001	0.011010573				
Tmem150a	308.229	0.983	0.026953258				
Bahcc1	2313.376	0.948	0.008239514				
Atp11a	2297.482	0.947	0.001207801				
Slc17a5	1027.990	0.944	0.013846932				
Vegfa	3634.098	0.873	0.005264977				
Cdh13	3191.281	0.847	1.05E-05				
Tbx3	5316.960	0.838	0.047066946				

Arid5b	1035.497	0.818	0.039845208
Large	840.153	0.808	0.012908849
Galnt11	639.879	0.766	0.01561526
Hsdl2	923.246	0.728	0.044984804
Cd24a	14100.979	0.700	0.001513872
Sap30	1461.658	0.611	0.039165153
Runx1	1436.232	0.600	0.046228427

Table S15, related to Figure 6D (lower panel).

List of the significantly up-regulated and down-regulated mRNAs in *Csf1r/Mir34a*-deficient tumoroids compared to *Apc*$^{Min/+}$ tumoroids.

Significantly up-regulated mRNAs				Significantly down-regulated mRNAs			
Gene symbol	Base mean	Log$_2$ fold change	padj	Gene symbol	Base mean	Log$_2$ fold change	padj
Gm10182	3021.766	5.666	1.59E-05	*Htra2*	1798.309	-0.555	0.041544
Gpc3	75.939	4.812	0.000112	*Gstm1*	6780.965	-0.737	0.005879
Zfp462	209.491	4.460	0.003653	*Cdhr2*	3046.022	-0.853	0.019161
Dkk2	453.441	4.338	2.64E-06	*Ccnd2*	19892.557	-0.945	0.034215
Cyp2f2	128.239	4.204	5.21E-06	*Camkk2*	451.907	-1.068	0.046562
Gm13067	24.628	4.064	0.038304	*1110028F11Rik*	595.344	-1.104	0.022368
Krt6a	622.517	4.048	1.96E-10	*Cth*	1414.812	-1.116	0.009021
Spink1	68.628	4.042	0.022515	*Gstm3*	679.570	-1.165	0.01257
Rtn1	64.786	3.964	0.010003	*Anpep*	720.996	-1.246	0.046199
Irx5	654.455	3.921	6.40E-09	*Slc40a1*	1100.126	-1.297	0.000394
Slc15a2	172.769	3.845	0.017163	*St3gal6*	1449.498	-1.331	0.022368
Gm29865	63.006	3.836	3.06E-05	*Apob*	1304.153	-1.396	0.005601
Fam43b	92.413	3.824	0.004269	*Galnt6*	637.388	-1.509	0.000836
Crnde	233.410	3.784	3.85E-05	*Gata4*	2081.080	-1.518	0.00226
Gm5148	40.408	3.779	0.000636	*Oit1*	951.318	-1.554	0.030775
Ncf2	100.728	3.490	0.004269	*Ereg*	168.653	-1.660	0.048455
Gm12669	217.052	3.296	0.038703	*Tm6sf2*	150.051	-1.763	0.004396
Cxcl14	54.669	3.273	0.011498	*Ces2c*	254.584	-1.865	0.003241
Gm45062	160.126	3.254	0.040073	*Acta1*	134.371	-1.908	0.000705
Car1	269.064	3.215	0.010679	*Ccdc141*	1178.872	-1.917	0.021743
Gpr157	136.064	3.093	9.85E-07	*Rpl11*	2971.549	-1.920	0.000275
RP23-457I3.2	223.702	3.069	6.07E-06	*Gsta1*	2811.844	-2.053	1.35E-07
Pcdh7	133.184	2.950	0.016184	*Cep85*	3224.738	-2.431	8.47E-15
RP24-390A22.1	939.211	2.943	2.34E-09	*Pla2g4a*	159.974	-2.454	0.014701
Lrrn3	81.537	2.893	0.008453	*Trf*	72.579	-2.613	0.016652
Dynap	78.475	2.826	0.009571	*Tgfb3*	91.135	-2.646	0.00226
Tbx1	2699.388	2.605	3.22E-06	*Muc6*	318.750	-2.668	2.33E-11
Irx3	66.315	2.550	0.009064	*Prap1*	811.752	-2.753	4.32E-05
RP23-145I16.5	350.152	2.527	0.047585	*Apoc2*	59.957	-2.838	0.0346
Enpp2	201.155	2.472	8.00E-05	*Tek*	324.310	-2.893	0.043686
Vim	3736.819	2.455	1.47E-05	*Plac9b*	304.759	-3.078	1.57E-06
Mreg	60.312	2.415	0.034215	*Dzip1l*	83.364	-3.099	0.032962
Gm20463	77.245	2.412	0.00226	*Tmem45a*	216.013	-3.181	0.014701
Serpinb7	58.653	2.350	0.019161	*Cybrd1*	219.553	-3.263	0.000187
Gm14226	548.793	2.265	4.27E-06	*Anxa10*	59.517	-3.278	0.016913
Gm25287	123.035	2.252	0.006312	*RP23-359B23.11*	692.619	-3.337	1.08E-39
Apcdd1	2075.022	2.251	0.000836	*Cfh*	42.786	-3.773	0.023116
St6gal1	576.819	2.239	8.45E-06	*Gkn3*	94.745	-4.141	0.016184
Htra1	58.451	2.203	0.04251	*Vsig2*	77.927	-4.546	1.25E-10
Znf41-ps	118.132	2.178	0.013182				
Col16a1	348.416	2.172	0.00226				
Rerg	130.070	2.087	0.030775				
Rassf4	342.221	2.008	0.046562				
Gm38394	961.573	1.891	0.035358				
Golga7b	475.245	1.871	0.000123				
Tgfbi	300.710	1.843	0.028586				
Gm22918	125.585	1.828	0.033746				
Rdh9	777.053	1.792	4.85E-09				
Tpbg	262.197	1.778	0.010003				
Gm13139	155.515	1.751	0.010003				
Reg3g	983.822	1.751	0.01436				
Tnfrsf11b	2360.995	1.656	1.76E-06				
Mtmr11	740.967	1.599	9.70E-08				
Ephb6	566.105	1.589	0.006291				
Hspb1	415.251	1.581	0.010679				
Tenm4	1017.163	1.485	0.009746				
Tbx3os1	128.414	1.481	0.035104				
Fbxo32	816.623	1.461	0.018058				
Casp12	234.405	1.424	0.030819				
Gm10036	1791.573	1.392	0.016913				
Slc16a10	682.693	1.267	0.018529				
Robo1	731.218	1.245	0.02306				
Tcf4	1757.854	1.241	0.016913				
Ptprd	2082.306	1.230	0.000836				
Dpysl3	1071.417	1.165	0.016184				
Mmp7	12865.237	1.131	0.000155				
Rarb	360.379	1.122	0.048455				
Zc3h12c	495.719	1.097	0.04301				
Itpkb	367.206	1.084	0.034215				
Bnip3	3814.478	1.077	0.017631				
Kitl	4642.004	0.985	0.001501				
Met	1175.914	0.720	0.0346				
Sap30	1461.658	0.639	0.018258				
Cdh13	3191.281	0.528	0.036342				
